# Nutritional interventions and dietary supplements in muscle diseases: a systematic review

**DOI:** 10.1093/rheumatology/keag378

**Published:** 2026-07-21

**Authors:** Taanya Talreja, Deepanjali Vedantam, Pranathi Bandarupalli, Lakshmi Nagendra, Sheryl Salis, Karen Cheng, Teerin Liewluck, Debra Lupeika, Ashley MacLean, Latika Gupta

**Affiliations:** Seth GS Medical College and King Edward Memorial Hospital, Mumbai, India; Shasta Regional Medical Centre, Redding, CA, USA; Mercy Health, Toledo, Ohio, USA; JSS Medical College, Mysore, India; Nurture Health Solutions, Mumbai, India; International Myositis Society, University Medical Center Göttingen, Göttingen, Germany; Department of Neurology, Mayo Clinic, Rochester, MN, USA; Department of Family and Community Medicine, University of California, Davis, CA, USA; Assistant Sports Dietitian, Stanford University, CA, USA; Department of Rheumatology, Royal Wolverhampton Hospitals NHS Trust, Wolverhampton, UK; School of Infection, Inflammation and Immunology, College of Medicine and Health, University of Birmingham, Birmingham, UK; Francis Crick Institute, London, UK

**Keywords:** diet, nutrition, myositis, muscular dystrophy, supplements

## Abstract

**Objectives:**

Medical nutrition therapy significantly impacts cardiovascular risk and overall health, but effects on muscle diseases remain unclear. This systematic review evaluates the safety and efficacy of dietary interventions and supplements on muscle disease outcomes.

**Methods:**

A multidisciplinary team conducted a PRISMA-guided systematic review registered on PROSPERO. Searches were conducted across multiple databases and screened against pre-specified inclusion criteria.

**Results:**

Of 107 full-text articles screened, 51 met inclusion criteria. Most identified interventions used dietary supplements rather than whole dietary approaches. In inflammatory myopathies, creatine (loading dose 20 g/day, maintenance 3 g/day) combined with exercise improved high-intensity functional performance in PM and DM over 6 months. In Duchenne muscular dystrophy, creatine (2–10 g/day for 8–16 weeks) improved maximal voluntary contraction and fatigue resistance. Carbohydrate-rich diets (65% CHO) reduced exercise-related symptoms in McArdle disease, while high-dose creatine (150 mg/kg/day) paradoxically worsened symptoms. Four trials of aceneuramic acid (6 g/day for 48 weeks) in GNE myopathy demonstrated dose-dependent strength improvements, leading to regulatory approval in Japan. High-protein supplementation showed positive trends for muscle preservation in critical illness myopathy. Quality assessment revealed 31% at low risk of bias, 49% with some concerns and 20% at high risk.

**Conclusion:**

Evidence for nutritional interventions in muscle diseases remains limited, especially for inflammatory myopathies. The strongest support emerged for mechanistically targeted approaches: creatine with exercise, carbohydrate-rich and ketogenic diets in McArdle disease and sialic acid in GNE myopathy. Future research requires adequately powered multicentre trials with standardized outcomes, with focus on inflammatory myopathies.

## Graphical abstract

**Graphical Abstract:**
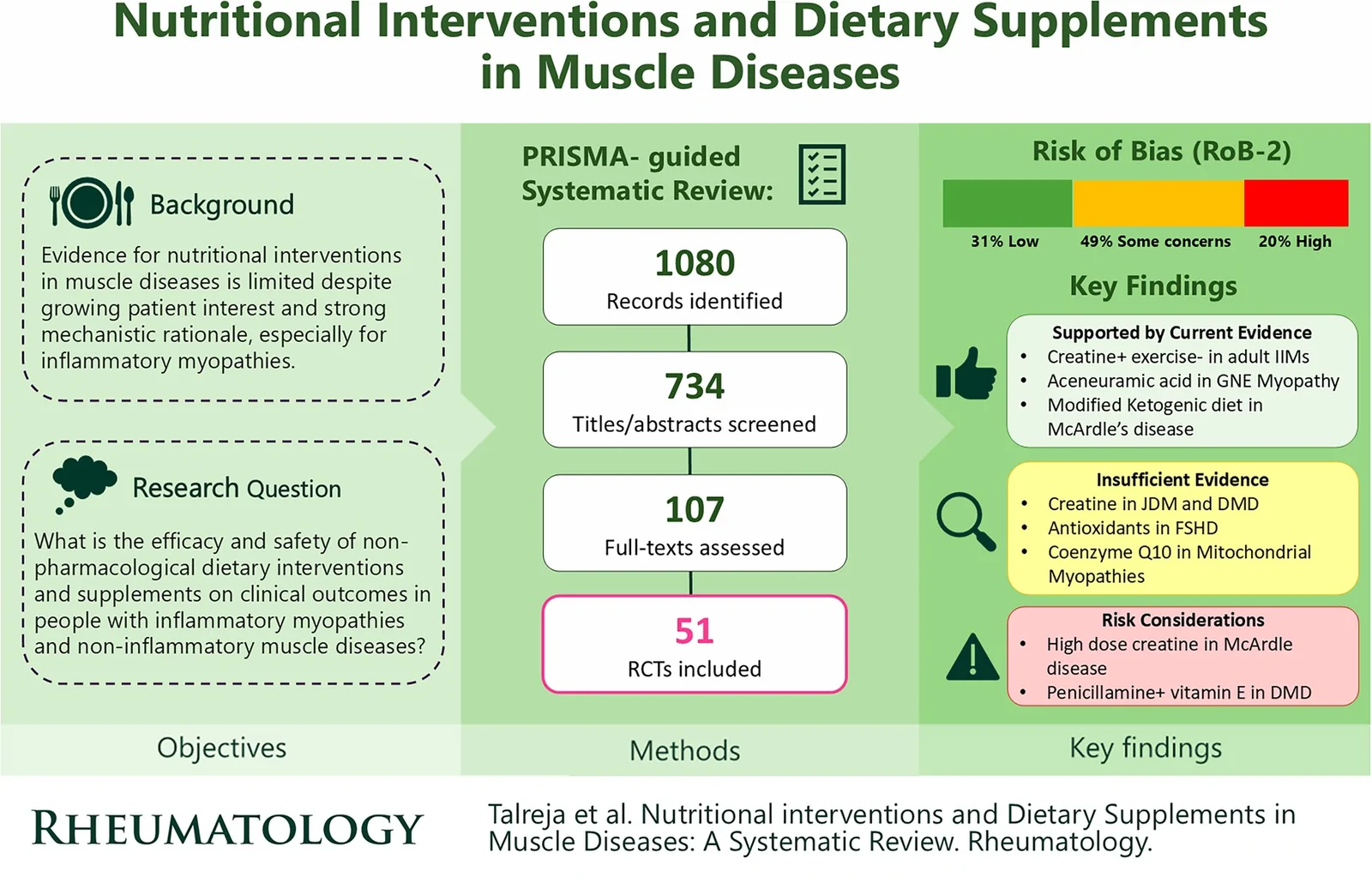

*Rheumatology* key messages

- Evidence for nutritional interventions in muscle diseases remains limited, particularly for inflammatory myopathies.
- Creatine supplementation with exercise shows promise in inflammatory myopathies and some muscular dystrophies.
- Mechanistically targeted approaches like sialic acid in GNE myopathy demonstrate disease-specific therapeutic potential.

## Introduction

Medical nutritional therapy increasingly influences disease outcomes, with tailored dietary interventions improving health across diverse conditions [1]. Food-based strategies, such as the Mediterranean diet [2], dietary approaches to stop hypertension (DASH) diet [3] and plant-based diets [4] have demonstrated substantial benefits in reducing cardiovascular events and improving overall health [5]. Among rheumatic diseases, high-dose omega 3 and vitamin D supplementation, plant-based diets and the Mediterranean diet have shown particular promise, with the most robust evidence emerging from RA research [6–8].

Idiopathic inflammatory myopathies (IIMs) affect 2–25 per 100 000 people [9], while muscular dystrophies affect ∼20–25 per 100 000 individuals [10], collectively impacting hundreds of thousands of patients worldwide. Current therapeutic options are limited: immunosuppressive therapies require prolonged treatment regimens accompanied by substantial adverse effects [11], while the absence of definitive cures for muscular dystrophies and metabolic myopathies restricts treatment options to supportive care and symptomatic management. Patients increasingly seek nutritional guidance through online communities and social platforms [12], highlighting a critical opportunity for physicians to address nutrition more systematically. Moreover, the proliferation of dietary trends and supplement marketing poses a significant challenge, as both healthcare providers and patients struggle to distinguish evidence-backed interventions from unsubstantiated claims. Thus, the potential for nutrition to serve as an accessible, affordable and low-risk adjunctive therapy makes this research gap increasingly important to address.

The intricate relationship between nutrition and immune function is well-established [13], with dietary therapeutic approaches demonstrating potential to modulate disease activity in various IIMs [14]. Oxidative stress, a hallmark of inflammatory, metabolic and degenerative diseases, drives contractile derangements in muscular dystrophies [15], while mitochondrial dysfunction can be targeted through interventions such as ketogenic diets and branched chain amino acids (BCAA) that enhance mitochondrial biogenesis [16]. Altered protein turnover in muscle diseases [17, 18] suggests potential benefits from optimized protein intake for muscle protein synthesis and preservation of muscle mass. Despite this strong mechanistic foundation and evidence from related conditions, the specific role of nutritional interventions in muscle diseases remains largely unexplored. This situation is compounded by the rarity of individual conditions, which makes conducting adequately powered nutritional studies challenging [19].

This systematic review addresses this critical knowledge gap by comprehensively evaluating the safety and efficacy of non-pharmacological dietary interventions in people with acquired myopathies, both inflammatory and non-inflammatory subtypes, and inherited myopathies. By synthesizing the available evidence across these related but distinct conditions, this review aims to establish the current state of knowledge regarding nutritional interventions in muscle diseases and identify priorities for future research and establishment of guidelines. Understanding and exploring such nutritional approaches can have implications transferable to several other inflammatory and metabolic conditions.

## Methods

### Design

This systematic review was conducted in accordance with the Cochrane Handbook [20] and reported following the PRISMA guidelines [21]. The protocol was prospectively registered with PROSPERO (CRD420251031110). A multidisciplinary expert panel comprising a rheumatologist, endocrinologist, neurologist, nutritionist, dietitian and patient research partner living with muscle disease collaboratively developed the research question and methodology.

The research question was: ‘What is the efficacy and safety of non-pharmacological dietary interventions and supplements on clinical outcomes in people with inflammatory myopathies and non-inflammatory muscle diseases?’

Study selection was guided by a structured PICO framework (Population, Intervention, Comparison, Outcome) [20]. Eligible studies included participants of all ages, encompassing both adult and paediatric populations, with confirmed diagnoses of inflammatory myopathies or non-inflammatory muscle conditions. Interventions comprised dietary modifications, nutritional supplements, fasting regimens or lifestyle factors [7]. Comparators included placebo, usual diet, standard care, alternative dietary interventions or different doses/regimens of the same intervention. Outcomes encompassed disease activity measures, organ-specific assessments, body composition, biomarkers, imaging or electrophysiological assessments and patient-reported outcomes. Only randomized controlled trials (RCTs) were eligible, providing the highest level of evidence [22].

Exclusion criteria comprised review articles, editorials, conference abstracts, case reports, case series and animal/*in vitro* studies. Detailed inclusion and exclusion criteria are provided in Supplementary Data S1.

### Search strategy

Systematic searches were performed in MEDLINE (via PubMed), Embase and the Cochrane Library from inception through 25 April 2025. Studies published in English or translated to English were considered. Complete search strategies are provided in Supplementary Data S2. Supplementary searches included reference list screening of included studies and relevant systematic reviews, forward citation searching and examination of clinical trial registries (https://clinicaltrials.gov).

All identified citations were uploaded to Covidence systematic review software and duplicates removed. Two reviewers (T.T., D.V.) independently screened titles and abstracts against predefined eligibility criteria. Full-text articles were retrieved for all studies meeting inclusion criteria or having sufficient information to assess eligibility, and were independently examined by two reviewers (T.T., D.V.). Disagreements at any stage were resolved through discussion, with adjudication by a fourth reviewer (L.G.) when consensus could not be reached.

### Data extraction and synthesis

Two independent reviewers (D.V., T.T.) extracted data using a standardized data extraction form [19], with all extracted data cross-verified to ensure accuracy and consistency. Discrepancies were resolved through discussion, with adjudication by a third reviewer (L.G.) when necessary. Extracted data included: study characteristics (author, year, country, design); participant characteristics (inclusion/exclusion criteria, disease type, age, sample sizes); intervention details (type, dose, duration); comparator details; outcome measures and tools; follow-up period and adverse events. Studies were organized by condition type and intervention category, with study characteristics, intervention details and outcomes tabulated for each intervention.

### Risk of bias assessment

Two independent reviewers (D.V., T.T.) assessed risk of bias using the Cochrane Risk of Bias 2 (RoB 2) tool for randomized trials [23]. Disagreements were resolved through discussion with a fourth reviewer (L.N.) when necessary. Studies were rated as ‘low risk of bias’, ‘some concerns’ or ‘high risk of bias’ according to RoB 2 criteria, with summary figures generated.

## Results

**Figure 1:**
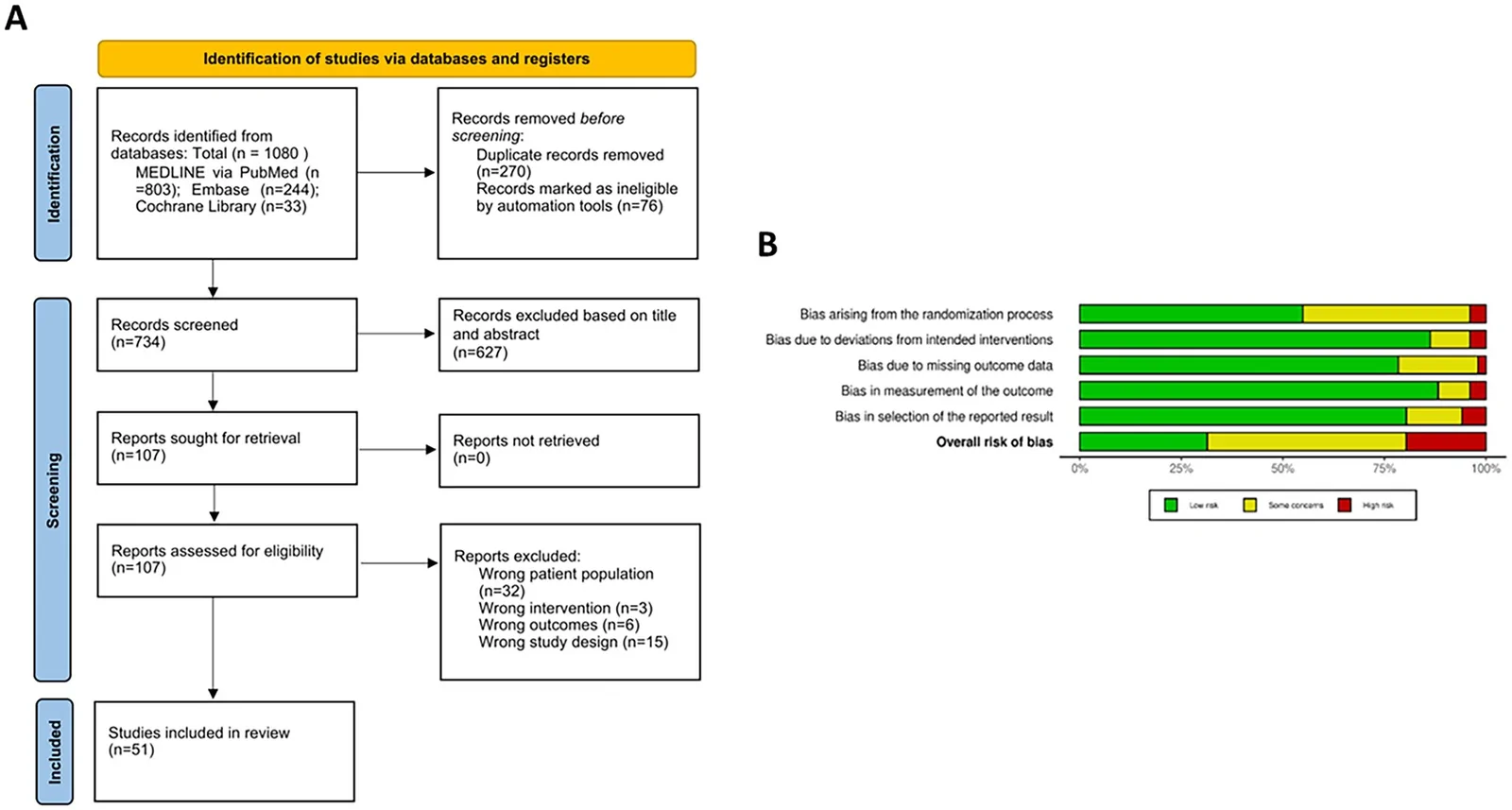
(**A**) PRISMA workflow chart for included studies, (**B**) risk of bias of included studies

The search identified a total of *n* = 1080 records. After removal of duplicates, 734 titles and abstracts were screened, and 107 full-text articles were assessed for eligibility. Of these, 51 studies met the inclusion criteria and were included in the review (Fig. 1A).

### Risk of bias assessment

Overall, 16 studies (31%) were at low risk of bias, 25 (49%) had some concerns and 10 (20%) were at high risk (Fig. 1B, Supplementary Fig. S1). The most common methodological concerns were inadequate reporting of randomization procedures and allocation concealment (Domain 1), affecting approximately half of the studies, while a smaller proportion had problems with missing outcome data (Domain 3).

### Characteristics of included studies

The included trials comprised 27 parallel-group RCTs (54%) and 24 crossover RCTs (46%). Two studies utilized open-label designs [24, 25]. Studies spanned five major domains: inflammatory myopathies, muscular dystrophies, metabolic and mitochondrial myopathies, critical illness myopathy and distal myopathies.

Interventions across the 51 trials included diverse nutritional approaches. Creatine supplementation was the most studied (*n* = 12), followed by dietary modifications (ketogenic/high-carbohydrate/high-protein diets), sialic acid-based therapies, ketone esters, omega-3 fatty acids, β-hydroxy-β-methylbutyrate (HMB), glutamine, antioxidant combinations, Coenzyme Q10 (CoQ10), L-carnitine, resveratrol, and single studies of branched chain amino acids (BCAAs), leucine, L-citrulline + metformin, cysteine donor and penicillamine + vitamin E.

Outcome measures demonstrated substantial heterogeneity. Muscle strength and functional capacity were assessed using diverse tools (manual muscle testing, dynamometry, 6-min walk test, timed function tests). Biochemical markers (creatine kinase, lactate, liver enzymes), patient-reported outcomes (SF-36, disease-specific scales) and advanced measures (P^31^Magnetic Resonance Spectroscopy, muscle MRI, cardiopulmonary testing) were variably reported. Standardized core outcome sets (IMACS, PRINTO) were rarely employed, contributing to heterogeneity that precluded meta-analysis. Complete details of all included studies are in Supplementary Data S3.

### Inflammatory myopathies

Four trials evaluated nutritional interventions in inflammatory myopathies, with creatine being the predominant intervention (*n* = 3, 75% of studies) (Table 1). Chung *et al.* [28] studied adults with PM or DM (median disease duration ∼9 years) with clinically stable, low disease activity who had received systemic steroid therapy for at least 6 months with stable prednisolone doses for 2 months. High-dose creatine (20 g/day loading for 8 days, then 3 g/day maintenance) combined with home exercise over 6 months significantly improved the primary outcome of aggregate functional performance time (median decrease 13%, *P* = 0.014), as well as secondary outcomes of Functional Index of Myositis (median increase from 50.3 to 57.0, *P* = 0.034), manual muscle testing (MMT) scores for shoulder abduction and hip flexion (*P* ≤ 0.05) and phosphocreatine/β-nucleoside triphosphate (PCr/β-NTP) ratios (3.4% increase, *P* = 0.05).

**Table 1:** Intervention efficacy in inflammatory myopathies.

Study	Disease; *N* (study/control)	Intervention & dose	Duration; adherence	Outcome—improvement	Outcome—no change
Low risk of bias					
Solis *et al.*, 2016 [26]	JDM; 15 (crossover)	Creatine monohydrate 0.1 g/kg/day	12 weeks (+8-week washout); not reported	–	Primary: muscle function and strength (1-RM, timed tests, MMT, CMAS) Secondary: muscle metabolism (^31^P-MRS PCr content), muscle strength (CMAS, MMT), disease activity (DAS, VAS), functional capacity (CHAQ), QoL (PedsQL), body composition and bone health (DXA, CTX, P1NP), aerobic capacity (VO_2_max, treadmill test), inflammatory markers (IL-6, IL-10, IL-17A, TNF-α, IFN-γ)
Kimura *et al.*, 2022 [27]	PM & DM; 24/23	Branched chain amino acids (BCAA): TK-98 (4.15 g) six packages/day in three divided doses	12 weeks; not reported	Secondary: functional capacity (FI score for bilateral shoulder flexion and all dynamic repetitive muscle functions)	Primary: muscle strength (MMT) Secondary: disease activity (MDACS)
Some concerns					
Chung *et al.*, 2007 [28]	PM & DM; 19/18	Creatine monohydrate (oral, highly purified) Loading: 20 g/day × 8 days Maintenance: 3 g/day + home exercise programme 5 days/week (both the groups)	6 months; no evidence of poor compliance	Primary: physical function (AFPT): median decrease of 13% in creatine group vs increase in placebo group, *P* = 0.014 Secondary: functional capacity (FI of myositis), muscle strength (MMT for shoulder abduction and hip flexion), muscle metabolism (PCr/β-NTP ratios)	Secondary: QoL (NHP), pain (short form McGill pain questionnaire), mood (hospital anxiety and depression scales), fatigue (Chalder fatigue score)
Dover *et al.*, 2021 [29]	JDM; 13 (multiple-baseline)	Creatine monohydrate 150 mg/kg/day chewable, lemon-flavoured tablets (AlzChem)	4 weeks–6 months; median adherence 88.5%	Primary: feasibility: adherence 88.5%; no missed visits; no adverse events Secondary: muscle metabolism (Pi/PCr ratio, pH recovery after exercise)	Primary: muscle function and strength (Wingate, maximal jump, handgrip) Secondary: aerobic capacity (YMCA cycle test), disease activity (IMACS), fatigue (PedsQL), physical activity (3DPAR), QoL (QoML)

Abbreviations: AFPT, aggregate functional performance time; BCAA, branched chain amino acids; CHAQ, childhood health assessment questionnaire; CMAS, childhood myositis assessment scale; CTX, C-terminal telopeptide of type I collagen; DAS, disease activity score; DXA, dual-energy X-ray absorptiometry; FI, functional index; IMACS, International Myositis Assessment and Clinical Studies; MDACS, myositis disease activity core set; MMT, manual muscle testing; MRS, magnetic resonance spectroscopy; NHP, Nottingham Health Profile; P1NP, procollagen type 1N-terminal propeptide; PCr/β-NTP, phosphocreatine/β-nucleoside triphosphate; PedsQL, paediatric quality of life inventory; Pi/PCr, inorganic phosphate/phosphocreatine; QoL, quality of life; QoML, quality of my life; 1-RM, one repetition maximum; TK-98, branched chain amino acid formulation; VAS, visual analogue scale; YMCA, YMCA cycle ergometer submaximal test; 3DPAR, 3-day physical activity recall; ^31^P-MRS, phosphorus-31 magnetic resonance spectroscopy.

Creatine supplementation in JDM yielded mixed results. Dover *et al.* [29] studied children with clinically stable JDM (disease duration 5–6 years) on stable medications using 0.15 mg/kg/day creatine, demonstrating good feasibility (primary outcome) and improved muscle metabolism (decreased Pi/PCr ratio, *P* = 0.03; reduced pH change after exercise, *P* = 0.003), but no improvements in muscle function or strength. Solis *et al.* [26] found no significant improvements in the primary outcome of muscle function assessed by strength and timed function tests; or any secondary measures including MMT and Childhood Myositis Assessment Scale (CMAS) scores, in children with JDM (mean disease duration 7 ± 3 years; 5 with active disease and 10 with inactive disease) on stable immunosuppression (≥8 weeks) receiving 0.1 g/kg/day creatine over 12 weeks.

BCAA supplementation was evaluated by Kimura *et al.* [27] in adults with PM or DM with relatively early disease (mean disease duration ∼6 months). BCAA supplementation (TK-98) over 12 weeks showed no change in the primary outcome of MMT scores, *P* = 0.98 but significantly improved functional index scores for bilateral shoulder flexion (*P* < 0.05) and all dynamic repetitive muscle functions, without affecting disease activity.

### Muscular dystrophies

In Duchenne muscular dystrophy (DMD), three trials evaluating creatine reported improvements in cellular energetics, strength measures, and bone mineral density [30–32], while one showed no significant functional benefits [33]. Omega-3 supplementation significantly reduced inflammatory markers in DMD [34], antioxidant supplementation improved quadriceps strength and endurance in FSHD at low risk of bias [35]. Details of all muscular dystrophy trials are reported in Table 2.

**Table 2:** Intervention efficacy in muscular dystrophies and distal myopathies.

Study	Disease; *N* (study/control)	Intervention & dose	Duration; adherence	Outcome—improvement	Outcome—no change
Muscular dystrophies					
Low risk of bias					
Escobar-Cedillo *et al.*, 2013 [36]	DMD *n* = 20 (10/10)	L-carnitine 50 mg/kg twice daily	12 months; not reported	–	Primary: muscle strength (MMT); functional status (Brooke, Swinyard, Vignos scales)
Escolar *et al.*, 2005 [33]	DMD *n* = 50 (creatine = 15, glutamine = 19, placebo = 16)	Arm 1: creatine monohydrate 5 g/day Arm 2: glutamine 0.3 g/kg twice daily	6 months; four withdrawals due to medication noncompliance	Secondary: muscle deterioration (QMT): less in creatine group; timed stair climbing: creatine better than placebo	Primary: muscle strength (MMT) Secondary: timed running; pulmonary function
Mendell *et al.*, 1984 [37]	DMD *n* = 91 (47/44)	L-leucine 0.1 g/kg twice daily	12 months; over 80% of the patients maintained acceptable levels of compliance	–	Primary: muscle strength (MMT); joint contractures; timed functional tests; pulmonary function (FVC, MVV, MEP); serum CK
Mok *et al.*, 2009 [38]	DMD *n* = 30 (crossover)	Glutamine 0.5 g/kg/day	4 months each (+1-month washout); good compliance	Secondary: fat-free mass increase attenuated with glutamine	Primary: gait speed Secondary: 2MWT; muscle mass; body composition; serum CPK
Passerieux *et al.*, 2013 [39]	FSHD *n* = 53 (26/27)	Antioxidant supplement vitamin C 500 mg + vitamin E 400 mg + zinc gluconate 25 mg + selenomethionine 200 μg/day	17 weeks; 96% adherence in supplemented group, 100% in placebo group	Primary: quadriceps strength (MVCQD): *P* = 0.011; quadriceps strength (MVCQND): *P* = 0.004; endurance (TlimQD): *P* = 0.028; endurance (TlimQND): *P* = 0.011 Secondary: Antioxidant markers (Vit C, Vit E, lipid peroxides): improved.	Primary: walking distance (2MWT): improved from baseline but no between-group difference
Rodríguez-Cruz *et al.*, 2018 [34]	DMD *n* = 40 (18/22)	Omega-3 LC-PUFAs 2.9 g/day EPA 45 mg + DHA 225 mg per capsule (×10 capsules/day)	6 months; 97.4% adherence in supplement group, 95.3% in control group	Primary: inflammatory markers: NF-κB, IL-1β, IL-6 mRNA downregulated; *P* < 0.05; serum IL-1β − 59.5%; *P* = 0.011; serum IL-6 − 54.8%; *P* = 0.041; serum IL-10 + 99.9%; *P* < 0.005	–
Schneider-Gold *et al.*, 2003 [40]	DM2/PROMM *n* = 20 (10/10)	Creatine monohydrate 10 g/day	3 months; 100% completion rate	Secondary: daily activity (VAS)	Primary: muscle strength (MRC, dynamometry) Secondary: NSS; grip strength
Sitzia *et al.*, 2019 [41]	DMD, FSHD & LGMD *n* = 29 (15/14)	Flavomega (*Curcumin* + acetyl-L-carnitine + CoQ10 + *Scutellaria baicalin* + green tea + fish oil + Vit E)	24 weeks; not reported.	Primary: isokinetic knee extension (Biodex), LGMD & FSHD only: *P* = 0.039 Secondary: walking distance (6MWT), LGMD & FSHD only; serum CK (DMD only): decreased	Secondary: questionnaire data (EK, ACTIVLIM, ABILHAND)
Some concerns					
Andersen *et al.*, 2015 [42]	FSHD *n* = 41 (18 training + protein, 13 training + placebo, 10 control)	Whey protein-carbohydrate supplement + aerobic training whey 23 g + carbohydrate 17 g post-exercise (vs placebo supplement + training vs no intervention)	12 weeks; supplement group: three patients missed one, three, five drinks, respectively; placebo group: two patients missed two and five drinks, respectively	Primary: exercise alone improved VO_2_ max (*P* < 0.002), workload (*P* < 0.001), walking speed (6MWT, *P* < 0.001). Supplement did not add benefit beyond training	Primary: protein supplement vs training alone: VO_2_ max; workload; walking speed; muscle strength; fatigue; daily activity level
Banerjee *et al.*, 2010 [30]	DMD *n* = 33 (18/15)	Creatine monohydrate 5 g/day (three divided doses)	8 weeks; one noncompliant patient.	Primary: bioenergetics (^31^P-MRS, PCr/Pi): *P* = 0.03 Secondary: muscle strength (MMT), parent-reported improvement	Secondary: functional status (Vignos scale)
Davidson *et al.*, 2021 [43]	DMD *n* = 36 (crossover)	Enhanced supplement creatine 5 g + glutamine 0.6 g/kg FFM + HMB 38 mg/kg + whey 22 g + vitamin D 2000 IU + fish oil 1050 mg Standard supplement whey 22 g + vitamin D 2000 IU + fish oil 1050 mg	20 weeks each (+2-week washout); proportion of used tablets: 85–88%	–	Primary: ambulation (6MWD, StepWatch) Secondary: body composition; QoL (PedsQL)
Fenichel *et al.*, 1988 [35]	DMD *n* = 106 (52/54)	Penicillamine + Vitamin E penicillamine: 125–500 mg/day; vitamin E: 600–1200 mg twice daily (dose by weight)	18 months; not reported	–	Primary: muscle strength; functional grade; timed tests; pulmonary function; serum CK
Louis *et al.*, 2003 [31]	DMD & Becker MD *n* = 15 (crossover)	Creatine monohydrate 3 g/day	3 months each (+2-month washout); not reported	Primary: muscle strength (MVC) *P* = 0.02; fatigue resistance: *P* = 0.001; BMD (ambulant patients): *P* < 0.05	Primary: joint stiffness (TJS); muscle bioenergetics (PCr/ATP ratio)
Tarnopolsky *et al.*, 2004 [44]	DM1 *n* = 42 (crossover)	Creatine monohydrate 5 g/day	4 months each (+6-week washout); not reported	–	Primary: muscle strength (MMT); body composition; bioenergetics (PCr/B-ATP) Secondary: isometric strength; pulmonary function; functional tests; ADL
Tarnopolsky *et al.*, 2004 [32]	DMD *n* = 30 (crossover)	Creatine monohydrate 2–5 g/day (weight-based)	4 months each (+6-week washout); not reported	Primary: handgrip strength (dominant hand): *P* < 0.05; FFM: *P* < 0.05; bone resorption (N-telopeptide/creatinine): reduced; *P* < 0.05	Primary: pulmonary function (FVC, FEV1), functional tasks, ADL
Walter *et al.*, 2000 [45]	Mixed MD (FSHD, Becker, DMD, LGMD) *n* = 32 (crossover)	Creatine monohydrate adults: 10 g/day; children: 5 g/day	8 weeks each (+3-week washout); not reported	Primary: muscle strength (MRC): *P* < 0.05; neuromuscular symptoms (NSS): *P* < 0.05; patient-assessed improvement: *P* < 0.05	Primary: pulmonary function (VC); laboratory tests
Walter *et al.*, 2002 [46]	DM1 *n* = 34 (crossover)	Creatine monohydrate loading: 10.6 g/day × 10 days; maintenance: 5.3 g/day	8 weeks each (+6-week washout); not reported	Note: in second crossover period only, strength and NSS improved	Primary: muscle strength (MRC, QMT); NSS; body composition; pulmonary function
Wilson *et al.*, 2024 [47]	FSHD *n* = 20 (10/10)	Antioxidant supplement vitamin C 500 mg + vitamin E 400 mg + zinc gluconate 25 mg + selenomethionine 200 μg/day	17 weeks; not reported	Primary: quadriceps strength (MVCQD): *P* = 0.014; quadriceps strength (MVCQND): *P* = 0.042 Secondary: muscle quality (MRI); physical function (SF-36); antioxidant markers	–
High risk of bias					
Hafner *et al.*, 2019 [48]	DMD *n* = 47 (23/24)	L-citrulline + metformin L-citrulline 2500 mg + metformin 250 mg, 3×/day	26 weeks; not reported	Primary: motor function (MFM D1), stable subgroup only: decline reduced, *P* = 0.03 Secondary: muscle fat fraction (MRI), muscle T2 relaxation time	Primary: motor function (MFM D1) Secondary: MFM D2, D3; timed tests; muscle force; lab biomarkers
Orndahl *et al.*, 1984 [49]	Myotonic dystrophy *n* = 27 (13/14)	Selenium + vitamin E titrated 0.4 → 1.6 mg/day selenium + 200 → 800 mg/day vitamin E	2 years; only 16/27 patients showed complete compliance	Primary: knee flexion strength (cybex, 120°/s); *P* < 0.05	Primary: handgrip strength; walking speed (declined in both the groups) Secondary: cognition (MMSE, SRB-3); functional status
Distal myopathies					
Low risk of bias					
Suzuki *et al.*, 2023 [50]	GNE myopathy; 19 (study *n* = 15; control *n* = 4)	Aceneuramic acid (SA-ER) four tablets of 500 mg thrice daily = 6 g/day	48 weeks; compliance > 70%	Primary: UEC at week 48: *P* = 0.0013 Secondary: shoulder abductor strength	Primary: UEC (Covariance analysis) Secondary: LEC; sit-to-stand; WAL; knee extensors; GNEM-FAS mobility and UE domains; 6MWT declined
Some concerns					
Argov *et al.*, 2016 [51]	GNE myopathy; 47	Aceneuramic acid (Ace-ER) 6 g/day *n* = 15; 3 g/day *n* = 18; Placebo *n* = 14; cross-over phase: 3 g *n* = 23, 6 g *n* = 24	48 weeks; not reported	Primary: UEC strength: 6 g/day vs placebo week 24: L *P* = 0.040; combined 6 g vs 3 g week 48: *P* = 0.0031; ≥200 m subgroup week 24: *P* = 0.040; week 48: *P* = 0.0005. Secondary: GNEM-FAS: trend improvement in UE function and mobility (6 g vs 3 g); EIM: dose-dependent impedance changes at week 24 (6 g group); IBMFRS and INQoL: dose-dependent positive trends at week 48	Primary: LEC, 6MWT, gait speed, stair climb, sit-to-stand, WAL, muscle MRI
Park *et al.*, 2023 [52]	GNE myopathy; 14	6′-Sialyllactose (6SL) (6 g/day *n* = 7; 3 g/day *n* = 7)	96 weeks; 100% completion	Primary: LEC score: high-dose vs low-dose, *P* = 0.0406; shoulder abduction *P* < 0.05; elbow flexion *P* = 0.038; hip flexion *P* = 0.0015; ankle dorsiflexion *P* = 0.0021 Secondary: Muscle fat fraction increase on MRI attenuated in high-dose group	Primary: UEC, 6MWT Secondary: GNEM-FAS
High risk of bias					
Mori-Yoshimura *et al.*, 2023 [53]	GNE myopathy; 14 (study *n* = 10; control *n* = 4)	Aceneuramic acid (SA-ER) four tablets of 500 mg three times daily = 6 g/day	48 weeks; 100% completion	Secondary: Investigator efficacy rate; GNEM-FAS UE and mobility scores	Primary: UEC Secondary: knee extensor strength

Abbreviations: ABILHAND, ability of hand scale; ACTIVLIM, activity limitation scale; ADL, activities of daily living; ATP, adenosine triphosphate; BMD, bone mineral density; CK, creatine kinase; CPK, creatine phosphokinase; DHA, docosahexaenoic acid; DM1/DM2, myotonic dystrophy type 1/2; DMD, Duchenne muscular dystrophy; EIM, electrical impedance myography; EK, Egen Klassifikation scale; EPA, eicosapentaenoic acid; FFM, fat-free mass; FLAVOMEGA, multicomponent supplement (curcumin, acetyl-L-carnitine, CoQ10, baicalin, green tea, fish oil, vitamin E); FEV1, forced expiratory volume in 1 s; FSHD, facioscapulohumeral muscular dystrophy; FVC, forced vital capacity; GNE, glucosamine (UDP-N-acetyl)-2-epimerase/N-acetylmannosamine kinase; GNEM-FAS, GNE myopathy functional activity scale; HMB, β-hydroxy β-methylbutyrate; IBMFRS, Inclusion Body Myositis Functional Rating Scale; LC-PUFA, long-chain polyunsaturated fatty acid; LEC, lower extremity composite score; LGMD, limb-girdle muscular dystrophy; MD, muscular dystrophy; MEP, maximum expiratory pressure; MFM, motor function measure (D1/D2/D3, dimensions 1/2/3); MMSE, mini mental state examination; MMT, manual muscle testing; MRC, medical research council scale; MRS, magnetic resonance spectroscopy; MVC, maximal voluntary contraction; MVCQD/MVCQND, maximal voluntary contraction of quadriceps (dominant/non-dominant); MVV, maximum voluntary ventilation; NF-κB, nuclear factor kappa B; NSS, neuromuscular symptom score; PCr, phosphocreatine; PedsQL, paediatric quality of life inventory; PROMM, proximal myotonic myopathy; QMT, quantitative muscle testing; SA-ER, sialic acid extended release; SF-36, short form-36 health survey; SRB-3, Stanford rating scale; TJS, total joint stiffness; TlimQD/TlimQND, endurance limit time of quadriceps (dominant/non-dominant); UE, upper extremity; UEC, upper extremity composite score; VAS, visual analogue scale; VC, vital capacity; WAL, weighted arm lift test; 2MWT, 2-min walk test; 6MWT, 6-min walk test; 6MWD, 6-min walk distance; ^31^P-MRS, phosphorus-31 magnetic resonance spectroscopy.

### Distal myopathies

Four trials of sialic acid-based therapies in GNE myopathy demonstrated dose-dependent strength improvements. Aceneuramic acid 6 g/day maintained upper extremity strength vs placebo decline over 48 weeks in three studies [50, 51, 53], while 6′-sialyllactose 6 g/day improved lower extremity strength and reduced muscle fat accumulation on MRI [52] (Table 2).

### Metabolic and mitochondrial myopathies

Across metabolic and mitochondrial myopathies, high-protein diet combined with exercise consistently improved aerobic capacity and quality of life in both adult and paediatric Pompe disease at low risk of bias [54, 55]. High-protein diet also maintained lean body mass in long-chain fatty acid oxidation disorders [56]. Details of all metabolic and mitochondrial myopathy trials are reported in Table 3.

**Table 3:** Intervention efficacy in metabolic and mitochondrial myopathies and critical illness myopathies.

Study	Disease; *N* (study/control)	Intervention & dose	Duration; adherence	Outcome—improvement	Outcome—no change
Metabolic and mitochondrial myopathies					
Low risk of bias					
Chen *et al.*, 1997 [57]	Mitochondrial encephalomyopathies (MERRF, MELAS, CPEOM) *n* = 8/8	Coenzyme Q10 (CoQ10) 160 mg/day orally	3 months; Not reported	Primary: muscle strength (MRC): improved; *P* < 0.05 Secondary: serum CoQ10 levels	Primary: ADL (subjective improvement; did not reach significance) Secondary: serum lactate and pyruvate after exercise
Gimenes *et al.*, 2015 [58]	Mitochondrial myopathy (CPEO) *n* = 6/6	L-carnitine 3 g/day once daily	16 weeks; 100% compliance	Primary: exercise tolerance (CWR Tlim), *P* < 0.05: longer; gas exchange ratio: lower vs placebo, *P* < 0.05 Secondary: inspiratory capacity: increased	Primary: ICPET variables; isokinetic muscle endurance Secondary: body composition
Hoogeveen *et al.*, 2021 [59]	Glycogen storage disease type IIIa (GSDIIIa) *n* = 6/6	Ketone ester + carbohydrate drink KE 395 mg/kg + CHO 30 g vs CHO drink ∼66 g	7 days; not reported	Primary: blood βHB: ANK induced in all subjects; median peak 2.6 mmol/l, *P* < 0.0001; glucose: lower in KE + CHO vs CHO, *P* < 0.0001; muscle energetics (^31^P-MRS): favorable quadriceps energetic state in KE + CHO vs CHO (overt myopathy subgroup); RER: stable on doubled workload in KE + CHO arm (overt myopathy subgroup) Secondary: insulin concentrations lower in KE + CHO arm; urinary βHB excretion increased in KE + CHO arm	Primary: postexercise Pi recovery kinetics (τPi) Secondary: blood lactate and FFA concentrations
Storgaard *et al.*, 2022 [60]	VLCAD or CPT II deficiency *n* = 5/4 (crossover)	Resveratrol 1000 mg/day	8 weeks; 92 ± 8% adherence to RSV, 94 ± 6% to placebo	–	Primary: heart rate at end of exercise; rate of palmitate oxidation Secondary: perceived exertion (Borg scale); fatigue (FSS); Bouchard diary
Some concerns					
Andersen *et al.*, 2008 [61]	McArdle disease *n* = 4/3 (crossover)	Carbohydrate-rich vs protein-rich diet CHO-rich: 65% CHO/15% protein/20% fat Protein-rich: 55% protein/30% CHO/15% fat	3 days each (1-week washout); not reported	Primary: exercise capacity (heart rate, Borg scale): lower on CHO-rich diet; *P* < 0.0005	Secondary: plasma glucose; serum lactate (higher in CHO group but not primary end point)
Bleeker *et al.*, 2020 [62]	VLCAD deficiency *n* = 2/3	Ketone ester + carbohydrate drink Ketone ester 395 mg/kg + dextrose 54 g	7 days; not reported	Primary: muscle energetics (^31^P-MRS, Pi/PCr): reduced ≥40% in KE arm vs CHO in overt myopathy subgroup; respiratory exchange ratio: stable on doubled workload	Primary: PCr recovery time constant Secondary: intramuscular fat oxidation
Gillingham *et al.*, 2019 [63]	LC-FAODs (TFP, LCHAD, CPT2, VLCAD deficiency) *n* = 7/6	High-protein diet (Hi PRO) Whey protein supplement + high protein diet (25% of daily caloric demand) vs high-carbohydrate diet (Hi CHO)	4 months; not reported	Primary: lean body mass: maintained in Hi PRO (Hi CHO group lost lean mass, *P* = 0.02)	Primary: muscle and licer lipid deposition Secondary: resting energy expenditure; TEE; fasting FFA, glucose, insulin
Glover *et al.*, 2010 [64]	Mitochondrial myopathy and cytopathies *n* = 15/15	Coenzyme Q10 (CoQ10) 600 mg twice daily	60 days; not reported	Primary: VO2/kg lean mass on ergometer (at 15 min): increased; *P* = 0.046; Secondary: post-exercise lactate: attenuated; gray matter choline compounds: decreased	Primary: BMD; lean body mass; forearm strength (NIRS); heart rate, RER, expired volume on ergometer; ADL, QoL Secondary: urinary markers of oxidative stress
Løkken *et al.*, 2021 [65]	Mitochondrial myopathy *n* = 11/11	Resveratrol 500 mg twice daily	16 weeks; 97% compliance for both the groups	–	Primary: exercise capacity (heart rate, cycle ergometer) Secondary: plasma lactate, pyruvate; Borg scale; QoL (SF-36); fatigue (FSS), indirect calorimetry measures, changes in peak Wmax
Ørngreen *et al.*, 2003 [25]	CPT II deficiency *n* = 4/4 (crossover)	High-carbohydrate diet 65% CHO/15% protein/20% fat vs high-fat diet: 60% fat/15% protein/25% CHO	3 days each; not reported	Primary: perceived exertion (Borg scale): lower on CHO diet; *P* < 0.005, exercise duration, heart rate	Secondary: plasma glycerol, FFA, alanine, lactate, glucose at rest and exercise
Scheffers *et al.*, 2023 [55]	Pompe disease (paediatric) *n* = 8/6	High-protein diet + exercise training Protein 2 g/kg/day + tailored exercise 45–60 min vs no exercise (control)	12 weeks; median training session attendance 94.4%.	Primary: peak VO_2_ (maximal CPET): improved within group; *P* = 0.039 Secondary: muscle strength (total sum score), core stability, parent-reported QoL, child-reported fear of exercise	Primary: peak VO_2_ vs control; 6MWT; heart rate on submaximal CPET Secondary: QMFT, STST, body composition; serum CK
Tarnopolsky *et al.*, 1997 [66]	Mitochondrial cytopathies *n* = 7/7	Creatine monohydrate 5 g twice daily × 2 weeks, then 2 g/day × 1 week	3 weeks; not reported	Primary: handgrip strength, *P* < 0.05; NIDFT, *P* < 0.01; postexercise lactate, *P* < 0.05	Primary: ADL score, 2MWT, body composition, cycle ergometry (VO_2_, heart rate, RER)
Vorgerd *et al.*, 2000 [67]	McArdle disease *n* = 4/5	Creatine monohydrate Loading: 150 mg/kg/day × 1 week; Maintenance: 60 mg/kg/day	5 weeks; not reported	Primary: PCr depletion during aerobic exercise: increased; *P* = 0.006 Secondary: serum creatine; EMG median frequency decrease: larger with creatine	Primary: maximum workload; maximal exercise duration Secondary: fatigue (FSS)
Vorgerd *et al.*, 2002 [68]	McArdle disease *n* = 10/9	High-dose vs low-dose creatine High dose: 150 mg/kg/day Low dose (comparator): 60 mg/kg/day	5 weeks; two dropouts due to noncompliance	Primary: high-dose creatine worsened exercise-induced muscle pain (*P* = 0.02) and limited daily activities (*P* = 0.005) Secondary: serum creatine, EMG smaller increase in amplitude with creatine	Secondary: muscle bioenergetics (MRS); CPK
High risk of bias					
Klopstock *et al.*, 2000 [56]	Mitochondrial myopathy/CPEO *n* = 16/16	Creatine monohydrate 5 g orally, four times daily	8 weeks; not reported	–	Primary: muscle strength (MRC); Hammersmith motor score; maximal voluntary torques; NSS; function time test Secondary: resting and post-exercise lactate; ataxia score
Løkken *et al.*, 2020 [24]	McArdle disease *n* = 10 (no control)	Modified ketogenic diet (three regimes) Diet 1: 65% fat/15% protein/20% CHO Diet 2: 75% fat/15% protein/10% CHO Diet 3: 80% fat/15% protein/5% CHO	3 weeks each; two patients on diet 1 withdrawn due to compliance issues, diet 2 had highest acceptability	Primary: exercise capacity (heart rate, Borg scale): lower post-diet vs baseline across all regimes Secondary: fatty acid oxidation: increased; CHO oxidation: decreased; blood ketones highest on diet 3	No control group; between-diet statistical comparison not performed
Løkken *et al.*, 2022 [69]	McArdle disease *n* = 8/4 (healthy controls)	Ketone ester (KE) drink KE drink 395 mg/kg	2 days; not reported	Secondary: plasma HOB, AcAc and relative KB oxidation	Primary: exercise capacity (heart rate, ergometer) Secondary: perceived exertion (Borg scale)
Løkken *et al.*, 2023 [70]	McArdle disease *n* = 11/9 (crossover)	Modified ketogenic diet (mKD) 80% fat/15% protein/5% CHO vs placebo diet (PD): 50% fat/15% protein/35% CHO	3 weeks each; mean compliance 96.6% for mKD, 96.9% for PD	Secondary: plasma HOB, AcAc; QoL (SF-36); fatigue (FSS); patient-reported symptoms	Primary: exercise capacity (heart rate); perceived exertion (Borg scale)
Mancuso *et al.*, 2010 [71]	Mitochondrial myopathy *n* = 27/42	Whey-based cysteine donor 10 g/day	1 month; two participants excluded due to non-compliance	Secondary: antioxidant markers (FRAP, AOPP at rest, AOPP post-exercise)	Primary: muscle strength (MRC); QoL (SF-36)
Sechi *et al.*, 2020 [54]	Late-onset Pompe disease (LOPD) *n* = 13/13	High-protein diet + exercise training 25–30% protein/30–35% CHO/35–40% fat + aerobic exercise vs no exercise (control)	26 weeks; not reported	Primary: peak VO_2_ (cycle ergometer): exercise alone (*P* = 0.05) and exercise + diet (*P* = 0.009); QoL (SF-36): general health and vitality exercise + diet (*P* = 0.03) Secondary: serum CK, LDH: reduced after exercise + diet vs exercise alone; FEV1%: improved after exercise + diet only	Primary: 6MWT Secondary: vital capacity, AST, ALT, muscle strength, Walton scale
Critical illness myopathy					
Low risk of bias					
Viana *et al.*, 2021 [72]	Critically ill, mechanically ventilated patients *n* = 30 (15/15)	HMB (β-hydroxy-β-methylbutyrate) 3 g/day	30 days; 100% in survivors	Secondary: net protein breakdown: reduced; phase angle (BIA): increased; global health (SF-12): improved	Primary: quadriceps muscle area loss (SMA, ultrasound): overall loss but no between-group difference
Some concerns					
Supinski *et al.*, 2021 [73]	Mechanically ventilated adult patients *n* = 73 (EPA = 17, HMB = 18, HMB + EPA = 18, placebo = 20)	Arm 1: EPA 2 g/day Arm 2: HMB 3 g/day Arm 3: HMB + EPA 3 g + 2 g/day	11 days; good compliance (average 17–19 doses received)	–	Primary: diaphragm strength (PdiTw); quadriceps strength (QuadTw); diaphragm and quadriceps thickness (US)
High risk of bias					
Verceles *et al.*, 2023 [74]	Critically ill, mechanically ventilated patients *n* = 39 (16/23)	High-protein whey supplement + NMES + physical therapy Whey protein 1.75 g/kg/day; 20 NMES sessions over 14 days (vs standard care)	14 days; average 10.1/20 NMES sessions received	Primary: attenuation of muscle volume loss (CT, lower extremity): thigh, *P* = 0.03; lower leg, *P* = 0.05 Secondary: nitrogen balance: positive on day 9	Primary: thigh cross-sectional area (combined mean) Secondary: delirium; ICU length of stay; days on mechanical ventilation

Abbreviations: AcAc, aceto-acetate; ADL, activities of daily living; ALT, alanine aminotransferase; AOPP, advanced oxidation protein products; AST, aspartate aminotransferase; βHB, beta-hydroxybutyrate; BIA, bioelectrical impedance analysis; BMD, bone mineral density; CHO, carbohydrate; CK, creatine kinase; CoQ10, coenzyme Q10; CPEO, chronic progressive external ophthalmoplegia; CPET, cardiopulmonary exercise test; CPK, creatine phosphokinase; CPT II, carnitine palmitoyltransferase II; CWR, constant work rate exercise test; EMG, electromyogram; EPA, eicosapentaenoic acid; FAO, fatty acid oxidation; FEV1, forced expiratory volume in 1 s; FFA, free fatty acids; FRAP, ferric reducing antioxidant power; FSS, fatigue severity scale; GSDIIIa, glycogen storage disease type IIIa; HMB, β-hydroxy β-methylbutyrate; HOB, beta-hydroxy-butyrate; ICPET, Incremental Cardiopulmonary Exercise Test; ICU, intensive care unit; KB, ketone bodies; KE, ketone ester; LC-FAOD, long-chain fatty acid oxidation disorder; LCHAD, long-chain hydroxy-acyl CoA dehydrogenase; LDH, lactate dehydrogenase; LOPD, late-onset pompe disease; MELAS, mitochondrial myopathy encephalopathy lactic acidosis and stroke-like episodes; MERRF, myoclonic epilepsy with ragged red fibres; mKD, modified ketogenic diet; MRC, Medical Research Council scale; MRS, magnetic resonance spectroscopy; NIDFT, non-ischaemic dorsiflexion torque; NIRS, near-infrared spectroscopy; NMES, neuromuscular electrical stimulation; NSS, neuromuscular symptom score; PCr, phosphocreatine; PD, placebo diet; Pi, inorganic phosphate; QMFT, quick motor function test; RER, respiratory exchange ratio; RSV, resveratrol; SF-12, short form-12 health survey; SF-36, short form-36 health survey; SMA, skeletal muscle area; STST, supine to stand test; τPi, time constant of inorganic phosphate recovery; TEE, total energy expenditure; TFP, trifunctional protein deficiency; VLCAD, very long-chain acyl-CoA dehydrogenase; VO_2_, oxygen uptake; Wmax, maximal workload; 2MWT, 2-min walk test; 6MWT, 6-min walk test; ^31^P-MRS, phosphorus-31 magnetic resonance spectroscopy.

### Critical illness myopathy

HMB-enriched whey protein with neuromuscular electrical stimulation and physical therapy significantly attenuated lower extremity muscle volume loss in one study [74], while two other trials evaluating HMB alone or in combination with omega-3 fatty acids showed mixed results [72, 73] (Table 3).

### Adverse events

Most nutritional interventions were well tolerated with no serious adverse events reported. Creatine supplementation demonstrated acceptable safety across multiple conditions including muscular dystrophies, inflammatory myopathies and most metabolic myopathies, with no adverse effects observed in the majority of trials. However, in McArdle disease, high-dose creatine (150 mg/kg/day) worsened exercise-induced pain and activity limitations compared with lower doses [68]. The highest rates of adverse events occurred with penicillamine and BCAA supplementation in PM/DM, though the latter was confounded by concomitant high-dose glucocorticoid use. Details of adverse events are reported in Table 4.

**Table 4:** Summary of adverse events reported across studies.

Disease	Study	Intervention	Adverse events
Inflammatory myopathies			
JDM	Dover 2021 [29]	Creatine (150 mg/kg/day)	Transient reversible dehydration with elevated serum creatinine (*n* = 1)
PM/DM	Kimura 2022 [27]	BCAA	Heart failure (*n* = 1), myocarditis, depression, suicide attempt (*n* = 1), pneumocystis pneumonia, urolithiasis, interstitial lung disease, anorexia, odontectomy. Most attributed to high-dose glucocorticoids; IRB could not exclude potential BCAA-glucocorticoid-antidepressant interactions
Muscular dystrophies			
DMD	Davidson 2021 [43]	Multicomponent nutritional supplement	Rash (*n* = 3), elevated blood pressure (*n* = 1), resting tachycardia (*n* = 1), mild tachycardia (*n* = 2), weight gain (*n* = 2), respiratory infection (*n* = 1), GI illness (*n* = 1), ear/throat infection (*n* = 1)
	Fenichel 1988 [35]	Penicillamine (125–500 mg twice daily) + vitamin E (600–1200 mg twice daily)	Nausea/vomiting (*n* = 8), anorexia (*n* = 8), rash (*n* = 14), taste disturbance (*n* = 2), fever (*n* = 3), mouth sores (*n* = 7). Eight patients withdrawn due to adverse reactions
	Hafner 2019 [48]	L-citrulline (2.5 g three times daily) + metformin (250 mg three times daily)	Mild transient gastrointestinal symptoms (*n* = 4)
	Mendell 1984 [37]	L-leucine (0.2 g/kg/day)	Gastrointestinal side effects: decreased appetite, anorexia, nausea
	Mok 2009 [38]	Glutamine (0.5 g/kg/day)	Gastroenteritis (*n* = 1), urticaria (*n* = 1), nervousness (*n* = 1)
Myotonic dystrophy	Orndahl 1984 [49]	Selenium (0.4–1.6 mg/day) + vitamin E (200–800 mg/day)	Slight diarrhoea in a few patients
Distal myopathy			
GNE myopathy	Mori-Yoshimura 2023 [53]	Aceneuramic acid 1500 mg/day	Dry eyes, GERD, diarrhoea, fevers, nasopharyngitis
	Park 2023 [52]	6′-Sialyllactose (6SL) 6 g/day	Constipation (*n* = 1), intermittent and transient headache (*n* = 1)
Metabolic & mitochondrial myopathies			
McArdle disease	Vogerd 2002 [68]	Creatine 150 mg/kg/day	Increased exercise induced pain and activity limitations at high doses
	Lokken 2020 [24]	Modified ketogenic diet	Mild fatigue and headache (*n* = 3), mild nausea (*n* = 2)
	Lokken 2023 [70]	Modified Ketogenic diet	Mild transient headache, fatigue, nausea, diarrhea/constipation
LC-FAODs	Gillingham 2019 [63]	High protein diet (25% daily calories from whey protein)	Subtle vision loss (*n* = 1)
Mitochondrial myopathy	Klopstock 2000 [56]	Creatine 20 g/day	Muscle cramps (*n* = 2)
	Lokken 2021 [65]	Resveratrol 1000 mg/day	Joint stiffness (*n* = 3), dizziness (*n* = 2), Mild GI symptoms like diarrhoea
CPT II	Storgaard 2022 [60]	Resveratrol 1000 mg/day	Headache (*n* = 2), sleeping of legs (*n* = 1), self-resolving bilateral dorsal hand eczema (*n* = 1)

Abbreviations: BCAA, branched chain amino acids; DMD, Duchenne muscular dystrophy; FSHD, facioscapulohumeral dystrophy; FFM, fat-free mass; GI, gastrointestinal; HMB, β-hydroxy-β-methylbutyrate; IRB, institutional review board; LC-FAODs, long-chain fatty acid oxidation disorders; VLCAD, very long-chain acyl-CoA dehydrogenase; CPT II, carnitine palmitoyltransferase II, GERD, gastroesophageal reflux disease.

## Discussion

This systematic review of 51 randomized controlled studies reveals considerable heterogeneity in both the quality and outcomes of nutritional interventions across muscle diseases. The evidence base is substantially larger for non-inflammatory myopathies, particularly muscular dystrophies and metabolic myopathies, compared with inflammatory myopathies. This disparity likely reflects multiple factors beyond disease prevalence, notably the absence of curative treatments for non- inflammatory myopathies [75]. The complexity of immune dysregulation [76] and treatment-related complications in inflammatory myopathies [11] makes nutritional interventions more difficult to study and potentially less mechanistically straightforward than in conditions with defined metabolic defects. The dominance of corticosteroids in myositis treatment—which themselves induce muscle catabolism [77]—should logically drive research into nutritional strategies addressing these complications, yet this area remains underdeveloped.

Creatine supplementation was the most frequently studied single intervention across disease subtypes, demonstrating variable efficacy. This effect is biologically plausible given creatine’s well-established role in muscle energy metabolism through phosphocreatine regeneration, which facilitates rapid ATP synthesis during muscle contraction [78]. A 2013 Cochrane review of creatine for muscle disorders [79] found moderate-quality evidence for modest short-term increases in muscle strength in muscular dystrophies but no benefit in most metabolic myopathies, concluding that evidence was insufficient to recommend routine creatine use. Our review, including additional recent trials in JDM [26, 29], largely confirms these findings. In adult inflammatory myopathy, a single trial [28] combining high-dose creatine with a home exercise program showed a 13% improvement in aggregate functional performance time (AFPT) alongside gains in strength and metabolic outcomes. The AFPT measures the time taken to complete a series of functional tasks including rising from the floor, climbing and descending stairs, and a 50-foot walk [28]; a reduction represents a gain in the ability to perform daily activities, beyond the threshold typically considered important to patients with inflammatory myopathy. However, as exercise was mandated in both groups, attributing benefits specifically to creatine vs enhanced exercise tolerance remains problematic. In JDM, creatine improved metabolic parameters (Pi/PCr ratio, pH recovery) without translating to functional gains [29], possibly reflecting outcome measure insensitivity, or suggesting that biochemical benefit alone may be insufficient to change the patient experience. Standard strength tests like MMT may have ceiling effects in milder disease, failing to capture endurance or fatigability changes detected by metabolic measures [80]. A related but distinct limitation is seen in adult inflammatory myopathy studies, where functional index scores improved significantly despite unchanged MMT scores [27], suggesting that dynamic, repetitive function assessments may better capture treatment effects than maximal strength testing. These observations highlight the inadequacy of relying on a single outcome domain and suggest that future trials should incorporate both functional and patient-reported measures as benchmarks for clinical significance. Across muscular dystrophies, creatine showed modest improvements in body composition and isolated strength measures in DMD but no functional benefits. Alarmingly, in McArdle disease, creatine paradoxically worsened symptoms at doses >150 mg/kg/day [68], highlighting disease-specific metabolic considerations that preclude universal application.

Ketogenic diets in McArdle disease exemplify how mechanistically targeted nutrition can address specific metabolic defects, with studies showing improved exercise capacity Although this carries substantial day-to-day significance with respect to physical activity and employment for patients with McArdle’s disease, the practical challenge of maintaining highly restrictive diets, combined with risks of dyslipidaemia and micronutrient deficiencies [81] raises questions about real-world applicability beyond short-term supervised trials. In critical illness myopathy, HMB-enriched whey protein combined with physical interventions showed positive trends for muscle mass preservation. Given that current guidelines recommend protein intake of 1.2–2.0 g/kg/day for critically ill patients [82–84], the question is not whether high-protein interventions are effective, but whether specific formulations or timing strategies confer advantages over standard high-protein feeding. The biological rationale is sound [85], but demonstrating incremental benefits requires larger studies extending into post-ICU recovery.

The four studies examining sialic acid-based therapies for GNE myopathy demonstrate targeted nutritional supplementation can yield functional benefits in rare muscle diseases. GNE myopathy is an autosomal recessive distal myopathy caused by mutations in the GNE gene, resulting in sialic acid deficiency and hyposialylation of muscle glycoproteins; sialic acid supplementation aims to bypass this enzymatic defect and slow progressive muscle degeneration [51]. Across three RCTs, aceneuramic acid 6 g/day over 48 weeks consistently maintained upper extremity composite (UEC) strength vs placebo decline, while functional activity scales and 6-min walk test did not improve significantly [50, 51, 53]. In contrast, 6′-sialyllactose 6 g/day improved lower extremity composite (LEC) strength across multiple muscle groups and attenuated muscle fat fraction increase on MRI [52], suggesting a complementary but distinct pattern of effect. Together, these findings formed the evidence base that led to regulatory approval in Japan in March 2024 [86], representing a significant advance for a previously untreatable condition, though questions remain regarding long-term efficacy and whether similar supplementation strategies might benefit other distal myopathies.

The variable and largely inconsistent results with supplements such as coenzyme Q10, resveratrol and L-carnitine across different muscle disease populations highlight another pattern: interventions that lack clear mechanistic rationale for specific genetic or metabolic defects generally show weak or contradictory effects. This variability may reflect genuine differences in treatment response across genetic subtypes, but more likely stems from methodological limitations.

Across the included trials, positive outcomes tended to emerge when nutritional interventions were combined with structured physical rehabilitation rather than delivered in isolation: creatine alongside a home exercise programme improved functional performance in adult IIM, though both arms received the exercise co-intervention [28]; high-protein diet combined with aerobic training improved peak VO_2_ and quality of life in both adult and paediatric Pompe disease [54, 55] and protein supplementation with training in FSHD conferred no additional benefit beyond training alone [61]. Exercise is now recognized as an important therapeutic modality across the inflammatory myopathies, with evidence supporting its efficacy in reducing disease activity and improving functional capacity [87]. Future trials in this field should incorporate standardized exercise co-interventions and report them transparently, to allow isolation of nutritional effects within a multimodal treatment framework. The methodological quality assessment revealing only 31% of studies at low risk of bias, with 49% raising some concerns and 20% at high risk, indicates that much of this evidence base rests on a weak foundation. The most problematic deficiencies—inadequate sample sizes, poor or unclear randomization, lack of blinding and selective outcome reporting—are particularly concerning for nutritional interventions where placebo effects and reporting bias can substantially inflate apparent benefits, especially for subjective outcomes. Dietary interventions present inherent methodological challenges, including difficulties controlling for baseline dietary intake, ensuring compliance with prescribed dietary patterns [88] and interpreting multicomponent formulations where individual contributions remain unclear. Null findings in this evidence base should not be conflated with evidence of no effect, as underpowered trials lack the sensitivity to detect clinically meaningful signals. The absence of blinding is particularly consequential for subjective outcomes such as fatigue and quality of life, where placebo effects are well recognized, and limits confidence in positive findings in these domains.

A significant challenge to evidence synthesis is the heterogeneity in outcome measures across studies. While diverse assessment tools reflect the heterogeneous nature of these conditions and their specific functional domains, this variation along with the lack of standardized effect sizes made formal meta-analyses impossible, even within more homogeneous subgroups such as IIM or DMD. Additionally, the predominance of short study durations, typically weeks to months, poses a fundamental problem: slowly progressive conditions require years of follow-up to demonstrate disease modification, yet few studies extend beyond the intervention period to assess durability of effects or long-term safety, limiting our ability to draw firm conclusions about relative efficacy. Given the rarity of these conditions, innovative trial designs such as basket trials could prove valuable for this patient population.

Several clinical implications emerge from this evidence (Table 5). For patients with inflammatory myopathies, creatine supplementation combined with exercise warrants consideration as low-cost, low-risk adjunctive therapy, considering the favourable safety profile and modest functional benefits, although evidence is limited to one trial in adults and two in children. Conversely, BCAA supplementation cannot be recommended yet given lack of benefit and potential adverse interactions with high-dose glucocorticoids [27]. For conditions with defined enzymatic defects, mechanistically targeted approaches such as ketogenic diets in McArdle disease or aceneuramic acid in GNE myopathy justify individualized trials within a multidisciplinary framework.

**Table 5:** Clinical summary table.

Intervention & dose	Disease	Summary of evidence
Supported by current evidence		
Creatine + exercise Loading 20 g/day → 3 g/day maintenance	PM/DM (adult)	Improved functional performance and muscle energetics in one RCT
Aceneuramic acid 6 g/day	GNE myopathy	Consistent benefit on upper extremity strength across three RCTs; regulatory approval in Japan
Carbohydrate-rich diet (65% CHO)	McArdle disease; CPT II deficiency	Reduced exercise-related symptoms; mechanistically targeted; supported by small RCTs
Modified ketogenic diet (80% fat/5% CHO)	McArdle disease	Improved quality of life and fatigue in one RCT; practical adherence challenges
High-protein diet + exercise (∼25–30% protein)	Pompe disease	Improved aerobic capacity and quality of life across two RCTs (adult and paediatric)
Insufficient evidence		
Creatine 0.1–0.15 g/kg/day	JDM	Metabolic benefit on MRS; no functional or strength gains in two RCTs
BCAA (TK-98) 4.15 g × 6 packs/day	PM/DM (adult)	Secondary functional gains only; primary strength outcome not met in one RCT
Creatine 2–10 g/day	Duchenne muscular dystrophy	Body composition benefit in 3/4 RCTs; no consistent functional improvement
Omega-3 LC-PUFAs 2.9 g/day (EPA + DHA)	Duchenne muscular dystrophy	Anti-inflammatory biomarker benefit in one RCT; no functional endpoints assessed
Antioxidants (Vit C 500 mg + Vit E 400 mg + Zn + Se)	Facioscapulohumeral dystrophy (FSHD)	Quadriceps strength improved in two RCTs; no functional (walking) benefit
6′-Sialyllactose 6 g/day	GNE myopathy	Lower extremity strength and MRI benefit in one small pilot RCT; requires replication
Coenzyme Q10 160–1200 mg/day	Mitochondrial myopathy	Conflicting results across two RCTs; heterogeneous patient populations
HMB 3 g/day ± omega-3	Critical illness myopathy	Protein catabolism benefit in one RCT; no muscle volume or strength benefit in two RCTs
Risk considerations		
High-dose creatine 150 mg/kg/day	McArdle disease	Worsened exercise-induced pain and limited daily activities in one RCT
BCAA + high-dose glucocorticoids	PM/DM	Serious adverse events; potential drug–nutrient interaction not excludable
Penicillamine + vitamin E	Duchenne muscular dystrophy	Significant adverse events with no functional benefit in one RCT

Abbreviations: BCAA, branched chain amino acids; CHO, carbohydrate; CPT II, carnitine palmitoyltransferase II; DHA, docosahexaenoic acid; EPA, eicosapentaenoic acid; FSHD, facioscapulohumeral muscular dystrophy; GNE, glucosamine (UDP-N-acetyl)-2-epimerase/N-acetylmannosamine kinase; HMB, β-hydroxy-β-methylbutyrate; LC-PUFAs, long-chain polyunsaturated fatty acids; MRS, magnetic resonance spectroscopy; RCT, randomized controlled trial; Se, selenium; Vit C, vitamin C; Vit E, vitamin E; Zn, zinc.

In 2022, ACR published guidelines for the management of RA with exercise, rehabilitation, diet and additional integrative interventions, emphasizing a ‘food-first’ approach and encouraging an anti-inflammatory, well-balanced Mediterranean diet to combat the chronic inflammatory disease state [89]. The paucity of trials in inflammatory myopathies, combined with mechanistic plausibility from related conditions, identifies high-priority interventions for future myositis trials: creatine plus exercise, high-protein diets to counter steroid effects and anti-inflammatory dietary patterns (Mediterranean diet) given success in RA [89].

Key priorities for future research include: adequately powered, multicentre RCTs using rigorous methodology and international collaborative networks given disease rarity; development of standardized core outcome sets prioritizing patient-centred measures to enable meta-analysis; increased research attention to inflammatory myopathies, particularly examining anti-inflammatory dietary patterns and nutritional strategies to counteract corticosteroid-induced complications; long-term studies assessing effects on disease progression and functional outcomes over years rather than months and pragmatic trials in real-world settings to evaluate effectiveness and feasibility in actual clinical practice.

## Conclusion

While our study compiles existing data and brings forward key outcomes in the nutritional impact on muscle diseases, the current evidence base remains limited particularly regarding inflammatory myopathies. The strongest support emerged for mechanistically targeted approaches such as creatine supplementation and disease-specific interventions like sialic acid in GNE myopathy, although heterogeneity in study design and outcome measures limits definitive conclusions. Standardized outcome measures, international collaboration and long-term observational research are essential to establish whether nutritional interventions can meaningfully impact disease progression and quality of life.

## Supplementary Material

keag378_Supplementary_Data

## Data Availability

No new data were generated or analysed in support of this research.

## Disclaimer

The findings from this systematic literature review were previously presented as an abstract at the American College of Rheumatology (ACR) Convergence 2025.

Vedantam D, Talreja T, Bandarupalli P, Salis S, Lupeika D, Maclean A, Cheng K, Liewluck T, Nagendra L, Gupta L. Systematic review: nutritional interventions in muscle diseases [abstract]. Arthritis Rheumatol 2025;77(suppl 9). https://acrabstracts.org/abstract/systematic-review-nutritional-interventions-in-muscle-diseases/ (23 November 2025, date last accessed).
