# Supplementary material for: Nutritional interventions and dietary supplements in muscle diseases: a systematic review
Source: Rheumatology (Oxford). 2026 Jul 21;65(8):keag378. doi: 10.1093/rheumatology/keag378 (PMC13600409; doi:10.1093/rheumatology/keag378)
Supplement: Supplementary file 1 [file keag378_supplementary_data.docx]

**Supplementary Data S1: Detailed inclusion and exclusion criteria**

**PARTICIPANTS (P)**

People with a diagnosis of muscle conditions at any age, including:

**Inflammatory Myopathies:**

- Dermatomyositis (adult and juvenile), DM, JDM
- Polymyositis (adult and juvenile), PM, JPM
- Immune-mediated necrotizing myopathy (IMNM), NM, NAM, anti-HGMCR NM, anti-SRP IMNM (adult and juvenile)
- Antisynthetase syndrome ASyS (adult and juvenile)
- Clinically amyopathic dermatomyositis CADM/JCADM (adult and juvenile)
- Overlap myositis (adult and juvenile)
- Inclusion body myositis (IBM)
- Amyopathic dermatomyositis (Adult and Juvenile)
- Connective tissue disease-associated myositis
- Scleromyositis
- Cancer-associated myositis (CAM)

**Non-inflammatory Muscle Conditions:-**

- Muscular dystrophies (Facioscapulohumeral dystrophy, Limb-girdle muscular dystrophy, Emery-Dreifuss muscular dystrophy, oculopharyngeal muscular dystrophy, and dystrophinopathy)
- Myotonic dystrophies (Type 1 and 2)
- Non-dystrophic myotonia (myotonia congenita and paramyotonia congenita)
- Congenital myopathies (late onset variants)
- Congenital myotonic dystrophy
- Metabolic myopathies- Lipid myopathies (carnitine palmitoyltransferase II deficiency, very-long-chain-acyl-CoA dehydrogenase deficiency, trifunctional protein deficiency; Glycogen storage disease myopathy  (McArdle's disease, Pompes, Cori’s)
- Mitochondrial myopathies
- Myofibrillary myopathy, GNE myopathy, and Multisystem proteinopathy 1-4
- Critical illness myopathy
- Distal myopathy

**INTERVENTIONS (I)**

Dietary interventions including:

**Macronutrients**

**Proteins**

- High/low protein diets
- Casein-free diet
- Protein supplements:
  - Whey
  - Pea
  - Soy
  - Casein
- Amino acids:
  - Leucine
  - BCAAs (Branched-Chain Amino Acids)
  - Glutamine
  - HMB (β-Hydroxy β-Methylbutyrate)
- Creatine

**Carbohydrates**

- Carbohydrate-restricted diet
- High-carbohydrate diets
- FODMAP
- Lactose-free diet
- High fructose diet and high sugar content food

**Fats**

- High saturated fat and trans-fat diet
- Low-fat diet
- Ketogenic/high-fat diet
- Omega-6 fatty acids
- Omega-3 fatty acids
- Fish oils

**Micronutrients**

- **Vitamins**:
  - Vitamin A
  - Vitamin C
  - Vitamin D
  - Vitamin E
  - Vitamin B9 / Folic Acid
  - Vitamin B12
  - Vitamin B2
- **Minerals**:
  - Calcium
  - Zinc
  - Magnesium
  - Potassium
  - Selenium
- **Other Micronutrients**:
  - Biotin

**Supplements / Other Nutritional Compounds**

**Anti-inflammatory & Antioxidant Supplements**

- Turmeric / Curcumin
- Ginger
- Antioxidants:
  - Coenzyme Q10
  - Green tea / Black tea
  - Dark chocolate
  - Herbs and spices
  - Berries
  - Citrus fruits
  - Red wine
  - Legumes
  - Purple grapes
  - Nuts and seeds
  - Isoflavones

**Probiotic-Related**

- Prebiotics
- Probiotics
- Synbiotics
- Postbiotics

**Polyphenol-rich Foods & Extracts**

- Polyphenol-rich foods, juices, and concentrates

**Miscellaneous Supplements**

- Red yeast rice
- Oyster mushrooms
- Spirulina

**Dietary Patterns / Other Diets**

**Plant-based & Regional Diets**

- Plant-based / High-fiber diets
- Mediterranean diet
- Nordic diet
- Gluten-free diet
- Anti-inflammatory diet
- Calorie-restricted diets
- Dietary Approach to Stop Hypertension (DASH) diet
- Mediterranean DASH Intervention for Neurodegenerative Delay (MIND) diet
- Immunonutrition diet
- Lacto-ovo-vegetarian diet
- Modified Atkins diet

**Alternative/Traditional Medicine Diets**

- Paleo diet
- Ayurvedic diet
- Traditional Chinese medicine diet
- Raw food diet
- Alkaline diet

**Berry-based Diets for Muscle Health**

- Raspberries
- Blueberries
- Aronia
- Elderberry
- Mixed compound berry-based diet

**Processed or Unbalanced Diets**

- Processed food-based diets
- Ultraprocessed food-based diets
- Unbalanced vegetarian/vegan diets
- Pescatarian diets

**Sodium/Caloric Restriction**

- Sodium-restricted diet
- Calorie-restricted diets

**Fasting Regimens**

- Intermittent fasting:
  - Alternate day
  - 5:2 regimen
  - Time-restricted feeding
  - B2 regimen
  - Weekly 1-day fasting
  - Intermittent VLCD (Very Low-Calorie Diet) therapy

**Lifestyle Factors**

- Caffeine intake
- Alcohol
- Smoking

**COMPARISONS (C)**

- Standard care/usual diet
- Pharmacological interventions
- Different dietary interventions
- Different regimens of the same intervention
- Placebo
- Combination therapies

**OUTCOMES (O)-**

**Disease Activity Measures:**

- Muscle strength - Manual Muscle Testing (MMT8, MMT26), Fixed dynamometry, Isokinetic dynamometry, Specific dynamometry, composite Maximum Voluntary Isometric Contraction Test (MVICT) score, DEXA lean body mass scan, mean grip strength, Quantitative Muscle Testing (QMT), 1Repetition Maximum, manual muscle testing)
- Physician/patient global assessments -Total Improvement score (TIS), Physician global impression of change (or physician assessment of change), Patient/parent global impression of change (or patient assessment of change, Physician global disease activity, Patient/parent global disease activity, Extra-muscular global activity (10 cm VAS), Myositis Disease Activity Assessment Tool (MDAAT), Disease Activity Score (DAS)
- Physical function -Functional Index-2, Barthel Index, Health Assessment Questionnaire (HAQ/CHAQ), Myositis Activity Profile (MAP), Patient Reported Outcome Measurement Information System (PROMIS), 6 minute and 2 minute walk test, Timed 10 m walk/run test, 30 sec 1kg arm rise test, arm lift test, Childhood Myositis Assessment Scale (CMAS), Inclusion Body Myositis Functional Rating Scale (IBMFRS)
- Muscle enzyme levels (CK, AST, ALT, LDH, aldolase, troponins)
- Standardized assessment tools (IMACS, PRINTO core sets, MDAAT, DAS, CMAS, CHAQ/HAQ)
- Muscle biopsy features of IIM such as  biopsy grading scales

Organ-specific assessments:

- Pulmonary- PFT, HRCT, Bronchoalveolar lavage, Echocardiogram for PAH, Bronchoscopy/surgical biopsy, Myositis Intention to Treat Index (MITAX), Myositis Disease Activity Assessment Visual Analogue Scales (MYOACT), King's Brief Interstitial Lung Disease questionnaire (K-BILD),  6MWT: 6‐minute walk test, SGRQ: St George's Respiratory Questionnaire, Dyspnoea score
- Cardiac- Cardiac function testing- Cardiopulmonary exercise testing (CPET), Major cardiovascular event (MACE), ECG, echocardiogram, troponins, cardiac MRI, Skin, muscle, Cutaneous/ Organ Response Evaluation (SCORE, mSCORE), [Carotid Intima-Media Thickness (CIMT)](https://www.google.com/search?client=safari&cs=0&sca_esv=17771aff06750f59&sxsrf=AHTn8zoLWZB_98NGGsZKZmlnfCyzuwGBkQ%3A1744098292121&q=Carotid+Intima-Media+Thickness+%28CIMT%29&sa=X&ved=2ahUKEwi3tOS4-MeMAxVmle4BHQ_oCTMQxccNegQIHhAB&mstk=AUtExfAUgyX7MPLB2948t67ajXtDNCzAVUqB6FLrfYfh6X950Kc_YUcHawdlmLb_BUhlZisBhCBzKH-673XMdDhEyOpWAm7cah01qMwv6jHaWIkAol3ijsPz7sC5WfdEJi9j_oVD76PeccganSvxGyUddDYmeAOmPDyjHdZJA_xdRbAVn6o6weL6jJM0WgumbZ9MIPaZtLZxJFbCj-AofwUaNSd_QieKCBwQHsXvSj3kUobr8qyBj1o5rBOFAen0he0bSw_FmbnJeTcQ7jSEYvfmBQWr&csui=3) and Plaque Detection, [Ankle-Brachial Index (ABI)](https://www.google.com/search?client=safari&cs=0&sca_esv=17771aff06750f59&sxsrf=AHTn8zoLWZB_98NGGsZKZmlnfCyzuwGBkQ%3A1744098292121&q=Ankle-Brachial+Index+%28ABI%29&sa=X&ved=2ahUKEwi3tOS4-MeMAxVmle4BHQ_oCTMQxccNegQIJxAB&mstk=AUtExfAUgyX7MPLB2948t67ajXtDNCzAVUqB6FLrfYfh6X950Kc_YUcHawdlmLb_BUhlZisBhCBzKH-673XMdDhEyOpWAm7cah01qMwv6jHaWIkAol3ijsPz7sC5WfdEJi9j_oVD76PeccganSvxGyUddDYmeAOmPDyjHdZJA_xdRbAVn6o6weL6jJM0WgumbZ9MIPaZtLZxJFbCj-AofwUaNSd_QieKCBwQHsXvSj3kUobr8qyBj1o5rBOFAen0he0bSw_FmbnJeTcQ7jSEYvfmBQWr&csui=3) and [Carotid-Femoral Pulse Wave Velocity (cf-PWV)](https://www.google.com/search?client=safari&cs=0&sca_esv=17771aff06750f59&sxsrf=AHTn8zoLWZB_98NGGsZKZmlnfCyzuwGBkQ%3A1744098292121&q=Carotid-Femoral+Pulse+Wave+Velocity+%28cf-PWV%29&sa=X&ved=2ahUKEwi3tOS4-MeMAxVmle4BHQ_oCTMQxccNegQIJxAC&mstk=AUtExfAUgyX7MPLB2948t67ajXtDNCzAVUqB6FLrfYfh6X950Kc_YUcHawdlmLb_BUhlZisBhCBzKH-673XMdDhEyOpWAm7cah01qMwv6jHaWIkAol3ijsPz7sC5WfdEJi9j_oVD76PeccganSvxGyUddDYmeAOmPDyjHdZJA_xdRbAVn6o6weL6jJM0WgumbZ9MIPaZtLZxJFbCj-AofwUaNSd_QieKCBwQHsXvSj3kUobr8qyBj1o5rBOFAen0he0bSw_FmbnJeTcQ7jSEYvfmBQWr&csui=3)
- Cutaneous- Cutaneous assessment tools- CDASI (Cutaneous dermatomyositis activity and severity index), DLQI (Dermatology Life Quality Index), CAT (Cutaneous Assessment Tool) and DSSI (Dermatomyositis Skin Severity Index), Disease Activity Score (DAS-skin), Dermatomyositis Investigator global assessment (DM-IGA) skin measure, Skindex-29 and Skindex-16, Extramuscular Global Assessment (EMGA), Trunk Impairment Scale (TIS).
- Gastrointestinal-   Swallowing assessments (videofluoroscopy, ultrasound, MRI, barium swallow studies, swallow questionnaires), MDAAT score for Gastroparesis and intestinal dysmotility assessment, gastric emptying study, wireless motility capsule, and colonic transit studies. Assessment of gastrointestinal vasculopathy by endoscopy or contrast CT or MRI.
- Joints - Joint assessment (Range of motion, X Rays, MRI or Ultrasound for effusion or synovitis)

**Body Composition and Muscle Function:**

- DEXA Lean body mass and fat-free mass
- Appendicular skeletal muscle mass
- Functional tests (gait speed, chair rise, Timed Up and Go (TUG), Short performance physical battery (SPPB), 30-second chair stand test, modified Bruce protocol- treadmill for adults, Incremental exercise test with cycle ergometer, Wingate cycle test, Astrand cycle test )

**Biomarkers:**

- Inflammatory markers
- Oxidative stress biomarkers
- Myositis-specific antibodies

**Imaging and Electrophysiology:**

- Muscle MRI (STIR, fat quantification, TMV)
- Electromyography
- Muscle shear wave elastography
- Muscle Ultrasound

**Patient-Reported Outcomes:**

- Quality of life measures - Short Form 36 Health Survey (SF-36), Pediatric Quality of Life Inventory (PedsQoL), EuroQoL Dimension Questionnaire(EQ-5D), Child Health Questionnaire- Parent Form 50 (CHQ-PF50), Patient Acceptable Symptom State (PASS), PROMIS pediatric measures, KINDL Revised Quality of Life Questionnaire, Child Dermatology Life Quality Index (CDLQI), SKindex 16, 29, 29+3; DLQI, CHQ, DGCM, PedsQoL, Quality of My Life (QoML), World Health Organisation- Quality of Life- Brief Version (WHOQOL-BREF)
- Fatigue scales - Functional Assessment of Chronic Illness Therapy (FACIT), Fatigue Severity Scale (FSS), Visual Analog Scale (VAS), EQ-5D
- Pain assessments (Visual Analog Scale (VAS), PROMIS measures, Pain disability index)
- Anxiety and depression (Hospital Anxiety and Depression Scale (HADS), Beck Depression Inventory (BDI-II), Generalized Anxiety Disorder (GAD-7), PROMIS scores, Patient Health Questionnaire (PHQ-9) Pediatric Symptom Checklist (PSC-17), Screen for Child Anxiety Related Emotional Disorders (SCARED)
- Work productivity (WPAI)

**Types of Studies**

- Randomized controlled trials
- Guidelines, SLRs, and MA will be screened for cross-references to identify any missed articles

**Exclusion Criteria:**

- Review articles and editorials
- Non-randomized controlled trials
- Conference proceedings
- Case reports/series
- Animal and in-vitro studies

**Supplementary Data S2: Detailed Search Strings**

**Pubmed: 4/18/25 - 803 results**

Filters - RCT, English, Humans

(

  "Muscular Diseases"[Mesh] OR "Myositis"[Mesh] OR "Polymyositis"[Mesh] OR "Dermatomyositis"[Mesh] OR "Inclusion Body Myositis"[Mesh] OR "Myopathies"[Mesh] OR "Muscular Dystrophies"[Mesh] OR "Muscular Dystrophy, Facioscapulohumeral"[Mesh] OR "Muscular Dystrophy, Emery-Dreifuss"[Mesh] OR "Myotonic Dystrophy"[Mesh] OR "Myotonia Congenita"[Mesh] OR "Mitochondrial Myopathies"[Mesh] OR "Congenital Myopathies"[Mesh] OR "Lipid Metabolism, Inborn Errors"[Mesh] OR "Glycogen Storage Disease Type II"[Mesh] OR "Glycogen Storage Disease Type V"[Mesh] OR "Distal Myopathies"[Mesh] OR "Limb-Girdle Muscular Dystrophy"[Mesh] OR "Nemaline Myopathy"[Mesh] OR "Myofibrillar Myopathy"[tiab] OR "GNE Myopathy"[tiab] OR "Multisystem Proteinopathy"[tiab] OR "Statin Myopathy"[tiab] OR "myopathy"[tiab] OR "myopathies"[tiab] OR "muscle disease"[tiab] OR "muscle diseases"[tiab] OR “Cancer associated myopathy”[tiab] OR "Critical illness myopathy"[tiab] OR  "Amyopathic dermatomyositis"[tiab] OR "Overlap Myositis"[tiab] OR  “Inclusion body myositis”[tiab] OR "Sporadic Inclusion Body Myositis"[tiab] OR "Immune-Mediated Necrotizing Myopathy"[tiab] OR "Antisynthetase Syndrome"[tiab] OR "Autoimmune Myopath*"[tiab] OR "Congenital Muscular Dystrophy"[tiab] OR "Centronuclear Myopathy"[tiab] OR  “Facioscapulohumeral muscular dystroph*”[tiab]  OR  “Juvenile dermatomyositis”[tiab] OR “Juvenile myositis”[tiab] OR “Polymyositis-dermatomyositis”[tiab] OR “Dermatopolymyositis”[tiab] OR “Adult type dermatomyositis”[tiab] OR “Childhood type dermatomyositis”[tiab] OR “Myositides”[tiab] OR “Inflammatory muscle disease”[tiab] OR “Inflammatory myopathy”[tiab] OR “Inflammatory myopathies”[tiab] OR “Juvenile idiopathic inflammatory myopath*”[tiab] OR “Focal myositis”[tiab]  OR “Proliferative myositis”[tiab] OR “Idiopathic inflammatory myopath*”[tiab] OR  “Myotubular myopath*”[tiab] OR "Inflammatory Myopathies"[tiab] OR “Metabolic myopath*”[tiab] OR "Non-inflammatory Myopathies"[tiab]

)

AND

(

  "Diet"[Mesh] OR "Diet, Mediterranean"[Mesh] OR "Diet, Vegetarian"[Mesh] OR "Diet, Ketogenic"[Mesh] OR “Dietary protein”[tiab] OR “Dietary protein”[Mesh] OR “Dietary fats”[tiab] OR “Dietary fats”[Mesh] OR "Diet, High-Fat"[Mesh] OR "Diet, Carbohydrate-Restricted"[Mesh] OR "Diet, Macrobiotic"[Mesh] OR "Diet Therapy"[Mesh] OR "Elimination Diets"[Mesh] OR "Feeding Behavior"[Mesh] OR "Feeding Methods"[Mesh] OR "Nutrition Therapy"[Mesh] OR "Nutritional Status"[Mesh] OR "Micronutrients"[Mesh] OR "Nutritional Supplements"[Mesh] OR “Soy”[tiab] OR  "Soy Foods"[Mesh] OR "Vitamin Supplementation"[Mesh] OR "Polyphenols"[Mesh] OR "Probiotics"[Mesh] OR "Prebiotics"[Mesh] OR "Synbiotics"[Mesh] OR "Fasting"[Mesh] OR "Caloric Restriction"[Mesh] OR "Fatty Acids, Omega-3"[Mesh] OR "Amino Acids"[Mesh] OR "Dietary Proteins"[Mesh] OR "Dietary Fiber"[Mesh] OR "FODMAP Diet"[Mesh] OR "Dietary Approaches To Stop Hypertension"[Mesh] OR "Caffeine"[Mesh] OR  “High protein Low carbohydrate diet”[tiab] OR “Intermittent fasting”[tiab] OR “Intermittent fasting”[Mesh] OR “High Fructose Corn Syrup"[Mesh] OR “High Fructose Corn Syrup"[tiab] OR "Alcohol Drinking"[Mesh] OR "Smoking"[Mesh] OR "Curcumin"[Mesh] OR "Vitamin D"[Mesh] OR "Coenzyme Q10"[Mesh] OR "Spirulina"[Mesh] OR "Inflammation/diet therapy"[Mesh] OR "Food, Processed"[Mesh] OR "Food, Formulated"[Mesh] OR "Myositis/diet therapy"[Mesh] OR "Creatine/therapeutic use"[Mesh] OR "Acetylcarnitine/therapeutic use"[Mesh] OR "Glutamine/therapeutic use"[Mesh] OR "Carnitine/therapeutic use"[Mesh] OR "Nordic diet"[tiab] OR "MIND diet"[tiab] OR "Paleo diet"[tiab] OR "Atkins diet"[tiab] OR "Ayurvedic diet"[tiab] OR "Berries"[tiab] OR "Whey protein"[tiab] OR "Anti-inflammatory diet"[tiab] OR "Raw food diet"[tiab] OR "Lacto-ovo-vegetarian diet"[tiab] OR "omega-3"[tiab] OR  "omega-6"[tiab] OR "polyphenol"[tiab] OR "fasting"[tiab] OR "caloric restriction"[tiab] OR "curcumin"[tiab] OR "vitamin D"[tiab] OR "coenzyme Q10"[tiab] OR "creatine"[tiab] OR "glutamine"[tiab] OR "acetylcarnitine"[tiab] OR "carnitine"[tiab] OR "Nutraceuticals"[tiab] OR "Resveratrol"[tiab] OR “Red yeast rice”[tiab] OR “Oyster mushrooms”[tiab] OR “Branched chain amino acids”[tiab] OR "Amino Acids, Branched-Chain"[Mesh]

)

**Embase: 244 (4/24/25)**

<1974 to 2025 April 24>

| **#** | **Query** | **Results** |
| --- | --- | --- |
| 1 | myositis.ab,kf,ti. | 21,006 |
| 2 | exp myositis/ | 57,891 |
| 3 | *mitochondrial myopathy/ or *distal myopathy/ or *critical illness myopathy/ or *nemaline myopathy/ or *idiopathic inflammatory myopathy/ or *centronuclear myopathy/ or *autoimmune necrotizing myopathy/ or *inclusion body myopathy/ | 5,060 |
| 4 | exp dermatomyositis/ or exp amyopathic dermatomyositis/ or exp adult dermatomyositis/ or exp juvenile dermatomyositis/ | 21,537 |
| 5 | (dermatomyositis or Juvenile dermatomyositis or Adult dermatomyositis or Amyopathic dermatomyositis or inclusion body myositis or Overlap myositis or scleromyositis or Necrotizing myopathy or Immune mediated necrotizing myopathy or Statin induced myopathy or anti-HGMCR Myopathy or Anti-SRP myopathy).ab,kf,ti. | 21,397 |
| 6 | exp polymyositis/ | 10,473 |
| 7 | polymyositis.ab,kf,ti. | 9,971 |
| 8 | *overlap myositis/ | 16 |
| 9 | exp antisynthetase syndrome/ | 2,146 |
| 10 | (antisynthetase syndrome or Critical illness myopath* or distal myopath* or mitochondrial myopath* or metabolic myopathy* or Myotonic dystrophy or Muscular dystrophy or Facioscapulohumeral dystrophy or Limb-girdle muscular dystrophy or Emery-Dreifuss muscular dystrophy or oculopharyngeal muscular dystrophy or dystrophinopathy or Non-dystrophic myotonia or Congenital myopath* or Congenital myotonic dystrophy or Metabolic myopath* or Lipid myopathies or Glycogen storage disease myopath* or McArdle's disease or Pompes disease or Cori's disease or Myofibrillary myopathy or GNE myopathy or Multisystem proteinopathy 1-4).ab,kf,ti. | 55,474 |
| 11 | exp muscular dystrophy/ | 55,875 |
| 12 | *myotonic dystrophy/ | 6,771 |
| 13 | 1 or 2 or 3 or 4 or 5 or 6 or 7 or 8 or 9 or 10 or 11 or 12 | 130,546 |
| 14 | (High protein diet or low protein diets or Casein-free diet or Protein supplements or Whey protein or Pea protein or Soy protein or Casein or Amino acids or Leucine or BCAAs or Branched-Chain Amino Acids or Glutamine or HMB or Creatine or Carbohydrate-restricted diet or High-carbohydrate diets or FODMAP).ab,kf,ti. | 485,223 |
| 15 | (Lactose-free diet or High fructose diet or High saturated fat or trans-fat diet or Low-fat diet or Ketogenic diet or high-fat diet or Omega-6 fatty acids or Omega-3 fatty acids or Fish oils or Vitamins or Vitamin A or Vitamin C or Vitamin D or Vitamin E or Folic Acid or Vitamin B12 or Vitamin B2 or Calcium or Zinc or Magnesium or Potassium or Selenium or Biotin or Dietary Supplements or Anti-inflammatory Supplements).ab,kf,ti. | 1,361,521 |
| 16 | (Turmeric or Curcumin or Ginger or Antioxidants or CoQ10 or Green tea or Black tea or Dark chocolate or Herbs or Berries or Citrus fruits or Red wine or Legumes or Purple grapes or Nuts or Isoflavones or Probiotic or Prebiotics or Synbiotics or Postbiotics or Polyphenols or Red yeast rice or Oyster mushrooms or Spirulina or Plant-based diet or High-fiber diets or Mediterranean diet or Nordic diet or Gluten-free diet or Anti-inflammatory diet or Calorie-restricted diets or DASH diet or MIND diet or Immunonutrition diet or Lacto-ovo-vegetarian diet or Modified Atkins diet or Alternative medicine diet or Traditional Medicine Diet or Paleo diet or Ayurvedic diet or Traditional Chinese medicine diet).ab,kf,ti. | 320,709 |
| 17 | (Paleo diet or Ayurvedic diet or Traditional Chinese medicine diet or Raw food diet or Alkaline diet or Berry-based Diet or Raspberries or Blueberries or Aronia or Elderberry or Processed diets or Unbalanced Diets or Processed food or Ultraprocessed food or vegan diets or Pescatarian diets or Calorie Restricted diet or Sodium restricted diet or Intermittent fasting or Caffeine or Alcohol or Smoking).ab,kf,ti. | 869,961 |
| 18 | exp 5:2 diet/ or exp vegetarian diet/ or exp diet/ or exp alkaline diet/ or exp lactoovovegetarian diet/ or exp Mediterranean diet/ or exp pescovegetarian diet/ or exp "high fat/high sucrose diet"/ or exp "high fat/high glucose diet"/ or exp "high fat/high fructose diet"/ or exp macrobiotic diet/ or exp Nordic diet/ or exp MIND diet/ or exp diet restriction/ or exp diet therapy/ or exp FODMAP diet/ or exp lactose free diet/ or exp vegan diet/ | 806,597 |
| 19 | exp sodium restriction/ or exp DASH diet/ or exp diet therapy/ | 456,456 |
| 20 | exp vitamin/ | 807,648 |
| 21 | exp probiotic agent/ | 63,766 |
| 22 | exp antioxidant/ | 494,269 |
| 23 | biotin.ab,kf,ti. | 36,617 |
| 24 | exp processed food/ | 9,520 |
| 25 | *creatine/ | 3,923 |
| 26 | exp diet supplementation/ | 107,828 |
| 27 | immunonutrition/ | 1,043 |
| 28 | exp soybean protein/ | 8,258 |
| 29 | exp whey protein/ | 5,876 |
| 30 | exp plant protein/ or exp pea protein/ | 181,665 |
| 31 | exp casein/ | 19,697 |
| 32 | exp mineral intake/ | 29,823 |
| 33 | exp potassium intake/ | 3,606 |
| 34 | 14 or 15 or 16 or 17 or 18 or 19 or 20 or 21 or 22 or 23 or 24 or 25 or 26 or 27 or 28 or 29 or 30 or 31 or 32 or 33 | 4,259,940 |
| 35 | limit 34 to (human and english language and randomized controlled trial and (english or hindi)) | 117,905 |
| 36 | limit 35 to (human and randomized controlled trial and (english or hindi)) | 117,905 |
| 37 | 13 and 34 | 16,292 |
| 38 | limit 37 to (human and randomized controlled trial and (english or hindi)) | 244 |

**Cochrane: 40 (4/25/25)**

#1 ( "muscular disease" OR "muscle diseases" OR myopathy OR myopathies OR "myositis" OR "polymyositis" OR "dermatomyositis" OR "inclusion body myositis" OR "immune-mediated necrotizing myopathy" OR "antisynthetase syndrome" OR "autoimmune myopathy" OR "autoimmune myopathies" OR "congenital muscular dystrophy" OR "limb-girdle muscular dystrophy" OR "facioscapulohumeral muscular dystrophy" OR "emery-dreifuss muscular dystrophy" OR "myotonic dystrophy" OR "mitochondrial myopathy" OR "glycogen storage disease" OR "juvenile dermatomyositis" OR "juvenile myositis" OR "idiopathic inflammatory myopathy" OR "inflammatory myopathy" OR "inflammatory myopathies" OR "myofibrillar myopathy" OR "centronuclear myopathy" OR "myotubular myopathy" OR "statin myopathy" OR "critical illness myopathy" OR "cancer associated myopathy" OR "distal myopathy" OR "nemaline myopathy" OR "overlap myositis" OR "multisystem proteinopathy" OR "Non-inflammatory Myopathies" OR "Metabolic myopathies" OR "Mitochondrial myopathies" ) 2924

#2 ( "diet" OR "mediterranean diet" OR "vegetarian diet" OR "ketogenic diet" OR "high protein diet" OR "low carbohydrate diet" OR "macrobiotic diet" OR "elimination diet" OR "nutrition therapy" OR "nutritional supplements" OR "micronutrients" OR "feeding behavior" OR "fasting" OR "intermittent fasting" OR "caloric restriction" OR "omega-3" OR "omega-6" OR "polyphenols" OR "probiotics" OR "prebiotics" OR "synbiotics" OR "vitamin D" OR "coenzyme Q10" OR "creatine" OR "glutamine" OR "carnitine" OR "acetylcarnitine" OR "nutraceuticals" OR "resveratrol" OR "red yeast rice" OR "oyster mushrooms" OR "branched chain amino acids" OR "whey protein" OR "anti-inflammatory diet" OR "paleo diet" OR "atkins diet" OR "ayurvedic diet" OR "mind diet" OR "nordic diet" OR "caffeine" OR "alcohol" OR "smoking" OR "soy" OR "berries" OR "processed food" OR "formulated food" OR "FODMAP" OR "DASH diet") 239615

#3	#1 AND #2 668

#4	Randomised controlled trials 1373438

#5	MeSH descriptor: [Myositis] explode all trees 331

#6	MeSH descriptor: [Dermatomyositis] explode all trees 154

#7	MeSH descriptor: [Muscular Dystrophies] explode all trees 688

#8	MeSH descriptor: [Distal Myopathies] explode all trees 14

#9	MeSH descriptor: [Myositis, Inclusion Body] explode all trees 49

#10	MeSH descriptor: [Muscular Dystrophies] explode all trees 688

#11	MeSH descriptor: [Muscular Dystrophy, Facioscapulohumeral] explode all trees 45

#12	MeSH descriptor: [Muscular Dystrophy, Emery-Dreifuss] explode all trees 0

#13	MeSH descriptor: [Myotonic Dystrophy] explode all trees 108

#14	MeSH descriptor: [Myotonia Congenita] explode all trees 14

#15	MeSH descriptor: [Mitochondrial Myopathies] explode all trees 64

#16	MeSH descriptor: [Myopathies, Structural, Congenital] explode all trees 7

#17	MeSH descriptor: [Distal Myopathies] explode all trees 14

#18	MeSH descriptor: [Muscular Dystrophies, Limb-Girdle] explode all trees 16

#19	MeSH descriptor: [Myopathies, Nemaline] explode all trees 2

#20	MeSH descriptor: [Glycogen Storage Disease Type II] explode all trees 65

#21	MeSH descriptor: [Glycogen Storage Disease Type V] explode all trees 38

#22	MeSH descriptor: [Lipid Metabolism, Inborn Errors] explode all trees 1249

#23	(Antisynthetase Syndrome):ti,ab,kw 38

#24	(“Multisystem Proteinopathy”):ti,ab,kw 0

#25	(“Autoimmune Myopathies”):ti,ab,kw 9

#26	(overlap myositis):ti,ab,kw 16

#27	(“Statin Myopathy”):ti,ab,kw 11

#28	(“Critical illness myopathy”):ti,ab,kw 27

#29	(“Cancer-associated myopathy”):ti,ab,kw 0

#30	#5 OR #6 OR #7 OR #8  #9 OR #10 OR #11 OR #12 OR #13 OR #14 OR #15 OR #16 OR #17 OR #18 OR #19 #20 OR #21 OR #22 #23 OR #24 OR #25 OR #26 OR #27 OR #28 OR #29 1187

#31	MeSH descriptor: [Diet] explode all trees 26389

#32	MeSH descriptor: [Diet, Mediterranean] explode all trees 1011

#33	MeSH descriptor: [Diet, Ketogenic] explode all trees 232

#34	MeSH descriptor: [Diet, Vegetarian] explode all trees 351

#35	MeSH descriptor: [Diet, High-Fat] explode all trees 386

#36	MeSH descriptor: [Diet, Carbohydrate-Restricted] explode all trees 868

#37	MeSH descriptor: [Diet, Macrobiotic] explode all trees 6

#38	MeSH descriptor: [Elimination Diets] explode all trees 27

#39	MeSH descriptor: [Diet Therapy] explode all trees 8419

#40	MeSH descriptor: [Nutrition Therapy] explode all trees  13131

#41	MeSH descriptor: [Nutritional Status] explode all trees 3793

#42	MeSH descriptor: [Micronutrients] explode all trees 7133

#43	MeSH descriptor: [Dietary Supplements] explode all trees 19714

#44	MeSH descriptor: [Feeding Behavior] explode all trees 13146

#45	MeSH descriptor: [Feeding Methods] explode all trees 4413

#46	MeSH descriptor: [Caloric Restriction] explode all trees 1241

#47	MeSH descriptor: [Fasting] explode all trees 4604

#48	MeSH descriptor: [Fatty Acids, Omega-3] explode all trees 4240

#49	MeSH descriptor: [Amino Acids, Essential] explode all trees 6498

#50	MeSH descriptor: [Amino Acids, Branched-Chain] explode all trees 2955

#51	MeSH descriptor: [Dietary Fiber] explode all trees 2719

#52	MeSH descriptor: [Dietary Proteins] explode all trees 5402

#53	MeSH descriptor: [Dietary Fats] explode all trees 9743

#54	MeSH descriptor: [Polyphenols] explode all trees 1100

#55	MeSH descriptor: [Probiotics] explode all trees 3772

#56	MeSH descriptor: [Prebiotics] explode all trees 784

#57	MeSH descriptor: [Synbiotics] explode all trees 315

#58	MeSH descriptor: [Soy Foods] explode all trees 719

#59	MeSH descriptor: [Vitamins] explode all trees 5719

#60	MeSH descriptor: [Vitamin D] explode all trees 7711

#61	MeSH descriptor: [Caffeine] explode all trees 2954

#62	MeSH descriptor: [Alcohol Drinking] explode all trees 5931

#63	MeSH descriptor: [Smoke] explode all trees 601

#64	MeSH descriptor: [Curcumin] explode all trees 731

#65	MeSH descriptor: [Ubiquinone] explode all trees 714

#66	MeSH descriptor: [Creatine] explode all trees 960

#67	MeSH descriptor: [Acetylcarnitine] explode all trees 163

#68	MeSH descriptor: [Glutamine] explode all trees 834

#69	MeSH descriptor: [Carnitine] explode all trees 818

#70	MeSH descriptor: [Food, Processed] explode all trees 33

#71	MeSH descriptor: [Food, Formulated] explode all trees 1717

#72	MeSH descriptor: [FODMAP Diet] explode all trees 17

#73	MeSH descriptor: [Immunonutrition Diet] explode all trees 15

#74	(MIND diet):ti,ab,kw OR (Nordic diet):ti,ab,kw OR (Paleo diet):ti,ab,kw OR ("Atkins diets"):ti,ab,kw OR (Ayurvedic diet):ti,ab,kw 829

#75	(Anti-inflammatory diet):ti,ab,kw OR ("high protein, low carbohydrate diet"):ti,ab,kw 1794

#76	#31 OR #32 OR #33 OR #34 OR #35 OR #36 OR #37 OR #38 OR #39 OR #40 OR #41 OR #42 OR #43 OR #44 OR #45 OR #46 OR #47 OR #48 OR #49 OR #50 OR #51 OR #52 OR #53 OR #54 OR #55 OR #56 OR #57 OR #58 OR #59 OR #60 OR #61 OR #62 OR #63 OR #64 OR #65 OR #66 OR #67 OR #68 OR #69 OR #70 OR #71 OR #72 OR #73 OR #74 OR #75 89565

#77	#30 AND #76 104

#78	#3 AND #77 55

#79	#78 AND #4 40

**Supplementary Data S3: Details of included studies**

**Inflammatory Myopathies**

| **Chung et al; 2007** | |
| --- | --- |
| **Country** | UK, Sweden |
| **Study design** | Randomised controlled trial |
| **Age, years** | Mean: 59 (creatine), 50 (placebo) |
| **Disease** | Polymyositis and Dermatomyositis |
| **Inclusion Criteria** | 1) men and women ages >18 years; 2) diagnosis of polymyositis or dermatomyositis using the clinical, biochemical, histopathologic, and electrophysiology criteria of Bohan and Peter with disease durations 6 months and neither clinical nor histologic evidence of inclusion body myositis; 3) quadriceps muscle strength at least 20% below predicted values based on the judgment of the assessing clinician in the context of current knowledge of normal quadriceps function and changes with age; 4) sufficient mobility to participate in the study; 5) previous therapy with systemic steroids for at least 6 months with current oral prednisolone stable for 2 months; 6) stable patients whose disease activity was low by the physician’s assessment; and 7) stable doses of immunosuppressive drugs for 3 months (if used) |
| **Exclusion Criteria** | 1) a formal active exercise program within the last 6 months, 2) unable to exercise sufficiently to participate, 3) concomitant serious medical disorders such as renal and liver disease, 4) pregnancy or of childbearing age unless adequate contraceptive precautions were taken, and 5) metal implants (which could affect MRS measurements). |
| **Patient Characteristics** | Disease duration (median years): Study- 9.2 (1-52), Control- 8.6 (1-23) |
| **Intervention** | Creatine Supplements |
| **Intervention details** | Oral creatine (highly purified creatine monohydrate)- loading dosage of 20 gm/day for 8 days followed by a maintenance dosage of 3 gm/day + home exercise program 5 days per week |
| **Control** | Placebo + home exercise program 5 days per week |
| **N** | Study= 19, Control= 18 |
| **Follow-up period** | 6 months |
| **Outcomes assessed** | **Primary outcome**: aggregate functional performance time (AFPT) which aggregates 4 objective functional assessments measured in seconds: 50-foot timed walk; the get up and go test, which also involves a 50-foot walk; and a stair ascent test and stair descent test.  **Secondary outcomes**: functional index in myositis, manual muscle testing using the Medical Research Council extended (0–5)scale, serum creatine kinase (CK) levels, health status (using the Nottingham Health Profile [NHP], which has previously been used in myositis), the Short Form McGill Pain Questionnaire, the Hospital Anxiety and Depression Scales , the Chalder fatigue score, and adverse events (clinical questioning, full blood count, renal and liver function tests). Muscle bioenergetics was evaluated by 31P MRS. Levels of PCr-nucleoside triphosphate, phosphodiester, and inorganic phosphate (Pi) were evaluated. Intracellular pH in muscles was determined by the chemical shift of the Pi resonance relative to the PCr signal. |
| **Result (Primary outcome)** | Decrease in AFPT (median decrease 13%, range: -32-8%, p=0.014), represents improvement |
| **Result (Secondary outcome)** | Increase in Functional Index of myositis (from median score of 50.3 to 57.0, p=0.034); Significant differences in shoulder abduction and hip flexion measured by Manual Muscle Testing (MMT) (p<=0.05); PCr/B-NTP ratios increased by 3.4% (p=0.05); No change in NHP, Short Form McGill Pain Questionnaire, Hospital Anxiety and Depression Scales and Chalder fatigue scores |
| **Safety results** | 22 adverse events in 13 patients (6–7 in each group), including 8 infections, 6 falls, and 2 gastrointestinal events, unrelated to creatine treatment or other medication. No significant changes in laboratory data. |

| **Dover et al; 2021** | |
| --- | --- |
| **Country** | Canada |
| **Study design** | Randomised, multiple baseline trial |
| **Age, years** | Median (Range): 13 (7-14) |
| **Disease** | Juvenile Dermatomyositis (JDM) |
| **Inclusion Criteria** | 1) diagnosis of probable or definite JDM with onset age < 16 according to Bohan and Peter criteria, 2) on a stable course of medication unlikely to change over the study period, and 3) a minimum height of 132.5cm (to be able to use all the exercise testing equipment) |
| **Exclusion Criteria** | 1) newly diagnosed (within 6 months), were unable to cooperate with or too weak to participate in study procedures, had impaired kidney function as determined by screening laboratory values, or were pregnant or planning to become pregnant within the study period. |
| **Patient Characteristics** | Age at diagnosis- 7.5 (2.5-12) years |
| **Intervention** | Creatine Supplements |
| **Intervention details** | Creatine 150 mg/kg/day or 4.69 g/m2/day, in chewable, lemon-flavoured tablets (AlzChem) |
| **Control** | Placebo |
| **N** | 13 (all patients received intervention at different start times) |
| **Follow-up period** | 4 weeks- 6 months |
| **Outcomes assessed** | **Primary outcome**: feasibility (recruitment rate, attended versus missed study visits, proportion of completed study procedures, and adherence), safety and tolerability.  **Secondary outcomes**: muscle function using Wingate Anaerobic Test, disease activity using International Myositis Assessment and Clinical Studies (IMACS) group core set, aerobic capacity using YMCA cycle ergometer submaximal test, muscle metabolism using 31-Phosphorus Magnetic Resonance Spectroscopy (31P-MRS), muscle strength using maximal jump test and a handgrip strength test, fatigue using Pediatric Quality of Life (PedsQL) Fatigue Module and both overall and health-related QOL using the Quality of My Life (QoML) scale, physical activity using 3-day physical activity recall questionnaire (3DPAR), and treatment satisfaction using the Treatment Satisfaction Questionnaire for Medication (TSQM II). |
| **Result (Primary outcome)** | Feasibility and safety were demonstrated- good adherence (median adherence- 88.5%), no missed visits, no adverse events. |
| **Result (Secondary outcome)** | Improvement in muscle metabolism as seen by decrease in Pi/PCr, ratio (p=0.03) and decrease in change in pH after exercise (p=0.003); High satisfaction (median rating 83.3); No statistically significant changes in muscle function, muscle strength, aerobic capacity, disease activity, fatigue, physical activity, or QOL |
| **Safety results** | No adverse events. Transient reversible dehydration in 1 patient (elevated serum creatinine). |

| **Kimura et al; 2022** | |
| --- | --- |
| **Country** | Japan |
| **Study design** | Phase II/III, multi-centre, randomized controlled trial |
| **Age, years** | 20-75 |
| **Disease** | Polymyositis and Dermatomyositis |
| **Inclusion Criteria** | 1) 20–75 years with a diagnosis of definite or probable PM/DM 2) Average Manual Muscle Testing (MMT) scores >6 and <9.5 with elevated serum Creatine Kinase (CK) or aldolase levels above the upper limit of normal |
| **Exclusion Criteria** | 1) patients with other myopathies, paralysis, malignancies, Interstitial lung disease, hypothyroidism, severe heart or liver diseases, congenital amino-acid metabolism, dysphagia, allergy to Branched Chain Amino Acids (BCAA's) or glucocorticoids, h/o drug or alcohol abuse, women in childbearing age group, pregnant or breast feeding, 2) subjects judged ineligible by investigators and 3) subjects participated in other clinical trials within 6 months. |
| **Patient Characteristics** | Disease duration [days, mean (SD)]- Study: 212.2(259.6), Control: 169.8(107.4) |
| **Intervention** | Branched Chain Amino Acids |
| **Intervention details** | One package of TK-98 (4.15g) contained L-isoleucine 952mg, L-leucine 1904mg, and L-valine 1144mg (molar ratio is 1:2:1.35), and 6 packages were administered daily in 3 divided doses. |
| **Control** | Placebo |
| **N** | Study- 24, Control- 23 |
| **Follow-up period** | 12 weeks |
| **Outcomes assessed** | **Primary outcome**: muscle strength evaluated by MMT;  **Secondary outcome**: clinical response evaluated with Myositis Disease Activity Core Set (MDACS) and the change of functional index (FI) |
| **Result (Primary outcome)** | No significant change in MMT score [mean (S.E.M.) 0.70 (0.19) in the TK-98 group vs 0.69 (0.18) in the placebo group; p=0.98] |
| **Result (Secondary outcome)** | Increase in functional index (FI) score of shoulder flexion [27.9 (5.67) vs 12.8 (5.67) for the right shoulder flexion, and 27.0 (5.44) vs 13.4 (5.95) for the left shoulder; p<0.05], improvement in all tested motions (dynamic repetitive muscle functions); Minimal, moderate and major improvement in clinical response (MDACS) was achieved in 100%, 82.4% and 29.4% of the TK-98 group and 100%, 83.3% and 38.9% of the placebo group, with no differences between the two; |
| **Safety results** | Adverse events, such as heart failure, myocarditis, depression, suicide attempt, pneumocystis pneumonia, urolithiasis, interstitial lung disease, anorexia, odontectomy, were observed in over 90% of patients in the investigational phase with no significant difference between the two groups, most being attributed to the use of high-dose Glucocorticoids |

| **Solis et al; 2016** | |
| --- | --- |
| **Country** | Brazil |
| **Study design** | Randomised Crossover Design |
| **Age, years** | Mean (SD)- 4 |
| **Disease** | Juvenile Dermatomyositis (JDM) |
| **Inclusion Criteria** | 1) stable dose of corticosteroids or other immunomodulating medication (i.e., cyclosporine, methotrexate, or intravenous immunoglobulin) for at least 8 weeks; 2) prednisone dose 40 mg/day. |
| **Exclusion Criteria** | 1) macroalbuminuria; 2) glomerular filtration rate <30 ml/min/1.73 m2; 3) use of hormonal contraceptives; 4) current pregnancy; 5) diabetes mellitus; |
| **Patient Characteristics** | Active disease (n=5), Inactive disease (n-=10)  Disease duration [years, mean (SD)]- 7(3) |
| **Intervention** | Creatine Supplements |
| **Intervention details** | 0.1 g/ kg/day of creatine monohydrate |
| **Control** | Placebo |
| **N** | 15 (crossover) |
| **Follow-up period** | 12 weeks + 8 weeks (washout) |
| **Outcomes assessed** | **Primary outcome**: Muscle function (1-repetition maximum leg press, bench press, handgrip strength, timed up-and-go test, timed-stands test) **Secondary outcomes**: Muscle metabolism: Intramuscular phosphocreatine (PCr) content, Body composition and bone health: Lean mass, fat mass, body fat percentage, bone mineral content (BMC), bone mineral density (BMD) at the lumbar spine, total hip, and whole body, CTX, P1NP; Aerobic conditioning: (oxygen consumption) VO2 max, VO2 at ventilatory anaerobic threshold (VAT), VO2 at respiratory compensation point (RCP), time to exhaustion during treadmill test; Disease activity and physical function: Childhood Myositis Assessment Scale (CMAS), Manual Muscle Testing (MMT), Disease Activity Score (DAS), Childhood Health Assessment Questionnaire (CHAQ), Physician Visual Analog Scale (VAS), Patient Visual Analog Scale (VAS); Quality of life: Pediatric Quality of Life Inventory (PedsQL – patient and parent versions); Inflammatory markers: IL-6, IL-10, IL-17A, TNF-alpha, IFN-gamma; Safety outcomes: Kidney function (51Cr-EDTA clearance), routine lab parameters, self-reported side effects, adverse events |
| **Result (Primary outcome)** | No significant change in muscle function; no interaction effect for any test (P>0.05). Between-condition differences (creatine vs placebo): 1-RM leg press −1.1 kg (95% CI −4.3 to 2.3; P=0.48); 1-RM bench press −0.5 kg (95% CI −0.8 to 1.8; P=0.43); handgrip −0.2 kg (95% CI −1.8 to 2.2; P=0.84); timed-stands −0.8 reps (95% CI −1.7 to 0.1; P=0.08); timed up-and-go −0.1s (95% CI −0.0 to 0.4; P=0.17); VO₂max −0.9 ml/kg/min (95% CI −2.9 to 3.4; P=0.45); time to exhaustion −42.0s (95% CI −60.6 to 72.7; P=0.85) |
| **Result (Secondary outcome)** | No significant change in intramuscular PCr content (95% CI for delta score −2.8±2.4; P=0.45). Body composition: all between-condition differences non-significant — lean mass 0.5 kg (95% CI −1.3 to 0.3; P=0.46); BMC −6.07g (95% CI −27.2 to 39.4; P=0.07); BMD lumbar spine 0.00 g/cm² (95% CI −0.04 to 0.03; P=0.23). Disease activity and functional capacity: CMAS −0.27 (95% CI −2.0 to 1.5; P=0.75); MMT −0.13 (95% CI −0.7 to 0.4; P=0.61); CHAQ 0.02 (95% CI −0.1 to 0.1; P=0.74); DAS −0.07 (95% CI −1.7 to 1.5; P=0.93); PedsQL patient −0.24 (95% CI −8.3 to 7.8; P=0.95) |
| **Safety results** | No adverse events or changes in laboratory parameters |

**Muscular Dystrophies**

| **Andersen et al; 2015** | |
| --- | --- |
| **Country** | Denmark |
| **Study design** | Randomised controlled trial |
| **Age, years** | Mean: Protein=42.6, Placebo=45.7, Control=51.3 |
| **Disease** | Facioscapulohumeral Muscular Dystrophy (FSHD) |
| **Inclusion Criteria** | 1)Adults 18-65 years of age, 2) Genetically verified FSHD type 1 (<10 D4Z4 repeats in leukocytes DNA). |
| **Exclusion Criteria** | 1)Regular cardio exercise (<2 hours/week), 2) pregnancy or breastfeeding, 3) inability to cycle, or 4) other disabilities than FSHD, which could confound the interpretation of the results. |
| **Patient Characteristics** | MFSHD clinical score: Protein=5.2, Placebo=6.2, Control=5.9 |
| **Intervention** | Aerobic training and postexercise protein- carbohydrate supplementation |
| **Intervention details** | Participants trained 3 times weekly (36 sessions) for 30 minutes on an upright stationary bike. Protein-carbohydrate beverage 163 kcal (23 g whey protein, 17 g carbohydrate, 0 g fat) or placebo beverage- Sukrin (based on the alcohol, erythritol-0 kcal, 33 g carbohydrate, 0 g fat, 0 g protein) was consumed 15 minutes after each session. |
| **Control** | No intervention |
| **N** | Study: Training +protein supplement=18, training +placebo supplement=13, Control (no intervention)=10 |
| **Follow-up period** | 12 weeks |
| **Outcomes assessed** | **Primary Outcome**: Aerobic training- maximum oxygen consumption and workload (VO2max, Wmax) using cycle ergometer and walking speed using the 6 Minute Walk Test (6MWT),  Secondary Outcomes: mobility by (1) 5-times sit-to-stand-test, (2) 14-step-stair-test performed first at usual pace and then as fast as possible, (3) standing balance test,12 (4) self-assessed physical questionnaire, and (5) testing of knee and elbow flexion and extension strength with a custom made dynamometer testing box, Quality Of Life (QOL) by Short Form 36- Health Survey (SF-36) and grading fatigue on a scale, daily activity level by Bouchard diary and accelerometer |
| **Result (Primary outcome)** | Training increased fitness by 9.5% (CI 4–15) (p <0.002), workload by 17.7% (CI 10–26) (p <0.001), and walking speed by 7.4% (CI 4-11) (p<0.001). Protein-carbohydrate supplementation did not improve fitness, workload, or walking speed further than training alone. |
| **Result (Secondary outcome)** | Training improved self-assessed health (SF-36 questionnaire) in 2 domains: role limitations due to physical health (p=0.04) and role limitations due to emotional problems (p=0.001). Training did not change the level of pain and fatigue, muscle strength or daily activity level. Protein-carbohydrate supplementation did not result in further improvements in any tests compared to training alone. |
| **Safety results** | No adverse effects observed. Back pain during cycle sessions and diarrhoea in placebo group (known side effect of Sukrin) |

| **Banerjee et al; 2010** | |
| --- | --- |
| **Country** | India |
| **Study design** | Randomised controlled trial |
| **Age, years** | Mean (SD): Study- 7(1.2), Control- 7.2 (1.1) |
| **Disease** | Duchenne Muscular Dystrophy (DMD) |
| **Inclusion Criteria** | 1)Ambulatory and 2) steroid naive DMD patients (diagnosis based on a compatible clinical history, muscle biopsy suggestive of DMD and absence of immunohistochemical staining for dystrophin including antibodies to rod, amino and carboxy terminus.) |
| **Exclusion Criteria** | 1)Non-ambulatory, 2) on steroids or 3) with renal dysfunction as evidenced by raised serum urea and creatinine |
| **Patient Characteristics** | Age at onset of symptoms, years [Mean(SD)]: Study- 4.5 (2.1), Control 4.4 (1.7) |
| **Intervention** | Creatine Supplements |
| **Intervention details** | Oral creatine monohydrate (Coach's formula™, 99% pure creatine) 5 g/day in three divided doses |
| **Control** | Placebo (500 mg of vitamin C) |
| **N** | Study= 18, Control= 15 |
| **Follow-up period** | 8 weeks |
| **Outcomes assessed** | **Primary Outcome**: Cellular energetics using 31P MRS,  **Secondary Outcomes**: Muscle strength by Manual Muscle Testing (MMT) score, functional status using Vignos' functional scale, Parents’ report |
| **Result (Primary outcome)** | Increase in mean phosphocreatine (PCr)/inorganic phosphate (Pi) ratio in patients treated with Cr (4.7; 95% CI; 3.9–5.6) as compared to the placebo group (3.3; 95% CI; 2.5–4.2) (p=0.03). |
| **Result (Secondary outcome)** | Mean MMT score (6.2; 95%CI 5.9–6.5) was significantly higher(p=0.04) compared to the placebo group (5.8; 95%CI 5.5–6.1). Parents reported subjective improvement on Cr supplementation versus worsening on placebo (p=0.02). No change in functional scale (p=0.19) was observed. |
| **Safety results** | No adverse effects observed. |

| **Davidson et al; 2021** | |
| --- | --- |
| **Country** | Australia |
| **Study design** | Randomised controlled crossover trial |
| **Age, years** | Mean (SD): Standard- 8.7(2), Enhanced-9.4(2.6) |
| **Disease** | Duchenne Muscular Dystrophy (DMD) |
| **Inclusion Criteria** | 1) Boys aged 5-13 years 2) a universal diagnosis of DMD (based on the onset of characteristic clinical symptoms or signs by 9 years of age, an elevated serum Creatine Kinase (CK), and ongoing difficulty with ambulation, with documentation of a mutation in the dystrophin gene, or absence of dystrophin on muscle biopsy) 3) stable on a corticosteroid dose for 6 months(n=1 was steroid naive) and 4) able to walk >75 m unassisted during a 6 min walk test (6MWT) |
| **Exclusion Criteria** | 1)Use of personal assistance or assistive devices required to aid walking or use of a wheelchair/motorised scooter/ similar mobility aid for >75% of activity during the day; 2) cow's milk protein allergy or lactose intolerance; 3) history of kidney or liver disease; 4) unable to attend the seven required hospital appointments within the 12 month period;5) unable to swallow tablets; 6) parents wishing to continue using other or additional complementary therapies (including other nutritional supplements); 7) participation in other muscular dystrophy trials involving an active intervention;8) and use of medications contraindicated due to possible supplemented drug interactions |
| **Patient Characteristics** | - |
| **Intervention** | Multicomponent Nutritional Supplement |
| **Intervention details** | Enhanced supplement- Standard supplement plus: Creatine monohydrate (5 g), glutamine (0.6 g/kg fat free mass), and HMB (38 mg/kg/day) |
| **Control** | Standard supplement- protein shake (22 g whey protein), multivitamin capsules, vitamin D capsules (Vitamin D3, 2000 IU) and fish oil capsules (1050mg omega-3 triglycerides |
| **N** | Enhanced=16, Standard=20 (crossover) |
| **Follow-up period** | 20 weeks each+ 2 week washout |
| **Outcomes assessed** | **Primary Outcome**: 6-min walk distance (6MWD) and community ambulation (StepWatch™ Activity Monitoring)  **Secondary Outcomes**: anthropometric measures (weight, height), body composition (DXA scan), activity limitations measured by the Activity Limitation (ACTIVLIM) questionnaire, quality of life using PedsQl-core and neuromuscular modules, serum 25-hydroxyvitamin D. |
| **Result (Primary outcome)** | Enhanced supplement compared to the Standard supplement was associated with a difference of +12 (95% CI: -16, 40, p=0.37) metres in 6MWD, +0.5 (95% CI: -53, 54, p=0.98) inactive minutes per day and -95 (95% CI: -887, 696, p=0.81) steps per day. A mixed effect model indicated a potentially clinically important effect of the Enhanced supplement on the 6MWD of +31 (95% CI: -19, 81) metres. |
| **Result (Secondary outcome)** | Mean serum 25 hydroxyvitamin D levels at week 50 was 94 (95% CI: 84, 104) nmol/L. No effect of either supplement regime on body composition or quality of life. |
| **Safety results** | No serious adverse events observed. Rash(n=3),elevated blood pressure (n=1), resting tachycardia (n¼1),mild tachycardia (n=2), weight gain (n=2), respiratory infection (n=1), gastrointestinal illness(n=1),ear and throat infection(n=1). |

| **Escobar-Cedillo et al; 2013** | |
| --- | --- |
| **Country** | Mexico |
| **Study design** | Randomised controlled trial |
| **Age, years** | Median (range): Study- 7(4-10), Control- 6 (4-8) |
| **Disease** | Duchenne Muscular Dystrophy (DMD) |
| **Inclusion Criteria** | 1)Confirmed DMD diagnosis (by clinical, enzymatic and electrophysiological evaluations), 2) 4-9 years old, 3) functional activity score of <3 as per Vignos scale, 4) maximum wight of 40kg; |
| **Exclusion Criteria** | 1)Use of steroids, creatine, or related agents prior to the study and 2) comorbidities such as cardiac or respiratory conditions |
| **Patient Characteristics** | Disease onset, years [Median(range)]: Study- 3.5 (1-6), Control 2 (1-4) |
| **Intervention** | L-Carnitine Supplements |
| **Intervention details** | L-carnitine 50mg/kg twice a day |
| **Control** | Placebo |
| **N** | Study= 10, Control= 10 |
| **Follow-up period** | 1 year |
| **Outcomes assessed** | **Primary Outcomes**: Muscular evaluation by Manual Muscle Testing (MMT), Functional evaluation using Brooke scale, Swinyard classification and Vignos scale, |
| **Result (Primary outcome)** | No significant differences in muscular or functional evaluation (P>0.05) |
| **Result (Secondary outcome)** | - |
| **Safety results** | No adverse effects observed. |

| **Escolar et al; 2005** | |
| --- | --- |
| **Country** | Multinational |
| **Study design** | Randomised controlled three-arm trial |
| **Age, years** | Mean (SD): Creatine- 6.67(1.76), Glutamine- 6.32 (1.00), Control- 6.81 (1.33) |
| **Disease** | Duchenne Muscular Dystrophy (DMD) |
| **Inclusion Criteria** | 1)Ambulatory and 2) steroid naive DMD patients |
| **Exclusion Criteria** | 1) symptomatic cardiomyopathy; 2) ventricular arrhythmias; or 3) recent or current use of glutamine, Cr, or other supplements |
| **Patient Characteristics** | - |
| **Intervention** | Creatine or Glutamine Supplements |
| **Intervention details** | 5gm daily of Creatine (EAS, Golden, CA) powder or 0.3mg/kg glutamine twice daily (EAS) |
| **Control** | Placebo |
| **N** | Study: Creatine= 15, Glutamine=19, Control= 16 |
| **Follow-up period** | 6 months |
| **Outcomes assessed** | **Primary Outcome**: Muscle strength by Manual Muscle Testing (MMT) score  **Secondary Outcomes**: Quantitative muscle testing (QMT) of bilateral elbow flexors and extensors, knee flexors and extensors, and grip, combined QMT arm and leg scores, timed function tests (time to rise from supine position, to run/walk 10m and to climb four steps), pulmonary function tests (pulmonary forced vital capacity and negative inspiratory force). |
| **Result (Primary outcome)** | No statistically significant differences in MMT score in either group. |
| **Result (Secondary outcome)** | The creatine group showed less strength deterioration (p=0.07), as measured by QMT, than those on placebo. In the timed climbing test, the creatine group did better than the placebo group over time (p=0.015), and there was a similar trend for the glutamine group (p>0.05). Similar trends were observed with both glutamine and creatine in the timed running test. |
| **Safety results** | No adverse effects observed. |

| **Fenichel et al; 1988** | |
| --- | --- |
| **Country** | USA |
| **Study design** | Randomised controlled trial |
| **Age, years** | Mean (SD): Study- 11.4 (4.6), Control- 11.8(4.6) |
| **Disease** | Duchenne Muscular Dystrophy (DMD) |
| **Inclusion Criteria** | 1)Male, 2) Onset of weakness before 5 years, 3) Proximal weakness, 4) Increased serum Creatine Kinase |
| **Exclusion Criteria** | 1) Patients with ptosis or weakness of the external ocular muscles 2) Patients with any skin rash suggestive of dermatomyositis 3) Patients with sensory abnormalities (except coincident mononeuropathy, such as a pressure palsy due to sitting in a wheelchair).4) Normal muscle biopsy or muscle biopsy evidence of denervation, glycogen-storage disease, lipid storage disease, a morphologically distinct congenital myopathy, or an inflammatory myopathy. 5) Normal electromyograph (EMG) or with major evidence of denervation. 6) Any girls in the family who fulfil the major and minor criteria for diagnosis of DMD. |
| **Patient Characteristics** | - |
| **Intervention** | Penicillamine and vitamin E |
| **Intervention details** | Penicillamine: Patients less than 100lb- 125mg/day followed by 125mg twice a day followed by 250mg twice a day. Patients more than 100 lb- 250mg/day followed by 250 mg twice daily followed by 500mg twice a day.  Vitamin E: Patients less than 100 lb- 600 mg twice daily, patients more than 100 lb- 1200mg twice daily |
| **Control** | Placebo |
| **N** | Study= 52, Control= 54 |
| **Follow-up period** | 18 months |
| **Outcomes assessed** | **Primary Outcome**s: Muscle strength (average muscle score), functional tests assessing the level of activity of arm, hip, and leg function, timed-tests including traveling 30 ft (walking, running, or using a wheelchair), arising from the floor from a supine position, climbing four standard stairs, and arising from a standard chair, pulmonary function tests including forced vital capacity, maximum voluntary ventilation, and maximum expiratory pressure, blood was obtained for Creatine Kinase. |
| **Result (Primary outcome)** | No significant difference in muscle strength, functional grade, timed-functional tests, pulmonary function, and serum creatine kinase levels. |
| **Result (Secondary outcome)** | - |
| **Safety results** | Nausea and vomiting (n=8), anorexia (n=8), rash (n=14), taste disturbed (n=2), fever (n=3), mouth sore (n=7). Eight patients were withdrawn from the study because of adverse reactions to penicillamine. |

| **Hafner et al; 2019** | |
| --- | --- |
| **Country** | Switzerland |
| **Study design** | Randomised controlled trial |
| **Age, years** | Mean (SD): Study- 8.2(1.2), Control- 8.2 (1.0) |
| **Disease** | Duchenne Muscular Dystrophy (DMD) |
| **Inclusion Criteria** | 1)Age 6.5-10 years, 2) genetically confirmed DMD, 3) able to walk at least 150 m in 6 minutes and 4) had moderate motor function (≥40%, as measured by the first dimension [D1; pertaining to transfer and standing posture] of the Motor Function Measure [MFM] scale) |
| **Exclusion Criteria** | (1) had received an unstable corticosteroid treatment within the last 6 months; (2) were participants in other clinical trials; (3) had known intolerance or hypersensitivity to any study treatment; (4) had other chronic diseases or clinically relevant limitations of renal, liver, or heart function; (5) had a diagnosis of cancer or suspected cancer; or (6) Patients who received the combination therapy or their l-arginine metabolites within the last 3 months |
| **Patient Characteristics** | - |
| **Intervention** | L-citrulline and Metformin |
| **Intervention details** | 2500 mg of L-citrulline and 250 mg of metformin (combination therapy) 3 times a day for 26 weeks |
| **Control** | Placebo |
| **N** | Study= 23, Control= 24 |
| **Follow-up period** | 26 weeks |
| **Outcomes assessed** | **Primary Outcome**: Change in transfer and standing posture, as assessed by the first dimension of the Motor Function Measure  **Secondary Outcomes**: Overall motor function (total MFM score), proximal and axial motor functions (MFM D2), distal motor function (MFM D3), timed function tests, 6-minute walking distance, knee extension and elbow flexion (handheld dynamometry and quantitative muscle testing, biomarkers of muscle necrosis in plasma and urine, muscle degeneration (MRI) |
| **Result (Primary outcome)** | Motor function measure first dimension sub score decrease was 5.5% greater than placebo group (95% CI, −1.0% to12.1%; P = 0.09).  Reduction in motor function decline by was observed among the stable subgroup of patients treated with combination therapy (6.7%; 95% CI, 0.9%-12.6%; P = 0.03). |
| **Result (Secondary outcome)** | Muscle degeneration was significantly reduced (Difference in fat fraction- 1.7%, p=0.04, difference in T2-weighted relaxation time- 1.4 milliseconds, p=0.02). No significant differences in the overall, proximal and axial, or distal motor function assessments, in the timed function tests, muscle force evaluation, laboratory biomarkers |
| **Safety results** | No severe adverse effects observed. Mild transient gastrointestinal symptoms seen (n=4) |

| **Louis et al; 2003** | |
| --- | --- |
| **Country** | Italy |
| **Study design** | Randomised controlled crossover trial |
| **Age, years** | Mean (SD): 10.8 (2.8) |
| **Disease** | Duchenne Muscular Dystrophy (DMD) and Beckers dystrophy |
| **Inclusion Criteria** | 1)Diagnosed by clinical examination and identification of dystrophin gene mutation after muscle biopsy |
| **Exclusion Criteria** | 1)Receiving another drug therapy or 2) unable to develop a torque of at least 1 Nm during elbow flexion |
| **Patient Characteristics** | DMD (n=12), Becker dystrophy (n=3). 10 out of 15 patients were wheelchair dependent. |
| **Intervention** | Creatine Supplements |
| **Intervention details** | Creatine monohydrate- 3g daily |
| **Control** | Placebo (maltodextrin) |
| **N** | Study= 8, Control= 7 (crossover) |
| **Follow-up period** | 3 months each + 2months washout |
| **Outcomes assessed** | **Primary Outcomes**: Muscle function including maximal voluntary contraction (MVC), fatigue resistance, and evaluation of total joint stiffness (TJS), bone mineral density by dual energy X-ray absorptiometry, 31P Nuclear magnetic resonance spectroscopy, hepatic and renal function tests. |
| **Result (Primary outcome)** | In the patients receiving Cr MVC improved by 15% (p= 0.02), and fatigue resistance almost doubled (p= 0.001). No change in TJS. In patients who were able to walk, bone mineral density increased by 3% (p<0.05). No significant change in PCr/ATP ratio. Urinary excretion of collagen type I cross-linking N telopeptide declined to about one third (P 0.001) after Cr. |
| **Result (Secondary outcome)** | - |
| **Safety results** | No adverse effects observed. |

| **Mendell et al; 1984** | |  |
| --- | --- | --- |
| **Country** | USA | |
| **Study design** | Randomised controlled trial | |
| **Age, years** | Mean (SD): Study- 9.8, Control- 9.2 | |
| **Disease** | Duchenne Muscular Dystrophy (DMD) | |
| **Inclusion Criteria** | Inclusion must fulfil all major and 2 minor criteria. Major criteria: 1) Male patient. 2) Onset of weakness before the age of 5 years 3) Proximal weakness. 4) Increased serum creatine kinase (CK) activity at least 10 times the upper limit of normal at some stage in the illness. Minor: 1) The pedigree has 2 affected males who are not brothers (one may be the propositus), and the second affected male is the offspring of a female related to the mother of the propositus through maternal linkage. 2) The mother, sister, maternal aunt, or maternal grandmother of the propositus has an elevated CK level without there being other affected males in the family. 3) There are 2 or more affected brothers (1 may be the propositus) who fulfil the criteria of Duchenne dystrophy without there being other affected males in the family. 4). There is no family history of Duchenne dystrophy or elevated CK levels | |
| **Exclusion Criteria** | 1) Patients with ptosis or weakness of the external ocular muscles 2) Patients with any skin rash suggestive of dermatomyositis 3) Patients with sensory abnormalities (except coincident mononeuropathy, such as a pressure palsy due to sitting in a wheelchair).4) Normal muscle biopsy or muscle biopsy evidence of denervation, glycogen-storage disease, lipid storage disease, a morphologically distinct congenital myopathy, or an inflammatory myopathy. 5) Normal electromyograph (EMG) or with major evidence of denervation. 6) Any girls in the family who fulfil the major and minor criteria for diagnosis of DMD. | |
| **Patient Characteristics** | - | |
| **Intervention** | Leucine Supplements | |
| **Intervention details** | L-Leucine- 0.1 g/per kg twice daily | |
| **Control** | Placebo | |
| **N** | Study=47, Control= 44 | |
| **Follow-up period** | 12 months | |
| **Outcomes assessed** | **Primary Outcome**: Muscle strength by modified MRC score, joint contracture measurements by average contracture score, functional grade including level of activity of arm, hip and leg function, Timed functional tests (traveling 30 feet (walking, running, or using a wheelchair), arising from the floor from a supine position, climbing four standard stairs, arising from a standard chair, putting on a T-shirt, and cutting out a 3 x 3 inch square.), Pulmonary function tests (forced vital capacity (FVC), maximum voluntary ventilation (MVV), and maximum expiratory pressure (MEP)), Creatine kinase, serum leucine levels | |
| **Result (Primary outcome)** | No significant effect on muscle strength, joint contractures, timed-functional tests, pulmonary functions or serum CK levels. | |
| **Result (Secondary outcome)** | - | |
| **Safety results** | No serious adverse effects observed. Some gastrointestinal side effects seen- decreased appetite, anorexia, nausea, | |

| **Mok et al; 2009** | |
| --- | --- |
| **Country** | France |
| **Study design** | Randomised controlled crossover trial |
| **Age, years** | Median (Q1, Q3)- 6.1 (4.6, 8.2) |
| **Disease** | Duchenne Muscular Dystrophy (DMD) |
| **Inclusion Criteria** | 1) Paediatric neurologist diagnosis of DMD by clinical history, muscle biopsy (absence of dystrophin confirmed by immunohistochemistry or Western blot) and molecular biology, 2) the ability to walk ≥170 m. |
| **Exclusion Criteria** | 1)Wheelchair dependency, 2)3 body weight >60 kg, 3) hepatic or renal insufficiency, or 4) surgery scheduled in the year following the first visit. |
| **Patient Characteristics** | - |
| **Intervention** | Glutamine Supplements |
| **Intervention details** | Glutamine (0.5 g/kg/day) |
| **Control** | Placebo (maltodextrin) |
| **N** | Study= 15, Control= 15 (crossover) |
| **Follow-up period** | 4 months each+1 month washout |
| **Outcomes assessed** | **Primary Outcome:** Functional benefit assessed by gait speed  **Secondary Outcomes:** 2-minute walk test, work, power, muscle mass (urinary creatinine), markers of myofibrillar protein breakdown (urinary 3-methyl-histidine/creatinine), serum creatine phosphokinase (CPK), body composition (fat free mass, fat mass percentage), safety and oral nutrient intake. |
| **Result (Primary outcome)** | No improvement in walking speed with glutamine versus placebo (median walking speed 0.9 m/s (±0.2) at baseline, stable to 0.8 m/s (±0.3) at 9 months; p=0.78). No significant improvement in 2-MWT, work, or power with glutamine compared to placebo. |
| **Result (Secondary outcome)** | Increase in fat free mass was significantly less with glutamine versus placebo, and an order effect was observed (p=0.001). In turn, percentage fat mass as measured by BIA was significantly greater with glutamine treatment. Fat mass percentage as measured by DXA was also significantly greater after glutamine (20.2% vs placebo 18.5%; p=0.03). No difference in muscle mass, markers of myofibrillar protein breakdown (3-methyl-histidine/creatinine ratio), or serum CPK between groups. |
| **Safety results** | No serious adverse effects observed. Adverse events reported included: gastroenteritis (n=1), urticaria (n=1), nervousness (n=1). |

| **Orndahl et al; 1984** | |
| --- | --- |
| **Country** | Sweden |
| **Study design** | Randomised controlled trial |
| **Age, years** | Mean (Range): Study- 43.6(35-62), Control- 47.4 (35-64) |
| **Disease** | Myotonic Dystrophy (MD) |
| **Inclusion Criteria** | 1)Well-established diagnosis of MD |
| **Exclusion Criteria** | - |
| **Patient Characteristics** | Function classes 1+2: Study (n=6), Control (n=7)  Function classes 3+4: Study (n=7), Control (n=7) |
| **Intervention** | Selenium and Vitamin E Supplements |
| **Intervention details** | 1st month- one tablet of 0.4 mg selenium and 200 mg vitamin E per day; After 1 month of treatment, the dose was raised to 2 tablets day and thereafter increased by 1 tablet at monthly intervals to a maximum of 1.6 mg selenium and 800 mg vitamin E per day. |
| **Control** | Placebo |
| **N** | Study= 13, Control= 14 |
| **Follow-up period** | 2 years |
| **Outcomes assessed** | **Primary Outcome**: Muscle strength (knee) by modified Cybex 11 isokinetic dynamometer, Handgrip strength by strain gauge dynamometer, Walking and stair-climbing tests.  **Secondary Outcomes:** Psychological tests- Mini Mental State Examination and Stanford Rating Scale (MMSE, SRB-3), Functional status questionnaire, |
| **Result (Primary outcome)** | Increase in knee-flexion strength (6.4 Nm) at 120" s (p < 0.05). Hand-grip strength and maximal walking speed decreased in both groups (p<0.01). |
| **Result (Secondary outcome)** | There were no significant changes in MMSE or SRB-3 or functional status over the treatment period. |
| **Safety results** | No serious adverse effects observed. A few patients contracted slight diarrhoea. |

| **Passerieux et al; 2013** | |
| --- | --- |
| **Country** | France |
| **Study design** | Randomised controlled trial |
| **Age, years** | Mean (SD): Study- 42.38(10.16), Control- 37.63 (9.27) |
| **Disease** | Facioscapulohumeral dystrophy (FSHD) |
| **Inclusion Criteria** | 1) between four and nine D4Z4 repeat units and positive family history for FSHD; 2) age between 18 and 60 years; 3) no HIV and/or hepatitis. |
| **Exclusion Criteria** | 1)confinement to a wheelchair, 2) smoking, 3) concomitant comorbidity (cardiac or pulmonary diseases, diabetes, etc.), or 4) being on medication (including mineral or vitamin supplement and /or other antioxidants). |
| **Patient Characteristics** | Steppage gait- 50% in study group vs 22% in placebo group |
| **Intervention** | Antioxidant Supplements |
| **Intervention details** | 500 mg vitamin C, 400 mg vitamin E, 25 mg zinc gluconate and 200μg selenomethionine once daily |
| **Control** | Placebo |
| **N** | Study= 26, Control= 27 |
| **Follow-up period** | 17 weeks |
| **Outcomes assessed** | **Primary Outcome:** 2 Minute Walk Test **(**2MWT), maximal voluntary contraction, and endurance limit time of the right and left quadriceps (MVCQR, MVCQL,TlimQR, and TlimQL) respectively using adapted exercise bench and strain gauge  **Secondary Outcomes:** changes in the antioxidant status and oxidative stress markers. |
| **Result (Primary outcome)** | MVCQ and TlimQ variations were significantly different between groups (MVCQD: p=0.011; MVCQND: p=0.004; TlimQD: p=0.028; TlimQND: p=0.011). 2MWT was significantly improved compared to baseline (p<0.001) but no significant difference between groups was observed. |
| **Result (Secondary outcome)** | Vitamin C (p=0.001), vitamin E as α-tocopherol (p=0.001), vitamin C/vitamin E ratio (p=0.017), vitamin E γ/α ratio (p=.022) and lipid peroxides (p=0.001) variations were significantly different between groups |
| **Safety results** | No adverse effects reported. |

| **Rodríguez-Cruz et al; 2018** | |
| --- | --- |
| **Country** | Mexico |
| **Study design** | Randomised controlled trial |
| **Age, years** | Mean (SD): Study- 7.28(3.08), Control- 8.56 (3.08) |
| **Disease** | Duchenne Muscular Dystrophy (DMD) |
| **Inclusion Criteria** | 1)Boys over the age of 3 and under 18 years 2) Deletion in the DMD gene or its promoter, as detected by Multiplex Polymerase Chain Reaction (MPCR) |
| **Exclusion Criteria** | 1)previously received glucocorticoids, consumed supplements containing omega-3 long-chain polyunsaturated fatty acids (PUFAs), or 2) had a known hypersensitivity to fish oil |
| **Patient Characteristics** | Wheelchair-bound- Study, n=2; Control, n=6 |
| **Intervention** | Omega-3 long chain-polyunsaturated fatty acid supplements |
| **Intervention details** | 10 identical capsules per day, providing a total of 2.9 grams per day of omega-3 long-chain polyunsaturated fatty acids (PUFAs), including 45 mg of eicosapentaenoic acid (EPA), 225 mg of docosahexaenoic acid (DHA), and 20 mg of additional omega-3 fatty acids per capsule. |
| **Control** | Placebo |
| **N** | Study= 18, Control= 22 |
| **Follow-up period** | 6 months |
| **Outcomes assessed** | **Primary Outcome:** mRNA expression by Reverse-Transcription quantitative PCR (Milliplex Map Immunoassay kit); and serum levels of Inflammatory markers- IL-6, lL-1b, TNFalpha, NF-kB IL-10; CK. |
| **Result (Primary outcome)** | mRNA of NK-kB and its target genes IL-1beta and IL-6 was downregulated significantly (p<0.05) in leukocytes compared to placebo group. Decrease in serum IL-1beta (-59.5%, p=0.011) and IL-6(-54.8%, p=0.041), increase in serum IL-10(99.9%, p<0.005). |
| **Result (Secondary outcome)** | - |
| **Safety results** | No adverse effects reported. |

| **Schneider-Gold et al; 2003** | |
| --- | --- |
| **Country** | Germany |
| **Study design** | Randomised controlled trial |
| **Age, years** | Mean (SD): 57.7 (8.7) |
| **Disease** | Myotonic dystrophy type 2 (DM2)/ Proximal myotonic myopathy (PROMM) |
| **Inclusion Criteria** | 1) Patients fulfilling the diagnostic criteria for PROMM |
| **Exclusion Criteria** | 1) Patients with renal or cardiac failure, 2) allergic reactions to food additives in their history, 3) diverticulitis, colitis, overt malignant diseases, and 4) Creatine medication during the last 4 months before the beginning of the study, 5) patients with DM1 mutation |
| **Patient Characteristics** | All patients had clinical and/or electric myotonia and proximal leg muscle weakness of Medical Research Council (MRC) grade 4 or less. |
| **Intervention** | Creatine Supplements |
| **Intervention details** | 10 g of Creapure per day (creatine monohydrate) |
| **Control** | Placebo- micro crystalline cellulose |
| **N** | Study= 10, Control= 10 |
| **Follow-up period** | 3 months |
| **Outcomes assessed** | **Primary Outcome:** Muscle strength using MRC scale and handheld dynamometry  **Secondary Outcomes:** Neuromuscular Symptom Score (NSS), maximum grip strength using grip balloon dynamometer, Visual analogue scale for activity |
| **Result (Primary outcome)** | No significant difference between creatine and placebo in MRC sum score (creatine: 139.5±7.4 to 142.6±5.2, change +3.2±4.4; placebo: 141.8±3.2 to 144.3±4.0, change +2.4±2.5) or hand-held dynamometry sum score (creatine: 163.3±24.6 to 170.9±35.0, change +7.6±20.0; placebo: 149.7±22.6 to 153.0±22.0, change +3.3±30.1). Both showed non-significant trends toward mild improvement with creatine |
| **Result (Secondary outcome)** | Visual analogue scale for daily activity showed greater absolute but not percentage change in creatine vs placebo (creatine +32.0±21.1 vs placebo +12.0±28.6; p=0.05). No improvement in NSS or maximum grip strength. |
| **Safety results** | No adverse effects reported. |

| **Sitzia et al; 2019** | |
| --- | --- |
| **Country** | Italy |
| **Study design** | Randomised controlled trial, phase II |
| **Age, years** | Mean (SD): 34.72 (15.49) |
| **Disease** | Muscular dystrophies- Duchenne (DMD), Facioscapulohumeral (FSHD), and Limb Girdle Muscular Dystrophy (LGMD) |
| **Inclusion Criteria** | 1) Documented genetic diagnosis and histological confirmation for inflammatory mononuclear cellular infiltrates in muscle biopsy; 2) age more than 9 in DMD, between 9-20 in LGMD, between 20-70 for FSHD |
| **Exclusion Criteria** | 1) severe cardiac or pulmonary disease, 2) corticosteroid treatment <6 months before study |
| **Patient Characteristics** | In each group DMD:FSHD:LGMD=2:1:1  Years since diagnosis [Mean(SD)]: DMD Study- 15 (2.76), Control- 16(3.79); Other Dystrophies Study-20 (11.80), Control- 17 (6.25)  All DMD and 5 LGMD patients were non-ambulant at baseline, all FSHD patients were ambulant. |
| **Intervention** | Flavonoids and Omega-3 Supplements |
| **Intervention details** | FLAVOMEGA: Dose: Sachet 1, powdered phase: 80 g net weight per day. Ingredients: fructose, phospholipidic curcumin, acetyl carnitine-L-HCl, ascorbic acid, flavoring, coenzyme Q10, skullcap (Scutellaria baicalensis Georgi) baicalin, green tea (Camellia sinensis) catechins, anti-agglomerant: silicon dioxide, sweeteners: acesulfame potassium and sucralose. Sachet 2, oily phase: 81.4 g net weight per day. Ingredients: fish oil 05/25, vitamin E acetate, citrus (Citrus limonum) essential oil. |
| **Control** | Placebo |
| **N** | Study= 15, Control= 14 |
| **Follow-up period** | 24 weeks |
| **Outcomes assessed** | **Primary Outcome:** Safety and efficacy by blood tests and side effects, global strength by Biodex System 4 Dynamometer functional test  **Secondary Outcomes:** 6 Minute Walk Test (6MWT). CK, Egen Klassifikation (EK) scale, Activity Limitation (ACTIVLIM) scale, Ability of Hand (ABILHAND manualability for adults with upper limb impairments scale) |
| **Result (Primary outcome)** | Significant treatment differences in isokinetic knee extension (P = 0.039) were observed in LGMD and FSHD subjects. |
| **Result (Secondary outcome)** | Significant change from baseline in 6min walk distance (P = 0.033) in LGMD and FSHD subjects. Serum Creatine Kinase (CK) levels decreased in all FLAVOMEGA treated groups with significant difference in DMD subjects (p=0.039). No significant difference in questionnaire data. |
| **Safety results** | No adverse effects reported. |

| **Tarnopolsky et al; 2004** | |
| --- | --- |
| **Country** | Canada |
| **Study design** | Randomised controlled crossover trial |
| **Age, years** | Mean (SD):41 (10) |
| **Disease** | Myotonic Dystrophy Type 1 (DM1) |
| **Inclusion Criteria** | 1) Adult (N 42) patients with genetically confirmed DM1 |
| **Exclusion Criteria** | 1) Taken Creatine monohydrate at least 3 months prior to starting the study. |
| **Patient Characteristics** | All patients were ambulatory and independent in their activities of daily living; none had functionally relevant cognitive impairment, were being treated for diabetes, or were participating in a formal exercise program |
| **Intervention** | Creatine Supplements |
| **Intervention details** | The CrM supplementation consisted of a flavored tablet containing lemon flavoring, dextrose (500 mg per tablet), and pure CrM (1 g/tablet, Neotine, Avicena Group, Palo Alto, California) at a total dose of 5 g CrM daily (0.074 0.02 g/kg daily). |
| **Control** | Placebo |
| **N** | Study = 21, Control= 21 (crossover) |
| **Follow-up period** | 4 months each + 6 weeks washout |
| **Outcomes assessed** | **Primary outcomes**: Anthropometry (height, weight, fat-free mass), Muscle PCr/B-ATP ratio using Magnetic Resonance Spectroscopy, Muscle strength using (Manual Muscle Testing) MMT  **Secondary Outcomes:** Isometric strength measurements (handgrip, foot dorsiflexion, knee extension); pulmonary function (FEV1, FVC); functional tasks (time to walk/wheel 30 feet, climb four stairs, stand from supine, cut a 3×3" square); activities of daily living (visual analogue scale); venous blood sampling (serum CK, AST, GGT, bilirubin, creatinine); 24-hour urine creatinine; muscle endurance (handgrip and foot dorsiflexion fatigue trials); side-effect questionnaire |
| **Result (Primary outcome)** | No significant differences in muscle strength (MMT mega-score: creatine 163.1±24.6 vs placebo 163.4±23.4), body composition, or bone density between CrM and placebo (all P>0.05). Muscle PCr/β-ATP ratios were similar following CrM (3.3±1.3) and placebo (3.2±1.3). |
| **Result (Secondary outcome)** | No significant differences in isometric strength (handgrip: creatine 20.2±14.0 vs placebo 20.6±14.2 N; dorsiflexion torque: 23.7±16.5 vs 23.7±17.6 Nm; knee extension: 101.3±48.0 vs 104.4±46.0 Nm), pulmonary function (FVC and FEV1 unchanged in both groups), functional tasks (stair climb, 30-foot walk, supine stand, cutting task), or ADL scale scores between conditions. Serum creatinine increased significantly following CrM (68 to 92 μmol/L) vs placebo (70 to 76 μmol/L; P<0.05), but creatinine clearance was unaffected. No differences in serum CK, liver function tests, or muscle endurance |
| **Safety results** | No adverse effects observed. |

| **Tarnopolsky et al; 2004** | |
| --- | --- |
| **Country** | Canada |
| **Study design** | Randomised controlled crossover trial |
| **Age, years** | Mean (SD): 10 (3) |
| **Disease** | Duchenne Muscular Dystrophy (DMD) |
| **Inclusion Criteria** | - |
| **Exclusion Criteria** | 1) Not taking creatine supplements |
| **Patient Characteristics** | 50% were taking corticosteroids |
| **Intervention** | Creatine Supplements |
| **Intervention details** | Creatine monohydrate in the form of a tablet containing lemon flavoring, dextrose (about 500 mg/tablet), and pure CrM (1 g/tablet, Neotine; Avicena Group, Palo Alto, CA). Each child received a total of 2 to 5 tablets/day (2 to 5g of CrM based on weight). |
| **Control** | Placebo |
| **N** | Study = 15, Control= 15 (crossover) |
| **Follow-up period** | 4 months each + 6 weeks washout |
| **Outcomes assessed** | **Primary outcomes**: Strength testing by Manual Muscle Testing (MMT) and maximal isometric handgrip strength, pulmonary function tests (functional vital capacity and forced expiratory volume in 1 second), body composition- fat free mass (FFM), percentage body fat, and bone mineral content by DEXA scan, functional tests (time to walk or wheel 30 ft, climb four standardized stairs, stand from a supine position, and cut a 3x3–in square from an 8x11–in piece of paper with scissors), Activity Scale for Kids questionnaire, venous blood sampling. |
| **Result (Primary outcome)** | Increase in handgrip strength in the dominant hand (p<0.05), and FFM (p<0.05), total MMT score decreased across time (collapsed across trial) by 6 months (p <0.05). Increase in FFM (0.7 kg; p<0.05) during the CrM treatment period, with no changes in bone density or content. CrM treatment resulted in a reduction of the N-telopeptide/creatinine content (p<,0.05). No significant effects on pulmonary function measures, functional tasks, or activities of daily living scales. |
| **Result (Secondary outcome)** | - |
| **Safety results** | No adverse effects observed. |

| **Walter et al; 2000** | |
| --- | --- |
| **Country** | Germany |
| **Study design** | Randomised controlled crossover trial |
| **Age, years** | Mean (SD): 10(4) |
| **Disease** | Muscular dystrophies (MD) |
| **Inclusion Criteria** | 1) Clinically, morphologically, and genetically defined MD |
| **Exclusion Criteria** | 1) Patients confined to bed or wheelchair, 2) on corticosteroid medication or 3) with renal insufficiency. |
| **Patient Characteristics** | 12 patients with facioscapulohumeral dystrophy, 10 patients with Becker dystrophy, 8 patients with Duchenne dystrophy, and 6 patients with sarcoglycan-deficient limb girdle muscular dystrophy |
| **Intervention** | Creatine Supplements |
| **Intervention details** | CREAPURE (SKW, Trostberg, Germany): adults, 10 g/day; children, 5 g/day |
| **Control** | Placebo |
| **N** | Study = 17, Control= 15 (crossover) |
| **Follow-up period** | 8 weeks each + 3 weeks washout |
| **Outcomes assessed** | **Primary outcomes**: Muscle strength (MRC scale), daily-life activities (NSS), vital capacity (VC), patient's own assessment of improvement, laboratory tests (blood chemistry, CK levels) |
| **Result (Primary outcome)** | Significant mean improvement of 3% in muscle strength during creatine vs placebo (MRC difference verum: +2.5±5.7, 95% CI −0.4 to 5.4; placebo: −3.2±4.7, 95% CI −5.8 to −0.5; p<0.05). Significant improvement of 10% in daily-life activities (NSS difference verum: +2.1±3.9, 95% CI 0.1 to 4.1; placebo: −0.9±5.0, 95% CI −3.7 to 1.8; p<0.05). 60% of patients assigned improvement to creatine (p<0.05). No significant changes in vital capacity or laboratory tests. |
| **Result (Secondary outcome)** | - |
| **Safety results** | No adverse effects observed. |

| **Walter et al; 2002** | |
| --- | --- |
| **Country** | Germany |
| **Study design** | Randomised controlled crossover trial |
| **Age, years** | Mean (SD):44 (13) |
| **Disease** | Myotonic Dystrophy Type 1 (DM1) |
| **Inclusion Criteria** | 1) Clinically, electrophysiologically and genetically defined DM1 |
| **Exclusion Criteria** | 1) Patients confined to bed or wheelchair or 2) with renal insufficiency. |
| **Patient Characteristics** |  |
| **Intervention** | Creatine Supplements |
| **Intervention details** | CREAPURE®,Degussa AG,Trostberg, Germany : 10.6g/day 1–10,5.3 g/day 11–56 |
| **Control** | Placebo- microcrystalline cellulose |
| **N** | Study = 17, Control= 17 (crossover) |
| **Follow-up period** | 8 weeks each + 6 weeks washout |
| **Outcomes assessed** | **Primary outcomes**: Muscle strength- MRC scale, Quantitative muscle strength using Multi Muscle Tester M3 Diagnos System, Neuromuscular Symptom and Disability Functional Score (NSS), body cell mass (BCM), lean body mass (LBM), fat mass, total body water (TBW), and intracellular water (ICW) using bioelectrical impedance analysis, pulmonary function (forced vital capacity, maximum inspiratory pressure), Patients global assessment of improvement |
| **Result (Primary outcome)** | No significant difference in MRC (p=0.1127) or NSS (p=0.1507) in overall crossover analysis. In second crossover period only, creatine improved while placebo worsened for MRC (p=0.0049) and NSS (p=0.0636). No significant differences in QMA biceps (p=0.0668), QMA quadriceps (p=0.4500), lean body mass (p=0.6882), body weight (p=0.9544), or vital capacity (p=0.2446). 73% reported no difference; 18% attributed improvement to creatine; 9% to placebo. |
| **Result (Secondary outcome)** | - |
| **Safety results** | No adverse effects observed. |

| **Wilson et al; 2024** | |
| --- | --- |
| **Country** | France |
| **Study design** | Randomised controlled trial |
| **Age, years** | Mean (SD): Study- 39.6(11.6), Control- 42.1(9.8) |
| **Disease** | Facioscapulohumeral muscular dystrophy (FSHD) |
| **Inclusion Criteria** | 1) Adults age 18–60 years 2) Genetic diagnosis of FSHD1 (D4Z4 repeat units 4–9) 3) Family history of FSHD |
| **Exclusion Criteria** | 1)Confinement to a wheelchair, 2) smoking, 3) concomitant comorbidity (e.g. cardiopulmonary diseases, diabetes), or 4) current treatment (including mineral or vitamin supplement and/or other antioxidants). Exclusion criteria for the MRI evaluation: patients with claustrophobia, cardiac pacemaker or defibrillator, cochlear, otologic or ear implant, and metal foreign intraocular bodies. |
| **Patient Characteristics** | - |
| **Intervention** | Antioxidant Supplements |
| **Intervention details** | Antioxidant supplement: Vitamin C 500 mg, Vitamin E 400 mg, Zinc gluconate 25 mg, Selenomethionine 200 µg daily |
| **Control** | Placebo |
| **N** | Study = 10, Control= 10 |
| **Follow-up period** | 17 weeks |
| **Outcomes assessed** | **Primary outcomes**: Muscle strength of both quadriceps by Maximal voluntary contraction of quadriceps muscle (MVCQ) using adapted exercise bench  **Secondary outcomes:** Muscle composition parameters measured by MRI (muscle volume, fat volume, muscle quality ratios), oxidative stress and antioxidant markers (lipid peroxides, oxidized DNA, vitamins C and E, selenium, zinc, copper, GSH/GSSG ratio, GPx, SOD1), and quality of life assessed by SF-36 questionnaire. |
| **Result (Primary outcome)** | Significant improvement in muscle strength in both dominant and non-dominant quadriceps (MVCQD: +18.2%, p=0.014; MVCQND: +31.1%, p=0.042) with significant between-group differences (p=0.029 and p=0.031). |
| **Result (Secondary outcome)** | Significant improvements in muscle quality (p=0.020), muscle and fat volumes in non-dominant quadriceps (p=0.003), physical functioning on SF-36 (p=0.045), and antioxidant markers (p<0.05). |
| **Safety results** | No adverse effects observed. |

**Distal Myopathy**

| **Argov et al; 2016** | |
| --- | --- |
| **Country** | United States and Israel |
| **Study design** | Randomised controlled trial |
| **Age, years** | Mean (SD): Study- 6g/day- 42.8 (8.86); 3g/day- 40.7 (12.71); Placebo- 35.3 (7.39) |
| **Disease** | GNE Myopathy |
| **Inclusion Criteria** | 1) Documented diagnosis of GNE myopathy, Heriditary Inclusion Body Myositis (HIBM), distal myopathy with rimmed vacuoles (DMRV), or Nonaka disease due to previously demonstrated mutations in the gene encoding the GNE/MNK enzyme, 2) Able to walk 20 meters independently (may use orthotics and assistive devices), 3) Able to provide reproducible force in bilateral elbow flexors and knee extensors during hand-held dynamometry testing (unilateral between test variability of < 15% for both muscle groups) |
| **Exclusion Criteria** | 1) Pregnant or breastfeeding at Screening or planning to become pregnant at any time during the study, 2) Ingestion of N-acetyl-D-mannosamine (ManNAc), Sialic Acid (SA) , or related metabolites; intravenous immune globulin (IVIG); or anything that can be metabolized to produce SA in the body for the prior 60 days, 3) hypersensitivity to SA or its excipients, 4) Elevated serum transaminases levels three times the upper limit of normal or serum creatinine of greater than 2.0 mg/dL |
| **Patient Characteristics** | 1) Age at symptom onset 27.6 (7.79); 2) Orthotic use in 60% (n= 28); 3) Assistive device use in 50%: Wheelchair use (n=11), Walking device use (n=24) |
| **Intervention** | Aceneuramic acid |
| **Intervention details** | Divided into three arms: aceneuramic acid 3g/day (n= 18) and 6g/day (n=15); and placebo (n=14) for 24 weeks. Cross-over of placebo group into 3g/day (n=23) and 6g/day (n=24) for additional 24 weeks |
| **Control** | Placebo |
| **N** | First 24 weeks: 3g/day (n= 18) and 6g/day (n=15); and placebo (n=14); Second 24 weeks: 3g/day (n=23) and 6g/day (n=24) |
| **Follow-up period** | 48 weeks |
| **Outcomes assessed** | **Primary Outcome**: Serum SA, muscle strength by dynamometry, functional assessments including 6 Minute Walk Test (6MWT) and gait speed ([GS] test, timed stair climb [SC] test, modified 30-second sit to-stand [STS] test) and 1-kg weighted arm lift [WAL]test, clinician- and patient-reported outcomes (GNEM-Functional Activity Scale, Individual Neuromuscular Quality of Life (INQoL), Inclusion Body Myositis Functional Rating Scale (IBMFRS)),  Muscle MRI,  Electrical impedance myography (EIM) and safety |
| **Result (Primary outcome)** | Supplementation with Ace-ER resulted in maintenance of muscle strength in an upper extremity composite (UEC) score at 6g/day compared with placebo at Week 24 (LS mean difference +2.33 kg (95% CI 0.11, 4.55), p=0.040), and larger in a pre-specified subgroup able to walk ≥200 m (+3.10 kg (95% CI 0.14, 6.06), p=0.040). After cross-over, a combined 6g/day group showed significantly better UEC strength than a combined 3g/day group (+3.46 kg (95% CI 1.17, 5.75), p=0.0031), with a larger effect in the ≥200m subgroup (+4.69 kg (95% CI 2.06, 7.33), p=0.0005). A similar dose-dependent response was observed in the lower extremity composite (LEC) score, but it was not significant at Week 24 (+1.06 kg (95% CI −3.04, 5.17), p=0.61) or Week 48 (+1.31 kg (95% CI −2.49, 5.10), p=0.50). In the ≥200m subgroup, quadriceps strength showed a significant difference at Week 24 (+3.31 kg (95% CI 0.06, 6.56), p=0.046). No statistically significant changes were observed in walking-related tests at Weeks 24 or 48 (6MWT p=0.95; comfortable gait speed p=0.90; stair climb p=0.25; sit-to-stand p=0.28; weighted arm lift p=0.80). The GNEM-FAS demonstrated a trend improvement in upper extremity function and mobility in the combined 6g/day group compared with the combined 3g/day group. IBMFRS and INQoL showed dose-dependent positive statistical trends at Week 48. No change in MRI scores from Baseline to Week 24; MRI not conducted at Week 48. EIM showed statistically significant dose-dependent changes in impedance patterns at Week 24 in the 6g/day group. |
| **Result (Secondary outcome)** | - |
| **Safety results** | No adverse effects observed. |

| **Mori-Yoshimura et al; 2023** | | |
| --- | --- | --- |
| **Country** | Japan |  |
| **Study design** | Randomised controlled trial |  |
| **Age, years** | Mean (SD): Study- 41.3 (6.1), controls- 37.0 (6.9) |  |
| **Disease** | GNE Myopathy |  |
| **Inclusion Criteria** | 1)Genetically confirmed GNE gene mutations 2) Two dynamometry force values <15% variability in the dominant arm 3) Disease duration of 5-15 years 4) GNEM-FAS (GNE myopathy-Functional Activity Scale) score of 24 points or more in the upper extremity |  |
| **Exclusion Criteria** | Use of substances that can be metabolised to sialic acid <60 days of study; Liver function tests >3x ULN; Serum creatine >2x ULN |  |
| **Patient Characteristics** | Age at onset of symptoms, years [Mean(SD)]: Study- 31.4 (6.0); Control- 27.5 (5.1) |  |
| **Intervention** | Aceneuramic Acid (sialic acid-extended release (SA-ER)) |  |
| **Intervention details** | SA-ER tablet-4 tablets of 500 mg of aceneuramic acid three times per day |  |
| **Control** | Placebo |  |
| **N** | Study= 10, Controls= 4 |  |
| **Follow-up period** | 48 weeks |  |
| **Outcomes assessed** | **Primary Outcome**: Change in upper extremity composite (UEC) score from baseline to week 48  **Secondary Outcomes**: Investigator-assessed efficacy based on Manual muscle testing (MMT) for upper extremity or grip strength, UEC score and difference in UEC score based on other similar studies |  |
| **Result (Primary outcome)** | Decrease in least square mean (LSM) change in UEC score at Week 48 with SA-ER (−0.115 kg) was numerically smaller as compared with placebo (−2.625 kg), with LSM difference (95% confidence interval) of 2.510 (−1.720 to 6.740) kg. |  |
| **Result (Secondary outcome)** | SA-ER group had higher efficacy (70%, 95% CI 34.75–93.33) when compared with placebo  (50%, 95% CI 6.76–93.24) |  |
| **Safety results** | No adverse effects observed. |  |

| **Park et al; 2023** | | |
| --- | --- | --- |
| **Country** | Republic of Korea |  |
| **Study design** | Randomised controlled trial |  |
| **Age, years** | Mean (SD): 3g/day- 34.5 (5); 6g/day- 36.2 (4.5) |  |
| **Disease** | GNE Myopathy |  |
| **Inclusion Criteria** | Clinical and genetic diagnosis of GNE myopathy |  |
| **Exclusion Criteria** | 1)Pregnancy 2) Age <18years and >70 years 3) Abnormal liver or kidney function |  |
| **Patient Characteristics** | Age at onset of symptoms, years [Mean(SD)]: 3g/day- 8.6 (5.6); 6g/day- 8.7 (3.8) |  |
| **Intervention** | 6′-sialyllactose (6SL) |  |
| **Intervention details** | 6g per day 6′-sialyllactose |  |
| **Control** | 3g per day |  |
| **N** | Study = 7, Control= 7 |  |
| **Follow-up period** | 96 weeks |  |
| **Outcomes assessed** | **Primary Outcome**: Motor function by handheld dynamometers combined by upper extremity and lower extremity composite score (UEC and LEC), 6 Minute Walk Test (6MWT)  Secondary Outcomes: Fat fraction on muscle MRI; GNE myopathy functional activity scale (GNEM-FAS) |  |
| **Result (Primary outcome)** | In the high-dose group (6g/day), motor powers of shoulder abduction (p=0.0113), elbow flexion (p=0.0153), hip flexion (p=0.0487), and knee extension (p<0.0001) showed a significant incremental tendency over 96 weeks by simple linear regression. Shoulder abduction, elbow flexion, hip flexion, and ankle dorsiflexion were significantly superior in the high-dose group compared with the low-dose group (two-way ANOVA; p<0.05, p=0.038, p=0.0015, and p=0.0021, respectively). The lower extremity composite (LEC) score tended to improve in both groups (simple linear regression, p=0.0458), with the increase more prominent and statistically significant in the high-dose group (two-way ANOVA, p=0.0406). The upper extremity composite (UEC) score failed to show an incremental tendency in either group. 6MWT remained stable in both groups |  |
| **Result (Secondary outcome)** | Fat fraction on muscle MRI increased in the low-dose group in all compartments (simple linear regression: anterior thigh p=0.0002, posterior thigh p=0.0484, anterior lower leg p=0.0338, posterior lower leg p=0.0280). The increase was attenuated in the high-dose group in all four compartments (two-way ANOVA: anterior thigh p=0.0005, posterior thigh p=0.0218, anterior lower leg p=0.0042, posterior lower leg p=0.003). GNEM-FAS total and domain scores consistently decreased over the study period, with no significant difference between the two groups |  |
| **Safety results** | No adverse effects observed. |  |

| **Suzuki et al; 2023** | | |
| --- | --- | --- |
| **Country** | Japan |  |
| **Study design** | Randomised controlled trial |  |
| **Age, years** | Mean (SD): Study- 47 (7) years; Control- 37 (1) |  |
| **Disease** | GNE Myopathy |  |
| **Inclusion Criteria** | 1)Confirmed GNE myopathy 2) 20-50 years |  |
| **Exclusion Criteria** | 1)Sialic acid like supplements including IVIG <60days of study 2)Sialic acid <30days of study 3) Pregnant/lactating |  |
| **Patient Characteristics** | Age at onset of symptoms, years [Mean(SD)]: Study- 30 (7); Control- 27 (1) |  |
| **Intervention** | Aceneuramic acid/sialic acid-extended release (SA-ER) |  |
| **Intervention details** | 4 tablets of 500mg thrice daily = 6g/day |  |
| **Control** | Placebo |  |
| **N** | Study= 15, Control= 4 |  |
| **Follow-up period** | 48 weeks |  |
| **Outcomes assessed** | **Primary Outcome**: Upper extremity composite (UEC) score by hand held dynamometer  **Secondary Outcomes**: GNEM-Functional Activity Scale (GNEM FAS) in upper extremity and mobility domain, Lower extremity composite (LEC) score, 6 Minute Walk Test (6MWT), sit-to-stand test score (30-second), Number of lifts in the weighted arm lift test (30-second), muscle strength and its percentage relative to the predicted standard value of knee extensors |  |
| **Result (Primary outcome)** | The mean value of change in UEC score (95% CI) at 48 weeks was −0.1 kg (−2.1 to 2.0) in the SA-ER group and −5.1 kg (−10.4 to 0.3) in the placebo group. Covariance analysis revealed least squares mean difference of 4.8 kg (95% CI −0.3 to 9.9; p=0.0635) between the two groups. GEE repeated measures analysis showed estimated values of change (95% CI) at 48 weeks of −1.0 kg (−2.5 to 0.5) in the SA-ER group and −5.7 kg (−8.0 to −3.4) in the placebo group, with a significant difference of 4.7 kg (95% CI 1.8 to 7.5; p=0.0013) between the two groups. |  |
| **Result (Secondary outcome)** | Mean changes (95% CI) at final evaluation in the SA-ER vs placebo groups were: LEC +2.1 kg (−3.2 to 7.4) vs −3.8 kg (−10.9 to 3.3); sit-to-stand test +1.9 (0.7 to 3.0) vs +1.5 (−1.5 to 4.5); weighted arm lift test +2.1 (−1.8 to 6.1) vs +1.5 (−12.8 to 15.8); knee extensors +1.2 kg (−1.3 to 3.7) vs −1.9 kg (−9.5 to 5.7); 6MWT −19.3 m (−36.1 to −2.5) vs +4.5 m (−32.9 to 42.0); GNEM-FAS mobility domain −0.6 (−2.2 to 1.0) vs +0.5 (−2.3 to 3.3); GNEM-FAS upper extremity domain +0.9 (−0.7 to 2.4) vs −0.8 (−3.5 to 2.0). |  |
| **Safety results** | No serious related adverse effects. |  |

**Metabolic and Mitochondrial Myopathies**

| **Andersen et al; 2008** | |
| --- | --- |
| **Country** | Denmark |
| **Study design** | Randomised, open-labeled, crossover study |
| **Age, years** | Mean (SD): Study- 38 (5), Control- 38 (5) |
| **Disease** | McArdle Disease |
| **Inclusion Criteria** | 1)Diagnosis was confirmed by genetic and biochemical testing in all patients, 2) Life long exercise intolerance 3) life-long exercise intolerance 4) Repeated exercise-induced episodes of muscle cramps |
| **Exclusion Criteria** | - |
| **Patient Characteristics** | 1) Five of the patients had experienced episodes of  Myoglobinuria, 2) One patient had diabetes mellitus type 2, treated with glimepiride (6 mg daily) |
| **Intervention** | Carbohydrate or Protein rich diet |
| **Intervention details** | 1) Protein-rich diet: containing 15% fat, 55% protein and 30% carbohydrate (chicken, beef, ham, eggs, nuts, bread, pasta, potato and cheese), 2) Carbohydrate-rich diet: containing 20% fat, 15% protein and 65% carbohydrate (vegetarian food: vegetables, fruits, pasta, rice, bread and low-fat cheese) |
| **Control** | Carbohydrate-rich diet or Protein rich diet alternative weeks |
| **N** | Study= 4, Control= 3 |
| **Follow-up period** | 3 days of diet, one week washout |
| **Outcomes assessed** | **Primary Outcome**: Constant workload bicycle ergometer for Heart rate and Visual analogue scale (Borg scale) for perceived exertion  **Secondary Outcomes**: Venous blood samples for plasma glucose, lactate and insulin levels |
| **Result (Primary outcome)** | Heart rate and perceived exertion were consistently lower at the constant workload on the carbohydrate versus the protein-rich diet (p<0.0005); a second wind phenomenon occurred in both groups but heart rate and perceived exertion remained lower in the carbohydrate group. |
| **Result (Secondary outcome)** | No change in glucose concentrations across both the groups. Serum lactate levels higher in carbohydrate groups during rest and activity (p= 0.01). Insulin concentrations were higher in the carbohydrate-rich trial, and decreased during exercise on both treatments. |
| **Safety results** | No adverse effects reported. |

| **Bleeker et al; 2020** | |
| --- | --- |
| **Country** | Netherlands |
| **Study design** | Randomised controlled trial |
| **Age, years** | Median: 22 years |
| **Disease** | Very Long-Chain acyl-CoA Dehydrogenase (VLCAD) deficiency |
| **Inclusion Criteria** | 1) Confirmed VLCADD by genetic profiling and 2) Normal cardiopulmonary function |
| **Exclusion Criteria** | 1)Recent episode of rhabdomyolysis or acute renal failure in the past 2 months; 2) inability to perform bicycle test; 3) known coronary artery disease, positive history for angina, or changes on ECG suggestive of previous ischaemia without a negative stress test; 4) insulin-dependent diabetes mellitus. |
| **Patient Characteristics** | Age at onset of symptoms, years [Mean(SD)]: Study- 4.5 (2.1), Control 4.4 (1.7) |
| **Intervention** | Nutritional ketosis induced by Ketone+carbohydrate drink |
| **Intervention details** | 395 mg/kg of ketone ester + 54 g of dextrose |
| **Control** | Carbohydrate drink (dextrose) |
| **N** | Study= 2, Control= 3 |
| **Follow-up period** | 7 days |
| **Outcomes assessed** | **Primary Outcome:** Quadriceps phosphocreatine (PCr), inorganic phosphate (Pi) and pH dynamics during exercise and recovery assayed by in vivo 31 P-MR spectroscopy.  **Secondary Outcomes:** Whole blood beta hydroxy butyrate (BHB) and Glucose levels, plasma lactate, acylcarnitine levels, Muscle glycolysis and fatty oxidation. |
| **Result (Primary outcome)** | Quantitative analysis revealed 3, 4, and 5 patients with 40% reduction in the (inorganic phosphate to phosphocreatine ratio (Pi/PCr) in exercising leg muscle than in the CHO arm. No changes in patient 1. Measured data lost in patient 2. Post-exercise, the rate of oxidative metabolic recovery indexed by the time constant of PCr recovery to basal level was identical for both arms. |
| **Result (Secondary outcome)** | Blood BHB increased to 2.0±0.2 mmol/L within 30 minutes of KE ingestion, remaining significantly higher than CHO for the entire protocol (p=0.0002 for intervention effect; p<0.0001 for time and intervention×time). Blood glucose remained within normal range in both arms (KE 5.7±0.4 vs CHO 6.4±0.7 mmol/L). Plasma lactate tended to be 1.3-fold lower at end of exercise in KE+CHO vs CHO (p=0.07). NK reduced total long-chain acylcarnitines including VLCADD-specific C14:1-carnitine during exercise. Intramuscular BHB was taken up by muscle during NK; sum of intramuscular glycolytic intermediates remained unchanged in NK but increased in 3/4 patients after CHO ingestion. No significant differential effect on intramuscular fat oxidation. |
| **Safety results** | No adverse effects reported. |

| **Chen et al; 1997** | |
| --- | --- |
| **Country** | Taiwan |
| **Study design** | Randomised controlled trial (crossover) |
| **Age, years** | Mean (SD): Study- 38.8 (15.3) |
| **Disease** | Mitochondrial Encephalomyopathies |
| **Inclusion Criteria** | 1) Monoclonal epilepsy with red ragged fibers(MERRF) syndrome (n=4); 2) Mitochondrial myopathy, encephalopathy, lactic acidosis and stroke-like episodes syndrome (MELAS) (n=3); 3) Chronic progressive external ophthalmoplegia with myopathy (CPEOM) (n=1) |
| **Exclusion Criteria** | None |
| **Patient Characteristics** | Age at onset of symptoms, years [Mean(SD)]: Study- 23.8 (7.8) |
| **Intervention** | Coenzyme Q10 (CoQ10) |
| **Intervention details** | CoQ10 160mg/day orally for three months |
| **Control** | Placebo |
| **N** | Study= 8, Control= 8 |
| **Follow-up period** | 3 months |
| **Outcomes assessed** | **Primary Outcome**: Global muscle strength by Medical Research Council scale (MRC), Subjective improvement in activities of daily living (ADL)  **Secondary Outcomes**: Serum CoQ10 levels, exercise-induced lactate and pyruvate levels (via modified Bruce treadmill protocol). |
| **Result (Primary outcome)** | Statistically significant improvement in the global MRC (Medical Research Council) index score after CoQ10 supplementation (p<0.05); while a trend of improvement in ADL scores was noted during CoQ10 treatment and relative worsening during placebo, these changes did not reach statistical significance. |
| **Result (Secondary outcome)** | Serum CoQ10 levels increased significantly after 2 and 3 months of treatment (p < 0.05), rising from a baseline of 0.73 ± 0.13 µg/ml to 2.91 ± 2.02 µg/ml. However, there was no significant difference in the metabolism of lactate or pyruvate during exercise before vs. after therapy. |
| **Safety results** | No adverse effects reported. |

| **Gillingham et al; 2019** | |
| --- | --- |
| **Country** | United States of America |
| **Study design** | Randomised controlled trial |
| **Age, years** | Mean (SD): Study**-** 14.3 (5.12) |
| **Disease** | Long-chain fatty acid β-oxidation disorders (LC-FAODs) |
| **Inclusion Criteria** | 1) Confirmed diagnosis of Trifunctional Protein(TFP), Long Chain Hydroxyacyl CoA Dehydrogenase (LCHAD), Carnitine Palmitoyltransferase (CPT2) or Very Long Chain Acyl CoA Dehydrogenase (VLCAD) deficiency 2)at least 7 years of age |
| **Exclusion Criteria** | 1)Diabetes, thyroid disease or other endocrine dysfunction that alters body composition. 2) Pregnancy 3) Anemia |
| **Patient Characteristics** | 1 adolescent-onset LCHAD deficiency, 9 infant-onset LCHAD deficiency |
| **Intervention** | High protein diet (Hi PRO) |
| **Intervention details** | High protein diet + whey protein supplement (25% of daily caloric demand) |
| **Control** | High carbohydrate diet (Hi CHO) |
| **N** | Study= 7, Control= 6 |
| **Follow-up period** | 4 months |
| **Outcomes assessed** | **Primary Outcome**: Dual Energy X ray Absorptiometry (DEXA) for Lean body mass, Muscle and liver lipid deposition by Magnetic resonance Spectroscopy  **Secondary Outcomes**: Resting energy expenditure by Indirect calorimeter, Total energy expenditure (TEE) using double labeled water (DLW), serum glucose, insulin and free fatty acids |
| **Result (Primary outcome)** | The PRO group maintained lean body mass while the CHO group experienced a significant loss (P = 0.02). The decrease in lean mass observed in the Hi CHO group occurred predominately among subjects who were greater than 16 years old (Pearson's R2 = 0.65; P = 0.053 slope different from 0 for the Hi CHO group; R2 = 0.11; P = 0.46 for Hi PRO group). Reduced intrahepatic lipid content was noted in the PRO group (P = 0.08). No difference was observed in muscle lipid deposition. |
| **Result (Secondary outcome)** | Resting energy expenditure was not significantly different between subjects on the Hi PRO compared to the Hi CHO diet; No difference in TEE between the diet-assignment groups (unpaired t test P = 0.64); While there was no difference between the two groups, the Hi CHO group's measured TEE was significantly lower than their expected energy requirement (P = 0.03), whereas the Hi PRO group's TEE was similar to their expected requirement. Fasting FFA, glucose and insulin levels were similar between the groups. |
| **Safety results** | No adverse effects observed. |

| **Gimenes et al; 2015** | |
| --- | --- |
| **Country** | Brazil |
| **Study design** | Randomised controlled trial |
| **Age, years** | Mean (SD): Study- 35.4±10.8; Control- 29±7.8 |
| **Disease** | Mitochondrial myopathy (MM) |
| **Inclusion Criteria** | 1) MM with chronic progressive external ophthalmoplegia (CPEO); 2) complaint of exercise intolerance, mild fixed limb weakness, or myalgias |
| **Exclusion Criteria** | 1) cardiac pacemaker; 2) severe limb weakness; 3) concomitant ergogenic supplementation treatment; 4) renal, cardiac, or pulmonary dysfunction; or 5) participation in regular physical activity in the preceding year |
| **Patient Characteristics** | Age at onset of symptoms, years [Mean(SD)]: Study- 34.5 (11.2) |
| **Intervention** | L-carnitine |
| **Intervention details** | 3gms L-carnitine once daily |
| **Control** | Placebo 3gms |
| **N** | Study= 6, Control= 6 |
| **Follow-up period** | 16 weeks |
| **Outcomes assessed** | Primary Outcome: Incremental cardiopulmonary exercise tests (ICPET) by cycle ergometer, constant work rate exercise test (CWR) by cycle ergometer, and isokinetic muscle endurance by dynamometer.  Secondary Outcomes: Body composition using skin fold caliper and mid-arm circumference (MUAC), pulmonary function tests using spirometry. |
| **Result (Primary outcome)** | No significant differences were detected in metabolic, ventilatory, or cardiovascular variables in the study group on ICPET (1216.7 ± 402 vs 1193 ± 371 mL/min, P > 0.05); CWR exercise testing observed a significantly longer limit of tolerance (Tlim) after L-carnitine (14 ± 1.9 vs 11 ± 1.4 min, P < 0.05); higher oxygen consumption (VO2) was observed at the 6th min of exercise (isotime) and at Tlim (1223 ± 114 vs 1060 ± 108 mL/min, P < 0.05); the gas-exchange ratio was significantly lower at isotime after L-carnitine compared to placebo (1.03 ± 0.04 vs 1.11 ± 0.04, P < 0.05); there were no significant changes in peripheral muscle strength, including peak torque (95.4 ± 12.1 vs 93.4 ± 35.5 N/m, P > 0.05), between the L-carnitine and placebo arms. |
| **Result (Secondary outcome)** | No change was observed in body mass index (20.4 ± 1.1 vs 20.6 ± 4.3 kg/m2, P > 0.05) or arm muscle circumference in either the study or placebo arms; regarding pulmonary function, only the inspiratory capacity (IC) significantly increased in the study group compared to placebo (2.45 ± 0.23 vs 2.26 ± 0.22 L, P < 0.05), while other volumes like residual volume remained unchanged. |
| **Safety results** | No adverse effects observed. |

| **Glover et al; 2010** | |
| --- | --- |
| **Country** | Canada |
| **Study design** | Randomised controlled trial |
| **Age, years** | Mean (SD): Study- 48 +/- 3; Control- 56 +/- 3 |
| **Disease** | Mitochondrial myopathies and cytopathies |
| **Inclusion Criteria** | Diagnosis confirmed based on clinical symptoms, fasting serum lactate concentration, muscle biopsy findings (ragged red fibers and/or cytochrome c oxidase–negative fibers), and mitochondrial DNA analysis |
| **Exclusion Criteria** | None |
| **Patient Characteristics** | - |
| **Intervention** | Coenzyme Q10 |
| **Intervention details** | Coenzyme Q10 600 mg twice daily |
| **Control** | Placebo (Soybean oil) |
| **N** | Study= 15, Control= 15 |
| **Follow-up period** | 60 days |
| **Outcomes assessed** | **Primary Outcome:** Bone mineral density and lean body mass by DEXA, forearm fatigue testing with near-infrared spectroscopy (NIRS), cycle ergometry, Activities of daily living (ADL)  **Secondary Outcomes:** Brain magnetic resonance spectroscopy (MRS) for lactate, absolute creatine and choline containing compounds, urinary markers of oxidative stress (8-OHdG and 8-IsoP) |
| **Result (Primary outcome)** | Significant increase in Oxygen consumption (VO2) (per kg lean mass) [CoQ10: 19.3 (1.3); Placebo: 17.4 (1.3); p=0.046] with CoQ10 treatment observed; No differences in bone mineral density (p=0.76) and lean body mass (p=0.91); Maximal isometric forearm strength was not significantly different between treatment phases [placebo: 24.6 (1.6); CoQ10: 23.8 (1.8); p=0.27] on NIRS; On cycle ergometer, no differences between treatments after 15 minutes of ride for heart rate (p=0.45), respiratory exchange ratio (p=0.72), and expired volume per minute (p=0.30).The mean scores for Activities of Daily Life (ADL) were not different among the treatment groups (p=0.26) and Quality of Life (QOL) was not different (p=0.09). |
| **Result (Secondary outcome)** | Coenzyme Q10 treatment attenuated the rise in lactate after cycle ergometry at +1 minute (p=0.002), but did not significantly increase VO2/kg lean mass after only five minutes of cycling (p=0.43); treatment resulted in decreased gray matter choline-containing compounds [CoQ10: 0.166 (0.017); Placebo: 0.217 (0.026); p=0.038]; no significant differences were found for urinary 8-OHdG (p=0.38) or 8-IsoP (p=0.81). |
| **Safety results** | No adverse effects observed with CoQ10 with doses as high as 3000-3600mg/day |

| **Hoogeveen et al; 2021** | |
| --- | --- |
| **Country** | Netherlands |
| **Study design** | Randomised controlled trial (crossover) |
| **Age, years** | Median: 46 years |
| **Disease** | Glycogen storage disease type IIIa (GSDIIIa) |
| **Inclusion Criteria** | 1) confirmation of GSDIII with enzyme assay and/or Glucose Debranching Enzyme (GDE) variation analysis; 2) GSDIIIa further specified as GDE deficiency in muscle or clinical and/or biochemical signs of cardiac and/or skeletal muscle involvement |
| **Exclusion Criteria** | 1) pregnancy or breastfeeding, 2) insulin-dependent diabetes mellitus, 3) recent cardiac disease (including cardiomyopathy, coronary artery disease, or a positive history for angina pectoris), 4) contraindications for magnetic resonance imaging studies, 5) unable to perform bicycle exercise |
| **Patient Characteristics** | - |
| **Intervention** | Acute Nutritional ketosis with ketone drink |
| **Intervention details** | Ketone-ester (395 mg/kg, KE) + carbohydrate drink (30 g, KE + CHO) |
| **Control** | Carbohydrate drink (~66g, CHO) |
| **N** | Study= 6, Control= 6 |
| **Follow-up period** | 7 days |
| **Outcomes assessed** | **Primary Outcome:** Blood beta-hydroxybutyrate (βHB) and glucose concentrations, exercise performance assessed by indirect calorimetry and heart rate measurements, and in vivo ³¹P magnetic resonance (³¹P-MR) spectra of quadriceps muscle during exercise and recovery including inorganic phosphate (Pi), phosphocreatine (PCr), and intramuscular pH.  **Secondary Outcomes:** Blood concentrations of acetoacetate (AcAc), insulin, lactate, and free fatty acids (FFAs), rate of perceived exertion (RPE) scores, and urinary excretion of beta-hydroxybutyrate (βHB) and glucose tetrasaccharide (Glc4). |
| **Result (Primary outcome)** | KE + CHO induced acute nutritional ketosis in all six subjects within one hour of ingestion, with median peak βHB concentration of 2.6 mmol/L (range: 1.6–3.1 mmol/L). Glucose concentrations were significantly higher throughout the exercise protocol in the CHO arm (t=50, P<0.0001; t=60, t=105, t=110, P<0.01), with mean delta glucose higher in the CHO arm (4.7 vs 2.6 mmol/L). Heart rate increased in both arms: 70 bpm at rest to 100 bpm at 30% Wmax and 130 bpm at 60% Wmax. RER did not increase in the KE + CHO arm when workload was doubled from 30% to 60% Wmax in subjects with overt myopathy, whereas it returned to 1.0 in the CHO arm. In vivo ³¹P-MR spectra showed a favorable change in quadriceps energetic state during exercise in the KE + CHO arm compared to CHO in subjects with overt myopathy, with a lower Pi/PCr ratio at both 30% and 60% Wmax in subject #1, and prolonged exercise duration in subject #3 (229 s vs 162 s) |
| **Result (Secondary outcome)** | Peak AcAc concentration in the KE + CHO arm was 1.0 mmol/L (range: 0.7–1.2 mmol/L). Insulin concentrations were significantly lower at t=50 and t=105 min in the KE + CHO arm compared to CHO (P<0.05). Lactate concentrations increased from baseline during exercise in both arms but did not differ significantly between arms at any timepoint. Free fatty acid concentrations remained low in both arms, no significant difference between arms. Median RPE scores were comparable between arms at 30% Wmax (7 in both arms) and at 60% Wmax (9 vs 10 in KE + CHO and CHO arms, respectively). Urinary βHB excretion was higher in the KE + CHO arm. Urinary Glc4 concentrations were lower in subjects without overt myopathy |
| **Safety results** | No adverse effects observed. |

| **Klopstock et al; 2000** | |
| --- | --- |
| **Country** | Germany |
| **Study design** | Randomised controlled trial |
| **Age, years** | Mean (SD)**:** Study- 46.4(17.2) |
| **Disease** | Mitochondrial myopathy (MM) or chronic progressive external ophthalmoplegia (CPEO) |
| **Inclusion Criteria** | Ragged red fibers on muscle biopsy |
| **Exclusion Criteria** | - |
| **Patient Characteristics** | Age at onset of symptoms, years [Mean(SD)]: 32.86(15.4) |
| **Intervention** | Creatine monohydrate |
| **Intervention details** | Creatine monohydrate- 5 g orally, 4 times daily |
| **Control** | Placebo |
| **N** | Study= 16, Control= 16 |
| **Follow-up period** | 8 weeks |
| **Outcomes assessed** | **Primary Outcome**: Medical Research Council (MRC) scale for muscle strength; Visual analogue scale for subjective muscle weakness; Maximal voluntary muscle torques of biceps muscle (arm curl) and quadriceps muscle (knee extension) by multifunctional training machine; Hammersmith motor ability score, neuromuscular symptom score, function time test; efficacy and safety  **Secondary Outcomes**: Resting and postexercise lactate by cycle ergometry; ataxia score; neuro-orthoptic examination |
| **Result (Primary outcome)** | No significant treatment effects were found in any of the primary outcome measures. MRC scale showed a positive but non-significant estimated treatment effect of 2.20 (95% CI: −0.82 to 5.22; p=0.13). Hammersmith motor ability score showed an estimated treatment effect of 0.04 (95% CI: −0.93 to 1.01; p=0.93). Maximal biceps torque showed an estimated treatment effect of −1.59 Nm (95% CI: −7.41 to 4.23; p=0.56) and maximal quadriceps torque −11.0 Nm (95% CI: −29.3 to 7.3; p=0.21). Neuromuscular symptom score showed an estimated treatment effect of −0.45 (95% CI: −1.62 to 0.72; p=0.42) and function time test −0.04 seconds (95% CI: −2.88 to 2.80; p=0.98). |
| **Result (Secondary outcome)** | No significant treatment effects were found in secondary outcomes. Nonischemic isokinetic biceps flexion showed an estimated treatment effect of −64.9 J (95% CI: −130.1 to 659.9; p=0.16) and nonischemic isokinetic quadriceps extension 68.2 J (95% CI: −681.2 to 817.7; p=0.84). Ataxia score showed an estimated treatment effect of −0.15 (95% CI: −1.03 to 0.73; p=0.71). No significant differences were found in resting or postexercise lactate, eye movements, or saccade measurements between creatine and placebo arms. |
| **Safety results** | Muscle cramps in 2 patients receiving Creatine monohydrate |

| **Løkken et al; 2020** | |
| --- | --- |
| **Country** | Denmark |
| **Study design** | Randomised trial |
| **Age, years** | Median: Diet 1- 46.5; Diet 2- 42.3; Diet 3- 34.3 |
| **Disease** | McArdle disease |
| **Inclusion Criteria** | 1)Patient is ambulatory 2)Genetically confirmed GSDV |
| **Exclusion Criteria** | 1)Pregnancy or breastfeeding 2) Patients with porphyria or disorders of fat metabolism |
| **Patient Characteristics** | - |
| **Intervention** | Modified ketogenic diet |
| **Intervention details** | One of three diet regimes for 3 weeks- #1 65%/15%/20%; #2 75%/15%/10%; #3 80%/15%/5%, fat/protein/carbohydrate |
| **Control** | - |
| **N** | Study= 10, no controls |
| **Follow-up period** | 3 weeks |
| **Outcomes assessed** | **Primary Outcome:** Exercise capacity by heart rate measured on cycle ergometer; perceived exertion (Borg scale)  **Secondary Outcomes:** Blood ketone levels (AcAc and β-HOB); indirect calorimetry measures (FAO and CHO) during constant workload cycling; and diet compliance |
| **Result (Primary outcome)** | All diet regimes seemed to improve exercise capacity as indicated by a small decrease in Heart rate; Perceived exertion (Borg score) was on average rated lower during the whole exercise period in the post diet condition compared to baseline assessments; Five patients reported subjective symptom relief, all of whom were on diets #2 and #3; Statistical significance not reported. |
| **Result (Secondary outcome)** | Blood ketone levels observed to be the highest on diet #3 and the lowest on diet #1; Indirect calorimetry data showed increased Fatty acid oxidation rates and decreased carbohydrate oxidation rates following all three diet conditions throughout the exercise period; Compliance to diet as maintained through the extent of the study with Diet #2 had the highest acceptability score |
| **Safety results** | Three reported mild fatigue and headache, two reported mild nausea; all resolved spontaneously. No serious adverse effects |

| **Løkken et al; 2021** | |
| --- | --- |
| **Country** | Denmark |
| **Study design** | Randomised controlled trial |
| **Age, years** | Mean (SD): 49.2(15) |
| **Disease** | Mitochondrial Myopathy |
| **Inclusion Criteria** | 1) Ambulatory 2) Genetically verified mitochondrial myopathy, and 3) Symptoms of exercise intolerance |
| **Exclusion Criteria** | 1)Pregnancy or breastfeeding 2) Severe cardiovascular disease 3) Symptoms of secondary mitochondrial myopathy |
| **Patient Characteristics** | - |
| **Intervention** | Resveratrol |
| **Intervention details** | 500 mg twice daily of Resveratrol |
| **Control** | Placebo |
| **N** | Study= 11, Controls= 11 |
| **Follow-up period** | 16 weeks |
| **Outcomes assessed** | **Primary Outcome**: Exercise capacity by heart rate measured on cycle ergometer  **Secondary Outcomes:** Plasma lactate, perceived exertion (Borg scale), modified short form health survey (SF-36); fatigue severity scale score (FSS); changes in indirect calorimetry measures during constant workload cycling, changes in peak maximal workload, and evaluation of changes in mitochondrial biomarkers in muscle tissue; Exploratory outcomes included changes in the blood metabolites pyruvate, free fatty acids (FFA), changes in complex I and complex IV activities in peripheral blood mononuclear cells (PBMC) and changes in the biomarker growth differential factor 15 (GDF-15) |
| **Result (Primary outcome)** | No difference in heart rate measurement on submaximal load on the cycle ergometry between the study and control group observed (ΔHR 0.33 ± 4.27 (RSV) vs 1.82 ± 4.99 bpm (P), P= 0.241) |
| **Result (Secondary outcome)** | No statistically significant change was noted in the secondary or exploratory outcomes between the study and control groups |
| **Safety results** | Mild gastrointestinal symptoms including diarrhea and 3 patients reported joint stiffness in the Resveratrol group, 2 patients reported joint stiffness in the placebo arm. Two patients reported short periods of dizziness 2-3 times in the Resveratrol group |

| **Løkken et al; 2022** | |
| --- | --- |
| **Country** | Denmark |
| **Study design** | Randomised controlled trial |
| **Age, years** | Mean (SD): 42.3 (14) |
| **Disease** | McArdle disease |
| **Inclusion Criteria** | Genetically confirmed McArdle disease or Healthy controls |
| **Exclusion Criteria** | Pregnancy or breastfeeding |
| **Patient Characteristics** | - |
| **Intervention** | Oral ketone ester supplementation |
| **Intervention details** | KE drink with 395 mgKE/kg, containing D-β-hydroxybutyrate esters ((R)-3-hydroxybutyl (R)-3- hydroxybutyrate) (25 g/65 ml), water, stevia, natural flavors, malic acid, potassium, sorbate, and potassium benzoate. |
| **Control** | Placebo- ketone-placebo-flavor-mix (HVMN®), bitter flavor (denatonium benzoate), stevia, and water. |
| **N** | Study= 8, Controls= 4 |
| **Follow-up period** | 25 minutes each day for two days |
| **Outcomes assessed** | **Primary Outcome:** Exercise capacity by heart rate measured on cycle ergometer  **Secondary Outcomes:** Perceived exertion (Borg scale); evaluation of KB (ketone bodies), fat, and CHO oxidation measured via indirect calorimetry and stable isotope technique at rest and during exercise after KE supplementation; changes in plasma concentrations of KBs, insulin, glucose, free-fatty acids (FFA), ammonia, pyruvate, lactate, and amino acids. |
| **Result (Primary outcome)** | Within‐group differences: there was no difference in HR (bpm) during the submaximal exercise test between placebo (P) and KE supplementation, neither in the GSDV (P: 117.9 ± 14.3 vs. KE: 123.0 ± 15.2, *p* = 0.120) or the HC group (P: 114.0 ± 11.2 vs. KE: 115.2 ± 15.9, *p* = 0.567). |
| **Result (Secondary outcome)** | Perceived exertion: no within-group difference in GSDV (P: 11.3±2.0 vs. KE: 12.2±2.8; p=0.109) or controls (P: 9.8±2.0 vs. KE: 9.4±1.5; p=0.678); Plasma HOB significantly increased in GSDV after KE vs. placebo (78.6±55.8 to 3289.9±1327.0 μmol/L; p=0.00008), as did plasma AcAc (p=0.0001) and relative KB oxidation in both GSDV (P: 0.6±0.5 vs. KE: 4.3±1.3 μmol/kg/min; p=0.0001) and controls (P: 1.1±0.4 vs. KE: 5.3±0.7; p=0.00006). Plasma glucose and FFA were lower with KE vs. placebo in GSDV (glucose: p=0.020; FFA: P: 0.47±0.12 vs. KE: 0.25±0.08; p=0.0003), and plasma glycerol Ra was lower indicating reduced lipolysis (P: 5.6±1.7 vs. KE: 3.3±1.1; p=0.007). No significant differences were found between KE and placebo in glucose Ra (GSDV: p=0.628; controls: p=0.265), plasma lactate (p=0.561), pyruvate (p=0.215), or ammonia (p=0.337) in GSDV. |
| **Safety results** | No adverse effects observed. |

| **Løkken et al; 2023** | |
| --- | --- |
| **Country** | Denmark |
| **Study design** | Randomised controlled trial |
| **Age, years** | Mean (SD): 48.6 (12) |
| **Disease** | McArdle disease |
| **Inclusion Criteria** | 1) Ambulatory 2) Genetically verified McArdle disease |
| **Exclusion Criteria** | 1)Pregnancy or breastfeeding 2) Severe cardiovascular disease 3) Significant cardiac or pulmonary disease, porphyria or disorders of fat metabolism |
| **Patient Characteristics** | - |
| **Intervention** | Modified Ketogenic diet (mKD) |
| **Intervention details** | Modified ketogenic diet- 80%/15%/5% fat/protein/carbohydrates |
| **Control** | Placebo- 50%/15%/35% fat/protein/carbohydrates (PD) |
| **N** | Sequence 1= 11, Sequence 2= 9 |
| **Follow-up period** | 3 weeks |
| **Outcomes assessed** | **Primary Outcome:** Exercise capacity by heart rate measured on cycle ergometer  **Secondary Outcomes:** Plasma Ketone Bodies (Aceto-acetate (AcAc) and beta-Hydroxy-Butyrate (b-HOB)), perceived exertion (Borg scale), modified short form health survey (SF-36); fatigue severity scale score (FSS); 5 point questionnaire for patient reported outcomes |
| **Result (Primary outcome)** | It was observed that the mean Heart Rate dropped numerically by -6 and -4 bpm with the mKD and PD, respectively, compared to initial visits. These changes were insignificant (p= 0.10 and p= 0.24, respectively), but there was no difference between the two diets (p= 0.64) |
| **Result (Secondary outcome)** | Significant increase in the ketone bodies on mKD compared to PD (b-HOB (p=0.002) and AcAc (p=0.018)); Decreased perceived perception in mKD ((p= 0.003) and PD (p= 0.15) was observed but no difference between the two dietary groups (p > 0.1); SF-36 scores improved on mKD compared to  PD in all domains (p= 0.008); FSS-score decreased on mKD compared to baseline (p= 0.013), as opposed to no difference on PD compared to baseline (p= 0.27) but statistical between-diet difference in the FSS-score changes observed; Improvement of reported symptoms observed on the simple 5-point questionnaire in mKD vs. PD (p=0.031) |
| **Safety results** | Mild and transient headache, fatigue, nausea and diarrhea/constipation with mKD |

| **Mancuso et al; 2010** | |
| --- | --- |
| **Country** | Italy |
| **Study design** | Randomised controlled trial |
| **Age, years** | Mean (SD): Study- 55.3 (12.9), controls**-** 55.9 (16.5) |
| **Disease** | Mitochondrial myopathies and cytopathies |
| **Inclusion Criteria** | Genetically confirmed diagnosis of Mitochondrial myopathies |
| **Exclusion Criteria** | None |
| **Patient Characteristics** | - |
| **Intervention** | Cysteine donor supplementation |
| **Intervention details** | Whey-based cysteine donor 10 g/day |
| **Control** | Casein 10g/day |
| **N** | Study= 27, Controls= 42 |
| **Follow-up period** | One month |
| **Outcomes assessed** | **Primary Outcome:** MRC (Medical Research Council) scale of muscle strength; Short Form 36, Health Survey (SF-36)  **Secondary Outcomes:** Blood oxidative stress biomarkers [advanced oxidation protein products (AOPP) and ferric reducing antioxidant power (FRAP)] levels |
| **Result (Primary outcome)** | MRC and SF-36 scales were not modified by the 30-day supplementation of the cysteine donor |
| **Result (Secondary outcome)** | Advanced Oxidation Protein Products (AOPP) levels were significantly greater in patients than controls (329.7±122.2 vs 212.0±71.2 mmol/L; p<0.00001). Ferric Reducing Antioxidant Power (FRAP) levels did not differ between patients and controls (0.623±0.151 vs 0.649±0.142 mmol/L). After supplementation: resting FRAP increased (+24.3%; p<0.005); resting AOPP decreased (−50.3%; p<0.00002 vs placebo); AOPP also decreased immediately after exercise (−34.0%; p<0.05) and 15 minutes post-exercise (−40.5%; p<0.02 vs placebo). Resting glutathione increased +50.0% (p<0.00005), with significant increases also immediately after and 15 minutes post-exercise (p<0.05 and p<0.001 vs placebo respectively). |
| **Safety results** | No adverse effects observed. |

| **Ørngreen et al; 2003** | |
| --- | --- |
| **Country** | Denmark |
| **Study design** | Randomised controlled trial |
| **Age, years** | Study: 17,20,23,28,44; Controls- Mean (SD): 24(1) |
| **Disease** | Carnitine palmitoyltransferase II (CPT II) deficiency |
| **Inclusion Criteria** | Diagnosis confirmed by measurements of CPT II activity in cultured skin fibroblasts |
| **Exclusion Criteria** | - |
| **Patient Characteristics** | - |
| **Intervention** | High carbohydrate diet |
| **Intervention details** | High carbohydrate diet- 20% fat, 15% protein, and 65% carbohydrate |
| **Control** | High fat diet- 60% fat, 15% protein, and 25% carbohydrate |
| **N** | Study= 4, Controls= 4 |
| **Follow-up period** | 3 days |
| **Outcomes assessed** | **Primary Outcome:** Exercise tolerance assessed by perceived exertion (Borg scale), endurance (exercise duration), heart rate during constant workload cycling at 50% VO2max  **Secondary Outcomes:** Plasma metabolites (glucose, lactate, FFA, glycerol, alanine), hormones (insulin, norepinephrine), respiratory exchange ratio (RER), oxygen uptake (VO2), and creatine kinase (CK) before and after exercise |
| **Result (Primary outcome)** | Perceived exertion (Borg scale): significantly lower on carbohydrate-rich vs fat-rich diet in the first 26 minutes of exercise (p<0.005); exercise duration: prolonged by 10.7±2.5 minutes on carbohydrate-rich diet in three of four patients; heart rate: lower in two patients on carbohydrate-rich diet (average last 6 min: carbohydrate-rich 124±4 vs fat-rich 126±6 beats/min). |
| **Result (Secondary outcome)** | Resting plasma glucose, lactate, FFA, glycerol, and alanine did not differ between diets; plasma lactate and glucose were unchanged during exercise on both diets; RER and VO2 did not differ at rest and increased to the same extent during exercise on both diets; norepinephrine increased similarly on both diets; CK levels did not differ between diets at baseline or 24 hours post-exercise (carbohydrate-rich: 179±74 vs fat-rich: 164±93). |
| **Safety results** | No adverse effects observed. |

| **Scheffers et al; 2023** | |
| --- | --- |
| **Country** | Netherlands |
| **Study design** | Randomised controlled trial |
| **Age, years** | Mean (SD): Study- 9.735 (5.01); Control- 12.6 (4.12) |
| **Disease** | Pompe disease |
| **Inclusion Criteria** | Children and adolescents (6-18years) with infantile Pompe disease |
| **Exclusion Criteria** | Contraindications to exercising |
| **Patient Characteristics** | Age at onset of symptoms, years [Mean(SD)]: Study- 4.4 (4.5); Control- 2.9(4.3) |
| **Intervention** | High protein diet + physical training (12weeks diet+training vs 6week diet only) |
| **Intervention details** | Tailored exercise program (45-60minutes) and high-protein diet (2g/kg) |
| **Control** | No exercise |
| **N** | Study= 8, Control= 6 |
| **Follow-up period** | 12 weeks |
| **Outcomes assessed** | **Primary Outcome**: Exercise capacity by cardio-pulmonary exercise test (CPET) maximal and submaximal, and by 6 Minute Walk Test (MWT)  **Secondary Outcomes**: Muscle strength by handheld dynamometry, motor function by Quick Motor Function Test (QMFT) and Supine to Stand Test (STST), Quality of Life (QoL) by child health questionnaire (CHQ), fatigue PedsQL Multidimensional Fatigue Scale (MFS), Body composition using skin calipers, Blood Creatine Kinase (CK) levels |
| **Result (Primary outcome)** | Absolute peak VO₂ improved significantly after the intervention (1279 mL/min [1012.5–2006] vs. 1352 mL/min [1101.5–2069]; p=0.039), but not compared to the control period (p=0.643). Peak VO₂/kg did not change. 6MWT walked distance did not improve in the exercise period (456m [348–488] vs. 401m [304–540]; p=0.675) or vs. control period (p=0.857). Submaximal CPET heart rate did not change significantly vs. control period. |
| **Result (Secondary outcome)** | Muscle strength total sum score improved significantly vs. control period (77.7N [66.58–136.9] vs. 84.2N [68.3–153.3]; p=0.001), with significant improvements in hip flexors (p=0.005), hip abductors (p<0.001), elbow flexors (p=0.035), and knee extensors (p<0.001). Core stability improved significantly in all four exercises vs. control period (p≤0.013). Parent-reported QoL improved in physical functioning (p=0.006), change in health, and family cohesion domains; child-reported QoL improved in the change in health domain vs. control period. Parent-reported fatigue improved in general fatigue (10 points; p=0.014) and total fatigue (16 points; p=0.034) vs. control period. Child-reported fear of exercise reduced vs. control period (p=0.043). Quick Motor Function Test (QMFT) score did not reach significance vs. control period (p=0.950). Time to move from supine to standing position (Supine to Stand Test, STST) improved within the exercise period (p=0.015) but not vs. control period (p=0.213). Body composition (BMI and body fat percentage) and CK levels remained unchanged (421 vs. 350 Units/liter; p=0.245). |
| **Safety results** | No adverse effects observed. |

| **Sechi et al; 2020** | |
| --- | --- |
| **Country** | Italy |
| **Study design** | Randomised controlled trial |
| **Age, years** | Mean (SD): Study- 49 ± 11 years |
| **Disease** | Late onset Pompe disease (LOPD) |
| **Inclusion Criteria** | 1)≥ 18 years 2) Enzymatic and/or genetically confirmed LOPD 3) Regular Enzyme replacement therapy for at least 2 years. |
| **Exclusion Criteria** | 1)Presence of significant cardiovascular diseases 2) wheelchair bound 3)24 h ventilator support 4) pregnancy |
| **Patient Characteristics** | Age at onset of symptoms, years [Median]: 37.0 ± 16.0 |
| **Intervention** | High protein diet + physical training (12weeks diet+ training vs 6week diet only) |
| **Intervention details** | Exercise & diet (25–30% protein, 30–35% carbohydrate and 35–40% fat) |
| **Control** | No exercise |
| **N** | Study= 13, Control= 13 |
| **Follow-up period** | 26 weeks |
| **Outcomes assessed** | **Primary Outcome:** Exercise tolerance assessed by peak oxygen uptake (peak VO₂) on a cycle ergometer, 6-minute walk test (6MWT), and quality of life by Short Form 36 Health Survey (SF-36)  **Secondary Outcomes:** Serum enzymes including aspartate aminotransferase (AST), alanine aminotransferase (ALT), lactate dehydrogenase (LDH), and creatine kinase (CK), and pulmonary function tests (PFT) by spirometry including vital capacity (VC) and forced expiratory volume in 1 second (FEV1) |
| **Result (Primary outcome)** | Peak VO₂ decreased significantly after the control period (p=0.004), increased after exercise alone (p=0.05), and improved more significantly after exercise + diet (p=0.009). 6MWT walked distance did not change significantly in any experimental condition (control: 404m vs. 412m, p=0.426; exercise: 425m vs. 420m, p=0.166; exercise + diet: 416.8m vs. 409m, p=1.0). |
| **Result (Secondary outcome)** | LDH decreased significantly after exercise + diet (p=0.047); CK and LDH were both significantly reduced after exercise + diet compared to exercise alone (p=0.03 and p=0.04 respectively). FEV1 improved significantly after exercise + diet (66.5% vs. 85%; p=0.039), with a trend toward improvement in VC (p=0.06); no pulmonary changes were observed after control or exercise alone. QoL showed a trend toward improvement in mental component scale after exercise alone (p=0.07); after exercise + diet, general health (p=0.03) and vitality (p=0.03) improved significantly, with trends in mental health (p=0.07) and mental component scale (p=0.09). BMI decreased significantly after exercise + diet (p<0.05). No changes were found in blood glucose, hemoglobin, lipid profile, albumin, or creatinine throughout the study. Walton scale score did not change in any experimental condition. No significant differences were found in muscle strength of upper or lower limbs in any experimental condition |
| **Safety results** | No adverse effects reported. |

| **Storgaard et al; 2022** | |
| --- | --- |
| **Country** | Denmark |
| **Study design** | Randomised controlled trial (crossover) |
| **Age, years** | Mean (SD): Study- 39 (11.33) years |
| **Disease** | Fatty acid oxidation (FAO) disorders- very long-chain acyl-CoA dehydrogenase (VLCAD) deficiency or carnitine palmitoyl transferase (CPT) II deficiency |
| **Inclusion Criteria** | 1)Genetically verified CPTII or VLCAD deficiencies 2)18 to 80 years 3)Ambulatory 4)signs or symptoms of metabolic myopathy |
| **Exclusion Criteria** | 1)Presence of significant cardiovascular diseases 2)wheelchair bound 3)24 h ventilator support 4)pregnancy |
| **Patient Characteristics** | **-** |
| **Intervention** | Resveratrol (RSV) |
| **Intervention details** | Resveratrol (RSV) 1000mg/day |
| **Control** | Placebo |
| **N** | RSV first = 5, Placebo first= 4 |
| **Follow-up period** | 8 weeks |
| **Outcomes assessed** | **Primary Outcome:** Heart rate and Fatty acid oxidation during constant workload exercise  **Secondary Outcomes:** Perceived exertion (Borg score); fatigue by Fatigue Severity Scale (FSS); Bouchard's diary |
| **Result (Primary outcome)** | Heart rate did not differ at the end of exercise after treatment with RSV vs placebo (P=0.063); Rate of oxidation of palmitate at end of exercise did not improve (P=0.109) |
| **Result (Secondary outcome)** | No improvement in perceived exertion; No improvement in Bouchard's diary (p=0.469) and FSS (p=0.117) |
| **Safety results** | No severe adverse effects observed. 2 participants experienced headache, 1 participant had sleeping of legs, one participant experienced bilateral self-resolving dorsal hand eczema |

| **Tarnopolsky et al; 1997** | |
| --- | --- |
| **Country** | Canada |
| **Study design** | Randomised controlled trial |
| **Age, years** | Mean (SD): Study- 38.6 (11.7) |
| **Disease** | Mitochondrial cytopathies |
| **Inclusion Criteria** | - |
| **Exclusion Criteria** | - |
| **Patient Characteristics** | 1) Elevated resting lactate concentration (>2.7 mmol/L) 2) Severe exercise intolerance 3) Ragged-red fibers on light microscopic examination with significant electron microscopic abnormalities |
| **Intervention** | Creatine Monohydrate (CrM) |
| **Intervention details** | Creatine monohydrate (5 g 2 times a day for 2 weeks followed by 2grams per day for one week) |
| **Control** | Glucose polymer (∼20 kcal) |
| **N** | Study= 7, Control= 7 |
| **Follow-up period** | 3 weeks |
| **Outcomes assessed** | **Primary Outcome:** Activities of daily living (ADL) by visual analogue scale, 2-minute walk test, ischemic isometric handgrip strength by forearm ischemic test (FIT), nonischemic isometric dorsiflexion torque (NIDFT), basal and postexercise lactate, maximal voluntary contraction (MVC) and evoked contraction strength of the dorsiflexors, aerobic cycle ergometry variables including VO₂, RER, heart rate, minute ventilation (VE) and rating of perceived exertion (RPE), and body composition by dual energy X-ray absorptiometry (DEXA). |
| **Result (Primary outcome)** | Ischemic isometric handgrip strength increased by 19% with creatine vs. placebo (p<0.01), with significant increases at t=10s and t=40s (p<0.05). NIDFT increased by 11% overall with creatine vs. placebo (p<0.01), with significant increases at 15s, 30s, and 105s (p<0.05). Postexercise lactate increased significantly immediately after both the forearm ischemic test and cycle ergometry in the creatine trial (p<0.05); resting and pre-exercise lactate did not differ between trials. No significant treatment effects were found for MVC, PTT, TPT, 1/2RT, or M-wave amplitude. No significant treatment effects were found for VO₂, RER, HR, VE, or RPE during cycle ergometry. ADL scores, 2-minute walk test, and body composition (lean body mass, body fat percentage, bone mass density) did not change significantly between trials. |
| **Result (Secondary outcome)** | - |
| **Safety results** | No reported related adverse effects. |

| **Vorgerd et al; 2000** | |
| --- | --- |
| **Country** | Germany |
| **Study design** | Randomised controlled trial (crossover) |
| **Age, years** | Mean (SD): Study- 39.1 ± 17.9 years |
| **Disease** | McArdle disease |
| **Inclusion Criteria** | 1)Exercise intolerance 2)Limited activities of daily living |
| **Exclusion Criteria** | - |
| **Patient Characteristics** | Age at onset of symptoms, years [Mean(SD)]: 27.7 [12.1] years |
| **Intervention** | Creatine Monohydrate (CrM) |
| **Intervention details** | Creatine capsules (150 mg/kg/day for 1 week, followed by 60 mg/kg body weight daily) |
| **Control** | Placebo |
| **N** | Study= 4, Control= 5 |
| **Follow-up period** | 5 weeks |
| **Outcomes assessed** | **Primary Outcome**: Exercise intolerance by cycle ergometer; phosphorus 31 nuclear magnetic resonance spectroscopy for muscle bioenergetics,  **Secondary Outcomes:** Serum creatinine; Electromyograms (EMG); Fatigue Severity Scale (FSS) for fatigue |
| **Result (Primary outcome)** | No significant differences in maximum workload (p=0.68) and in maximal duration of exercise (p=0.70) with CrM; Phosphocreatine depletion increased significantly during aerobic exercise (P = 0.006). Force-time integrals (p=0.03) and depletion of phosphocreatine (p=0.04) increased significantly during ischemic exercise with creatine. |
| **Result (Secondary outcome)** | Serum creatine (p=0.01) increased significantly with CrM; Creatine kinase values did not change significantly with creatine use (p=0.16); Decrease of median frequency in surface electromyograms during contraction was significantly larger (p=0.03) with creatine; FSS scores at the end of the creatine and placebo phases did not differ significantly (p=0.54) |
| **Safety results** | No serious related adverse effects. |

| **Vorgerd et al; 2002** | |
| --- | --- |
| **Country** | Germany |
| **Study design** | Randomised controlled trial (crossover) |
| **Age, years** | Mean (SD): Study- 33.8 [11.8] years |
| **Disease** | McArdle disease |
| **Inclusion Criteria** | 1)Exercise intolerance, such as exercise-induced pain in working muscles and early fatigue 2)Limited in daily activities, such as walking uphill and climbing stairs |
| **Exclusion Criteria** | - |
| **Patient Characteristics** | Age at onset of symptoms, years [Mean(SD)]: 27.7 [12.1] years |
| **Intervention** | Creatine Monohydrate (CrM) |
| **Intervention details** | Creatine capsules (150 mg/kg/day) |
| **Control** | Creatine capsules (60 mg/kg/day) |
| **N** | Study= 10, Control= 9 |
| **Follow-up period** | 5 weeks |
| **Outcomes assessed** | **Primary Outcome:** Patient reported symptoms of exercise intolerance, limitation of daily activities  **Secondary Outcomes:** Serum creatinine and Creatinine Phosphokinase (CPK); Magnetic resonance spectroscopy for muscle bioenergetics; Electromyograms |
| **Result (Primary outcome)** | Increases in the intensity of exercise-induced pain in working muscles observed (mean treatment- induced difference [d], 0.30 in log (score), 95% CI: 0.05-0.55, p=0.02) and increased limitation of daily activities (d, 0.59; 95% CI 0.22-0.97, *p*=0.005) with high dose creatine |
| **Result (Secondary outcome)** | Creatinine (p=<0.01) levels increased and CPK (p=0.22) values remained unaltered with high dose CrM use; No significant changes in magnetic resonance spectroscopy variables; Electromyograms (EMG) revealed a smaller increase in the electromyographic amplitude over time during muscle contraction with Cr use (d, -13.52%/min; 95% CI, -23.71%/min ro -3.34%/min, p=0.01) |
| **Safety results** | Primary outcomes highlight the adverse effects of of high dose creatine supplementation and warrant against the use of high dose creatine |

**Critical Illness Myopathy**

| **Verceles et al; 2023** | |
| --- | --- |
| **Country** | USA |
| **Study design** | Randomised controlled trial |
| **Age, years** | Mean (SD): Study= 62 (9.3), Control=62 (9.3) |
| **Disease** | Critically Ill patients |
| **Inclusion Criteria** | 1)>50year-old) 2) mechanically ventilated participants (≥24 hours) 3) pre-admission Barthel Index of ≥70, 4) ability to follow commands and able to perform physical therapy testing prior to ICU admission. |
| **Exclusion Criteria** | - |
| **Patient Characteristics** | APACHE II Score Mean (SD): Study= 17.8(5.5), Control= 15.4 (7.4) |
| **Intervention** | Neuromuscular electrical stimulation (NMES) with high protein supplementation (HPRO) and physical therapy |
| **Intervention details** | 20 NMES sessions in a 14-day period (1.4 sessions/day), including 10 simultaneously with physical therapy, powdered whey protein supplement containing 1.75g/kg/day. |
| **Control** | Standard Care |
| **N** | Study= 16, Control=23 |
| **Follow-up period** | 14 days |
| **Outcomes assessed** | **Primary Outcome**: Muscle volume and cross-sectional area measured using CT-imaging.  **Secondary Outcome:** Nutritional status, nitrogen balance, delirium, hospital and ICU length of stay, days on mechanical ventilation |
| **Result (Primary outcome)** | Decreased muscle loss in study group as compared to the control group, as seen in lower extremity muscle volume (thigh -21.0% vs. -8.4%, p=0.03; lower leg -15.3% vs. -1.2%, p=0.05) and mean combined thigh cross-sectional area (thigh -26.2% vs. -13.8%, p=0.08) |
| **Result (Secondary outcome)** | Positive nitrogen balance in study group on 3 days (significant on day 9), lesser number of patients positive for delirium (not significant). No significant difference in length of stay or days on ventilation. |
| **Safety results** | No adverse effects observed. |

| **Viana et al; 2021** | |
| --- | --- |
| **Country** | Switzerland |
| **Study design** | Randomised controlled trial |
| **Age, years** | Mean (95% CI)= 64.94(58.88-71.00) |
| **Disease** | Critically Ill patients |
| **Inclusion Criteria** | 1)>18 years 2) on mechanical ventilation, 3) had a central venous catheter,4) a functional gastrointestinal tract, 5) receiving a full treatment and were assessed likely to survive more than 7 days. |
| **Exclusion Criteria** | 1)Major burns (>20% body surface), 2) admission for cardiorespiratory arrest or brain injury, 3) pregnancy or lactation, 4) 5patient on parenteral nutrition, 5) participation in another interventional trial and 6) absence of consent. |
| **Patient Characteristics** | SOFA score Mean (95% CI): Day 4= 8.53(7.39-9.68), Day 15=4.41(2.99-5.83) |
| **Intervention** | HMB supplementation |
| **Intervention details** | 3 g per day of β−hydroxy-β−methylbutyrate (HMB) |
| **Control** | Placebo (maltodextrin) |
| **N** | Study= 15, Control=15 |
| **Follow-up period** | 30 days |
| **Outcomes assessed** | **Primary Outcome**: Magnitude of loss of skeletal muscle area (SMA) of the quadriceps femoris measured by ultrasound at days 4 and 15.  **Secondary Outcome:**  Measurements at day 4 and 15 of the phase angle measured by multi frequency bioimpedance (BIA), amino acid metabolism using amino acid tracers, and global health and mobility assessed by the SF-12 questionnaire at 30 and 60 days. |
| **Result (Primary outcome)** | The loss of total SMA was 11% between days 4 and 15 (p < 0.001), but not different between the groups (p = 0.86). |
| **Result (Secondary outcome)** | Net protein breakdown (Δ Estimate HMB-Placebo: −153 [-242, −63]; p = 0.0021) and production of several amino acid was significantly reduced, while phase angle increased more (0.66 [0.09, 1.24]; p = 0.0247), and SF-12 global health improved more (Δ Estimate HMB-Placebo: 27.39 [1.594, 53.19], p = 0.04) in study group. |
| **Safety results** | No adverse effects observed. |

| **Supinski et al; 2021** | |
| --- | --- |
| **Country** | USA |
| **Study design** | Randomised controlled trial |
| **Age, years** | Mean (95% CI): Control- 55.5 (47.7-63.1), EPA= 52.9(46.0-59.7), HMB=61.2(54.0-68.4), HMB+EPA= 56.2(49.7-62.7) |
| **Disease** | Adult mechanically ventilated patients |
| **Inclusion Criteria** | 1)All adult patients requiring mechanical ventilation for more than 48 h for respiratory failure |
| **Exclusion Criteria** | 1)Physician caring for the patient determined that the patient was too unstable to tolerate these measurements; 2) Receiving high dose pressors (more than 15 mcg/min of norepinephrine or more than 15 mg/kg/min of dopamine), 3) required > 80% FiO2 or > 15 cm H2O of PEEP, 4) had a cardiac pacemaker or implanted defibrillator, 5) received neuromuscular blocking agents within the 48 h preceding testing, 6) history of a preexisting neuromuscular disease, 7) recent variceal bleeding, 8) pregnant, 9) incarcerated, 10) institutionalized, or 11) if the attending physician thought that the patient was terminal and would have care withdrawn within 7 days. |
| **Patient Characteristics** | SOFA score: Mean (95% CI): Control- 7.0(5.3-8.7), EPA= 7.7(6.0-9.4), HMB=6.7(5.3-8.1), HMB+EPA= 8.1(6.1-10.1) |
| **Intervention** | Beta-hydroxy-beta-methylbutyrate (HMB) and/or eicosapentaenoic acid |
| **Intervention details** | HMB 3 gm/day and EPA 2 gm/day and HMB plus EPA |
| **Control** | Placebo |
| **N** | Study: EPA = 17, HMB=18, HMB+EPA=18, Control=20 |
| **Follow-up period** | 11 days |
| **Outcomes assessed** | **Primary Outcome**: Diaphragm strength assessed as the trans-diaphragmatic pressure generated by supramaximal magnetic phrenic nerve stimulation (PdiTw), Quadriceps strength assessed as leg force generated by supramaximal magnetic femoral nerve stimulation (QuadTw), Diaphragm and quadriceps thickness assessed by ultrasound. |
| **Result (Primary outcome)** | No significant differences were found across the four treatment arms for any primary outcome measures. ANCOVA analyses revealed no significant difference in diaphragm twitch pressure (PdiTw) (p=0.89) or diaphragm thickness (p=0.50). Similarly, no significant difference was found in quadriceps twitch force (QuadTw) (p=0.46) or quadriceps thickness (p=0.43). The mean difference in QuadTw pre- and post-treatment was 2.26 N (95% CI −0.51 to 5.0), 1.12 N (95% CI −0.34 to 2.59), 0.26 N (95% CI −3.01 to 3.57), and 0.18 N (95% CI −2.07 to 2.43) for Control, EPA, HMB, and HMB+EPA groups respectively. Duration of mechanical ventilation did not differ significantly between groups (p=0.372). |
| **Result (Secondary outcome)** | - |
| **Safety results** | No adverse effects observed. |

**Supplementary Figure S1: Risk of Bias of included studies categorised by disease (traffic light plot)**
